# Single-dose psilocybin-assisted therapy in major depressive disorder: A placebo-controlled, double-blind, randomised clinical trial

**DOI:** 10.1016/j.eclinm.2022.101809

**Published:** 2022-12-28

**Authors:** Robin von Rotz, Eva M. Schindowski, Johannes Jungwirth, Anna Schuldt, Nathalie M. Rieser, Katharina Zahoranszky, Erich Seifritz, Albina Nowak, Peter Nowak, Lutz Jäncke, Katrin H. Preller, Franz X. Vollenweider

**Affiliations:** a Neurophenomenology of Consciousness Lab, Department of Psychiatry, Psychotherapy and Psychosomatics, Psychiatric Hospital, University of Zurich, Zürich, Switzerland; b Department of Psychiatry, Psychotherapy and Psychosomatics, Psychiatric Hospital, University of Zurich, Zürich, Switzerland; c Division Neuropsychology, Department of Psychology, University of Zürich, Zürich, Switzerland

**Keywords:** Psilocybin, Psychedelic-assisted therapy, Major depressive disorder, Depression, RCT, Efficacy, Placebo-controlled

## Abstract

**Background:**

Psilocybin has been suggested as a novel, rapid-acting treatment for depression. Two consecutive doses have been shown to markedly decrease symptom severity in an open-label setting or when compared to a waiting list group. To date, to our knowledge, no other trial compared a single, moderate dose of psilocybin to a placebo condition.

**Methods:**

In this double-blind, randomised clinical trial, 52 participants diagnosed with major depressive disorder and no unstable somatic conditions were allocated to receive either a single, moderate dose (0.215 mg/kg body weight) of psilocybin or placebo in conjunction with psychological support. MADRS and BDI scores were assessed to estimate depression severity, while changes from baseline to 14 days after the intervention were defined as primary endpoints. The trial took place between April 11th, 2019 and October 12th, 2021 at the psychiatric university hospital in Zürich, Switzerland and was registered with clinicaltrials.gov (NCT03715127).

**Findings:**

The psilocybin condition showed an absolute decrease in symptom severity of −13.0 points compared to baseline and were significantly larger than those in the placebo condition (95% CI −15.0 to −1.3; Cohens' *d* = 0.97; P = 0.0011; MADRS) and −13.2 points (95% CI; −13.4 to −1.3; Cohens’ *d* = 0.67; P = 0.019; BDI) 14 days after the intervention. 14/26 (54%) participants met the MADRS remission criteria in the psilocybin condition.

**Interpretation:**

These results suggest that a single, moderate dose of psilocybin significantly reduces depressive symptoms compared to a placebo condition for at least two weeks. No serious adverse events were recorded. Larger, multi-centric trials with longer follow-up periods are needed to inform further optimisation of this novel treatment paradigm.

**Funding:**

The study was funded by the 10.13039/100000001Swiss National Science Foundation, Crowdfunding, the Swiss Neuromatrix Foundation, and the 10.13039/100013057Heffter Research Institute.

**Research in context**

**Evidence before this study**

“Psilocybin”, “Depression” and “MDD” were used as search terms in PubMed up to July 13th 2022. Apart from numerous review articles, population analysis and opinion papers, we identified three trials investigating efficacy of psilocybin in Major Depressive Disorder: An open-label trial, a waiting-list controlled RCT and an RCT comparing psilocybin efficacy to SSRI treatment. None of the trials compared a single, moderate dose of psilocybin to a placebo comparator.

**Added value of this study**

We show that a single, moderate dose of psilocybin is favourable to a placebo in terms of efficacy for at least 2 weeks following drug administration and was well-tolerated by all participants. As the first placebo-controlled study on the efficacy of psilocybin-assisted therapy in MDD, it furthermore demonstrates that the improvements in depressive symptoms shown in previous clinical trials cannot be attributed to the non-pharmacological therapeutic embedding alone. Tolerability of the treatment was favourable in the present trial compared to previously conducted studies using repeated and higher doses, while effect sizes for efficacy remained comparable. The data collected here can therefore help to inform future improvements of dose regimens within the framework of psychedelic-assisted therapy towards an optimised ratio between efficacy and tolerability.

**Implications of all the available evidence**

Existing data on safety and efficacy uniformly suggest that psilocybin-assisted therapy could emerge as an additional option for existing treatments of depression. However, larger cohorts sampled within multi-center trials are warranted to shed further light on predictive psychological and neural mechanism(s) of action, dose–response relationship, and the potential expansion of indications.

## Introduction

Major depressive disorder (MDD) is a leading cause of disability worldwide, affecting more than 300 million people around the globe.^1^ Most currently available antidepressants target the monoaminergic neurotransmission, mainly the serotonin (5-HT) system. However, the efficacy of conventional antidepressants is limited, with a substantial number of patients failing to achieve remission (60–70%).^2^ They are associated with delayed response onset of weeks to months of treatment, high incidence of side effects, high relapse rates, and the necessity for chronic administration. Thus, there is a clear need for more effective and rapid-acting antidepressants.^3^

In the search for new treatments, a pilot study and two controlled clinical trials have shown that the psychotropic substance psilocybin - a preferential serotonin 1A/2A receptor agonist – in combination with psychological support, rapidly and sustainably alleviates depressive symptoms in MDD. Specifically, in an open-label feasibility study, administration of two doses of psilocybin (10 mg and 25 mg, 7 days apart) together with psychological support resulted in rapid symptom improvement in patients with treatment-resistant depression that persisted at a 6 months follow-up.^4^^,^^5^ A waiting-list controlled clinical trial, administering two doses of psilocybin (25 and 30 mg, 2 weeks apart) reported large effect sizes with clinically significant response rates of 71% 1 week post-treatment and sustained therapeutic effects up to 12 months after the intervention.^6^^,^^7^ Additionally, two 25 mg doses of psilocybin (3 weeks apart) plus daily placebo were tested against daily doses of a standard-of-care SSRI (escitalopram) plus two 1-mg doses of psilocybin (3 weeks apart) over a period of 6 weeks. Participants in both treatment conditions showed substantial reduction of depressive symptoms. However, although favouring psilocybin over escitalopram on secondary outcomes, the primary efficacy endpoint (self-reported Inventory of Depressive Symptomatology) did not reach statistical significance.^8^ Given that a rapid decrease in symptom severity was observed in both intervention arms, it is possible that the psychological support and the low dose of psilocybin may have contributed significantly to the therapeutic effect. Lastly, a multi-centric trial investigating efficacy of 25 mg, 10 mg, and 1 mg of psilocybin in patients with treatment-resistant depression reported significant symptom reduction for the 25 mg treatment condition 3 weeks after baseline.^9^

However, no placebo-controlled trial has been published to date, therefore limiting our understanding of the contribution of different pharmacological and non-pharmacological aspects of psychedelic-assisted therapy. Furthermore, all cited clinical trials used dosage regimens that are considered low (≤10 mg) or included a high dose (>20 mg), while omitting the moderate dose range. It is still unknown, whether a single moderate dose of psilocybin may be sufficient to significantly alleviate MMD symptoms.

Therefore, the primary objective of this randomised clinical trial was to investigate the effect of a single moderate dose of psilocybin compared to placebo (equal time spent with psychological support in both treatment conditions) in patients with MDD. Given that moderate doses of psilocybin produced substantial psychological and neurobiological changes in a number of mechanistic studies in healthy participants, we hypothesise that a single, moderate dose of psilocybin is more efficacious than a placebo control.^10^^,^^11^ Seven visits over the course of 3 weeks provide the temporal resolution to capture rapid antidepressant effects and at the same time ensure appropriate psychological support during the preparation, drug, and integration sessions of the experience.

## Methods

### Study design

The trial adopted a randomised, double-blind, placebo-controlled, parallel-groups design including a total of seven in-person visits for each participant. The study was conducted as a single-centre trial between April 11th, 2019 and October 12th, 2021 at the Psychiatric University Hospital Zurich, Switzerland and was conducted in accordance with the Revised Declaration of Helsinki (2000) and the International Council for Harmonisation Good Clinical Practice (GCP) guidelines. Approval was granted by the Cantonal Ethics Committee, Zurich, Switzerland, the Swiss Agency for Therapeutic Products (Swissmedic) and the Federal Office of Public Health (FOPH).

### Participants

The Mini International Neuropsychiatric Interview (M.I.N.I.) was used to confirm the presence of a depressive episode in the context of a major depressive disorder (MDD) and to detect possible comorbid psychiatric conditions. MDD symptom severity was quantified by the Montgomery-Åsberg Depression Rating Scale (MADRS) and Beck's Depression Inventory (BDI). Inclusion criteria included a MADRS score between 10 and 40 points. Furthermore, participants taking medication were required to discontinue psychiatric medication supervised by a psychiatrist for at least 2 weeks or five half-lives of their current antidepressant medication before administration day. Information about prescription medication used by participants at the medical screening visit is summarised in Table 1. Past and acute suicidal tendencies were assessed to confirm credible absence of suicidal ideation (any form of intention or plan) as required by inclusion criteria. Further criteria consisted of an age range of 20–60 years, German language on level C2, willingness to refrain from using psychotropic substances including alcohol during the course of the study and the availability of a close relative to ensure safe return home at the end of the administration day.

**Table 1:** Demographic information of N = 52 participants.

	Psilocybin (N = 26)	Placebo (N = 26)
Age	37.6 (10.9)	35.9 (9.80)
Verbal IQ	115 (13.1)	113 (12.1)
Years of education	14.4 (2.52)	15.0 (2.39)
Gender		
Female	16 (61.5%)	17 (65.4%)
Male	10 (38.5%)	9 (34.6%)
Ethnicity		
White	26 (100%)	23 (88.4%)
Black Caribbean	0 (0%)	1 (3.8%)
Arab	0 (0%)	2 (7.7%)
Clinical		
MADRS [0–60]	24.3 (5.07)	24.1 (7.07)
BDI [0–63]	26.9 (6.70)	25.8 (9.59)
CGI Severity [2–8]	5.50 (0.58)	5.54 (0.91)
Suicidal ideation [0–5]	0.53 (0.85)	0.46 (0.65)
Meds at baseline		
Mean (SD) # antidepressants	0.42 (0.90)	0.27 (0.45)
# of participants with 0	19 (73%)	19 (73%)
# of participants with 1	5 (19%)	7 (27%)
# of participants with >1	2 (7.7%)	0 (0.0%)
Mean (SD) # prescription meds	0.69 (0.93)	0.38 (0.70)
# of participants with 0	14 (54%)	19 (73%)
# of participants with 1	8 (31%)	4 (15%)
# of participants with >1	4 (15%)	3 (12%)
Prior psychedelic experiences		
# of participants with prior experience	5 (19.2%)	11 (42.3%)
Mean (SD) life-time occassions with a psychedelic agent from pre-experienced individuals	2.40 (1.52)	2.00 (1.00)

Verbal IQ was assessed by the Multiple-Choice Word Test (MWT-B). Clinical Global Impressions (CGI) provides a clinician-rated impression of overall severity of clinical symptomatology. Suicidal ideation is derived from the intensity of ideation subscale from the Colombia Suicidality Severity Rating Scale (C-SSRS) assessing suicidal thoughts with a scale ranging from zero to five whereas scores greater than three imply acute intentionality. The number of medications used was assessed at the medical screening before discontinuation. Prescription medications include all pharmaceuticals only available on prescription, excluding antidepressant medication which is reported separately. Percentages may not sum up to 100% due to rounding.

Exclusion criteria consisted of: Psychosis spectrum disorders and/or mania symptoms in participants or first-degree relatives. Alcohol or other drug dependence according to DSM-V was not permitted to be prevalent for three months prior to inclusion. Comorbid anxiety disorders without tendencies for panic attacks were allowed as long as they did not require current pharmacological treatment. In addition, the SCID II was used to screen for axis-II disorders. Symptoms associated with an increased risk of adverse emotional and/or behavioural reactions to the treatment intervention led to exclusion from the study.

In addition to mental health assessments, a physical health check was performed to ensure good somatic health with no unstable medical conditions, covering general somatostatus, electrocardiogram, blood pressure, heart rate, routine blood laboratory and urine drug analysis (drug screening and pregnancy test). Women with childbearing potential were additionally required to use medically accepted contraception methods. No more than ten prior experiences with any hallucinogenic compound were allowed to support reasonable standardisation.

The trial was conducted in German including the use of corresponding validated versions of questionnaires and interviews. Written informed consent was obtained from all participants.

### Randomisation and masking

After enrolment of a participant by a study physician, the resident pharmacy was responsible for randomisation and allocation of the participant to either the psilocybin-assisted therapy group or the placebo-therapy group according to a priori computer-generated sequence in a 1:1 ratio without further stratification. Each participant was randomised after their third visit (−1d). The resident pharmacy had no further role in the rest of the trial. Allocations were stored in sealed, opaque envelopes. Placebo capsules were identical to the psilocybin-containing capsules in size, weight, shape and colour and contained pure mannitol. Pharmaceutical-grade, synthetic psilocybin was obtained from Compass Pathways, Ldt. Participants and study personnel except the resident pharmacist were blind to participants' treatment allocation until after the database was locked.

### Procedures

On the administration day (visit 4), the psilocybin-assisted therapy treatment group received 0.215 mg/kg body weight psilocybin in white gelatine capsules including either 1 or 5 mg of the active pharmacological ingredient implying the necessity of rounding (e.g., a participant with 70 kg body weight would receive 15.05 mg, subsequently rounded up to 16 mg = 3 × 5 mg + 1 × 1 mg). This dose range corresponds to a moderate psychoactive dose.^12^^,^^13^ Notably, compared to other published clinical trials using psilocybin for the treatment of depression, the dose used in this study is lower and was only administered once (compared to two administrations in previous studies).^6^^,^^9^

After medical screening, participants completed two preparatory visits (visits 2 and 3) 4–6 and 1 day before psilocybin/placebo administration. Subsequently, participants completed three integration visits (visit 5, 6, and 7) 2 days, 8 days, and 14 days after the intervention to provide psychological support throughout the study. Psychological counselling was scheduled at every study visit for ∼60 min. These sessions ensured a) psychological safety and well-being of all participants, b) similar to other clinical trials, patients were informed about the purpose of the study, i.e., investigating the clinical potential of psilocybin-assisted therapy for MDD, and were educated about potential mechanisms of action (visits 2 and 3), c) preparation for an experiential treatment including setting an intention for the experience and its potential outcomes (visit 3), d) a trusting relationship with the therapist accompanying the administration day fostering openness and acceptance with regard to the experience (visit 4), e) guidance on the integration of the experience including working through challenging emotions (visit 5) as well as facilitating the creation of a meaningful narrative thereof (visit 6) and f) support for adequate behavioural adaptations in everyday life (visit 7).

During drug administration visits, participants were instructed to immerse themselves in the experience with an introspective focus. A standardised playlist with music was played via headphones or speakers. One trained therapist was present in the room throughout the administration day to respond to the participants' needs. All visits were conducted in a living-room like environment. The duration of psychological counselling consisted of 2 h of preparation, 6 h during administration day, and 3 h of integration resulting in a total duration of 11 h.

### Choice of primary measure

Two independent scales were administered at every study visit to assess MDD symptom severity and response rates: MADRS and BDI. The primary endpoints were defined as changes from visit 2 (−5d) to visit 7 (+14d) on these scales. The MADRS, a clinician-rated instrument, is one of the most commonly used symptom severity scales to evaluate the efficacy of antidepressant treatment. A clinically relevant response is defined as either a 50% reduction in the MADRS sum score or a decrease below a prospectively determined threshold of <10 points or both.^14^ The BDI is one of the most commonly used self-report questionnaires. The cut-off for remission is <10 points and the criteria for clinically relevant responses are calculated analogously to the MADRS.^15^

### Secondary measures

Secondary outcome measures provide additional insight into further somatic and psychiatric symptoms. The following secondary outcomes were assessed:

a) The SCL-90-R assesses subjective impairments that emerged in the last seven days prior to assessment. It was administered at medical screening and at visit 7 (+14d). b) The Hamilton-Anxiety Rating Scale (HAM-A) clinician-rated scale evaluating anxiety states and changes and was assessed at every study visit. c) The Clinical Global Impression (CGI) is a widely used instrument estimating the overall severity of psychopathology and was administered at each study visit. d) The Colombia - Suicidality Severity Rating Scale (C-SSRS) was used for the quantification of suicidal ideation and early detection of potential suicidal behaviour. e) The Altered States of Consciousness (ASC) questionnaire, one of the most commonly used self-rating questionnaires to quantify subjective drug effects, was administered at the end of the administration day (∼360 min after administration). All scale scores are given as a percentage of the maximum scale value. The global intensity score captures the overall extent of consciousness alterations and is calculated as the mean of all main dimensions excluding vigilance reduction. Higher scores indicate greater intensity of alterations. For the details on the calculation of subscales derived from these questionnaires, please see Supplement.

To ensure adequate monitoring of safety-related parameters, the following endpoints were assessed at each study visit: Adverse events (AEs), psychological and physical well-being, suicidality, vital signs, and the use of concomitant medication. Tolerability of acute drug effects was monitored by hourly evaluations of blood pressure and pulse. Rate-pressure products enabling quantification of hemodynamic response were obtained by multiplying systolic blood pressure with heart rate.^16^ Reporting of AEs comprised clinically relevant symptoms outlasting acute drug effects, i.e., excluding transient adverse symptoms caused by psilocybin which are described in detail elsewhere.^4^^,^^10^ In case of emergency during acute drug effects nifedipine (10 mg) for hypertension, diazepam (5–10 mg) for severe anxiety and olanzapine (5–10 mg) were prepared for every administration day. Study staff was instructed to attenuate adverse reactions by psychological support before administering rescue medication. No rescue medication was used during the course of the trial.

### Statistical analysis

Data analysis was performed on the intention-to-treat population (ITT), i.e., all participants who received psilocybin treatment and had at least one assessment of efficacy post-treatment, resulting in a total of 52 participants in the sample. The last observation carried forward method was used to impute missing values from the three patients lost to follow-up.

The efficacy analysis on the primary endpoint (depression severity score: MADRS, BDI) consisted of two mixed models analysis of covariance (ANCOVA) including post-treatment values of both efficacy endpoints as dependent variable, treatment condition as independent variable, and pre-treatment values as covariate.

Treatment response and thresholds for remission were derived according to established guidelines.^14^^,^^15^ Responders were defined using the MADRS response criterion (−50% symptom reduction between visit 2 (−5d) and visit 7 (+14d) and/or decrease below the remission threshold. Statistically significant associations between treatment conditions and response-rates were derived using Fisher's exact test (two-sided).

Significant interaction terms between treatment condition and visit number from mixed models of variance including study visit as within-subject factor and treatment condition as between-subject factor, lead to subsequent post-hoc testing. Generalised eta squared (η^2^_G_) is considered the appropriate measure to report the amount of variance explained by the model.^16^ Two-sample Welch's t-tests were used to compare treatment conditions at each time point. Cohen's *d* was used to estimate effect sizes of group differences at visit 7 (+14d). All statistical tests used *P* < 0.05, two-tailed to determine statistical significance. For detailed procedures regarding statistical analysis of secondary endpoints, model assumptions, and power analysis see Supplement. Statistical analysis was performed with R Studio (Version 2021.9.2.382). For packages utilised for statistical tests, see Supplement. Monitoring of study data was independently carried out by the Clinical Trial Unit (CTU) Basel, Switzerland. The study is registered on clinicaltrials.gov (Identifier: NCT03715127) and KOFAM (Identifier: SNCTP000003139).

### Role of the funding source

The study funders had no role in study design, data collection, data analysis, data interpretation, or writing of the report. All authors had full access to all data and accept responsibility for the decision to submit for publication.

## Results

**Fig. 1:**
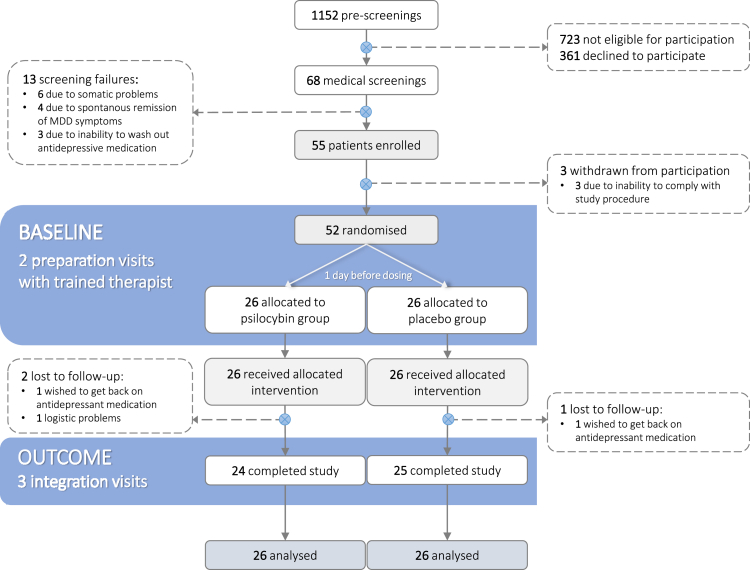
**CONSORT flow diagram and study design.** After enrolment, all participants underwent two preparation sessions (4–6 days and 1 day prior to administration) before being randomised to either the placebo or the psilocybin condition. Drug administration took place on the administration day at 9 a.m. and participants were discharged between 3 a.m. and 5 p.m. on the same day after acute drug effects had completely worn off. Integration of the experience was conducted 2 days, 8 days and 14 days after administration.

1152 potential participants were pre-screened either via mail or telephone. 68 participants with an acute depressive episode were invited to medical screening. 55 participants met all criteria for inclusion and were subsequently enrolled into the trial. Three participants withdrew from participation before being randomised due to the Covid-19 pandemic. A total of 52 participants underwent drug administration. Three additional participants withdrew from further participation: one participant due to Covid-19 related issues (psilocybin group) and two (one in each group) wanted to get back on antidepressant medication resulting in exclusion from the trial. Missing values of these participants were imputed as the last observation carried forward for the primary efficacy analyses (see Fig. 1). For details on demographic information see Table 1.

Change in symptom severity between visit 2 (4–6d before drug administration) and the last study visit (14d after drug administration) was a priori defined as primary efficacy endpoint. Mixed-effects model of covariance showed a significant main effect for treatment condition assessed for MADRS: F(1,49) = 14.20, P = 0.0004, η^2^_G_ = 0.224, and for BDI: F(1,49) = 8.53, P = 0.005, η^2^_G_ = 0.148. Significant interaction effects between study visits were found for MADRS: F(1,49) = 13.30, P = 0.0006, η^2^_G_ = 0.213, and for BDI: F(1,49) = 16.1, P = 0.0002, η^2^_G_ = 0.248. The psilocybin condition showed an absolute decrease in symptom severity of −13.0 points compared to baseline and were significantly larger than those in the placebo condition (95% CI −15.0 to −1.3; Cohens' *d* = 0.97; P = 0.0011; MADRS) and −13.2 points (95% CI; −13.4 to −1.3; Cohens’ *d* = 0.67; P = 0.019; BDI) 14 days after the intervention.

Response rates 14 days after administration resulted in 58% for MADRS (Psilocybin: 15/26 vs. Placebo: 4/26; P = 0.0034) and for BDI in 54% (Psilocybin: 14/26 vs. Placebo: 3/26; P = 0.0025. Remission rates were reported in 54% of patients for MADRS (Psilocybin: 14/26 vs. Placebo: 3/26; P = 0.0023) and assessed by BDI in 46% (Psilocybin: 12/26 vs. Placebo: 3/26; P = 0.013).

**Fig. 2:**
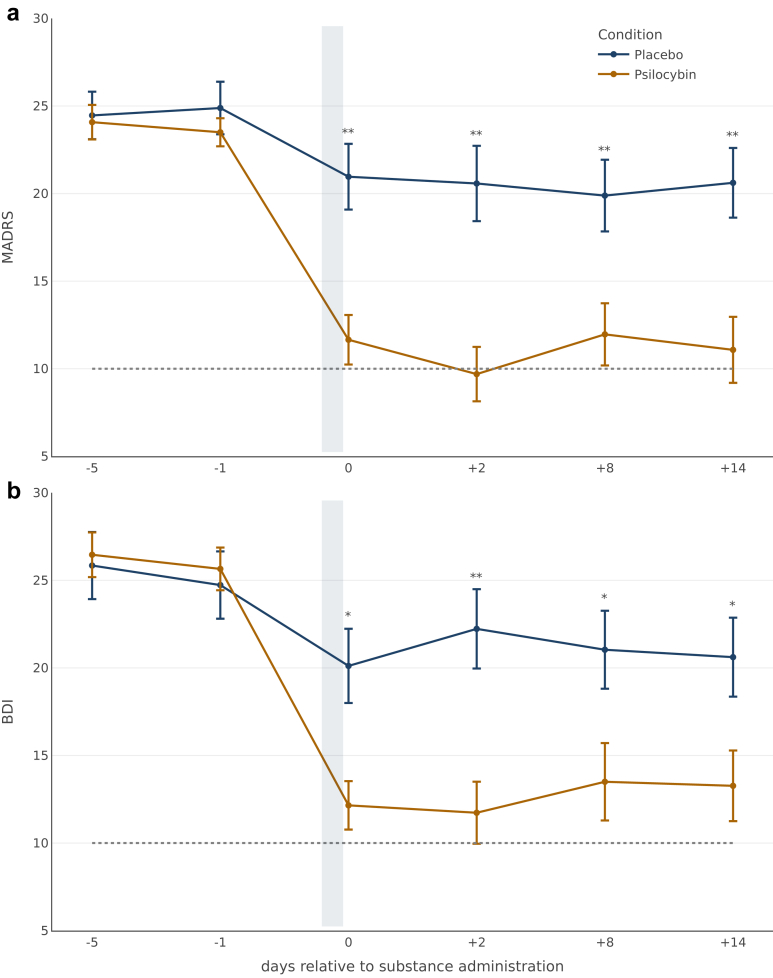
**Mean trajectories of MADRS and BDI at every study visit for both treatment conditions. a:** mean scores of clinician-rated primary endpoint (MADRS); **b:** mean scores of self-reported primary endpoint (BDI). The grey bar depicts the time of substance administration. The endpoints reported for the administration day were assessed after all subjective effects had worn off (around 6 h after administration). The dotted line depicts thresholds defined for the remission of symptoms. Differences between treatment conditions (blue = psilocybin; yellow = placebo) at each time point were calculated using independent two-samples Welch's t-tests rejecting the null-hypothesis at significance levels of ∗P < 0.05; ∗∗P < 0.01; ∗∗∗P < 0.001. Error bars represent standard errors of means (se).

To evaluate the time course of symptom change, analyses including all study visits were conducted for each primary endpoint (Fig. 2). There was a statistically significant interaction between treatment condition and time for MADRS: F(5,250) = 11.8, P < 0.0001, η^2^_G_ = 0.058, as well as for BDI: F(5,250) = 10.4, P < 0.0001, η^2^_G_ = 0.050. Post-hoc analysis confirmed significant differences (P < 0.05) between conditions at each timepoint following substance administration. Mean differences in depressive symptomatology between treatment conditions were highest two days after drug administration (visit 5) for MADRS (−14.4 points; CI −5.5 to −16.3; P = 0.0002; Cohens' *d* = 1.14) and for BDI (−14.7 points; CI −4.7 to −16.3; P = 0.0007; Cohens’ *d* = 1.01). Reported response rates were highest for self-reported symptoms on the administration day with 73% (19/26; BDI) of participants in the psilocybin condition, although the clinician-rated instrument showed the highest response rates 2 days later with 69% (18/26; MADRS) of participants in the psilocybin condition. Individual symptom trajectories are presented in Supplemental Fig. S1.

Secondary endpoints are summarised in Supplemental Table S1. Mixed-effects models of covariance yielded significant main effects for treatment condition for the following subscales: anxiety F(1,46) = 4.70; P = 0.035; depression F(1,46) = 8.70; P = 0.0050; psychoticism F(1,46) = 9.29; P = 0.0004; phobic anxiety F(1,46) = 4.90, P = 0.032, and paranoid ideation F(1,46) = 4.29; P = 0.044 as well as for the global severity index F(1,46) = 6.43; P = 0.015 indicating stronger decreases of symptoms in the psilocybin condition than the placebo condition. Furthermore, significant interactions were found for the HAM-A total score F(1,50) = 5.16; P = 0.027, the CGI severity score F(1,50) = 11.3; P = 0.0010 but not for the intensity of suicidal ideation F(1,50) = 1.40; P = 0.24.

Cardiovascular safety endpoints consisted of: 1) heart rate, 2) systolic and diastolic blood pressure [mmHg], and 3) rate pressure product (RPP). Heart rate was not different between the conditions throughout the 7 h of assessment P > 0.05. Mean systolic and diastolic blood pressure were highest 60 min post-intake (+14.5 mmHg; t(41.6) = −2.96; CI 4.6–24.4; P = 0.0051 for systolic blood pressure and +12.5 mmHg; t(40.1) = −5.09; CI 6.1–18.9; P = 0.0003 for diastolic blood pressure in the psilocybin condition. Systolic blood pressure was statistically different from baseline (30 min pre-intake) up to 5 h post-intake while diastolic blood pressure was significantly higher than at baseline up to 4 h after drug administration in the psilocybin condition. RPP was significantly higher than baseline 60 min after administration in the psilocybin condition compared to placebo: t(45.9) = −2.39; CI −2638 to −244; P = 0.021. Results are visualised in Supplemental Fig. S2.

No rescue medication was used during the course of the trial. No events of symptomatic hypertension, extreme anxiety and psychotic/delusional decompensation were documented. A total of 11 adverse events (i.e., clinically relevant symptoms outlasting acute drug effects) were recorded (Supplemental Table S2). All four cases of headache and the two cases of dizziness were categorised as “likely related” due to the temporal relationship to the intervention. All cases were reported to be mild. The case of diarrhea was already prevalent one day before drug administration, slightly intensified and continued thereafter and was thus categorised as “probably related”. The two cases of common cold and the case of cystitis were classified as “unlikely to be related” to the intervention.

**Fig. 3:**
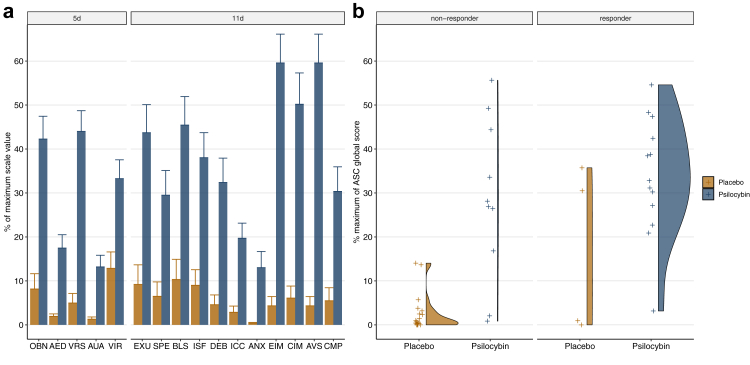
**Association of treatment response with subjective effects. a:** Mean values and standard errors of the mean (error bars) on the 5 dimensions altered states of consciousness questionnaire (ASC) in the psilocybin and placebo groups. Treatment groups (blue) differed significantly from Placebo (yellow) on all dimensions. OBN = oceanic boundlessness; AED = anxious ego dissolution; VRS = visual restructuralization; AUA = auditory alterations; VIR = vigilance reduction. 11 dimensions: EXU = experience of unity; SPE = spiritual experiences; BLS = blissfulness; ISF = insightfulness; DEB = disembodiment; ICC = impaired cognition and control; ANX = anxiety; EIM = elementary imagery; CIM = complex imagery; AVS = audio-visual synesthesia; CMP = changed meaning of percepts. **b:** ASC global intensity scores for responders and non-responders in the psilocybin and placebo groups. Responder and non-responders were defined according to the MADRS response criterion (−50% symptom reduction and/or decrease below the remission threshold) for changes between visit 2 (−5d) and visit 7 (+14d). Distributions were obtained using a gaussian density estimator kernel.

Subjective drug effects were characterised using the ASC questionnaire. Significant interactions between scale and treatment condition were obtained for the 5 main dimensions: F(2.67,130.79) = 11.943; P < 0.0001; η^2^_G_ = 0.099, as well as for the 11 sub-dimensions: F(9,441) = 9.646; P < 0.0001; η^2^_G_ = 0.087. Pairwise comparisons for each dimension revealed significant differences (Bonferroni-adjusted) between the psilocybin and the placebo condition with P < 0.01 for all main and sub-dimensions (Fig. 3a). Correlation analysis between the global intensity score and MADRS depressive symptomatology change scores in the psilocybin condition resulted in r(24) = −0.38; P = 0.059.

Furthermore, no significant differences were obtained when comparing the global intensity score between treatment responders and non-responders at visit 7 (+14d) in the psilocybin condition t(17.0) = −1.21; CI −22.7 to 6.1; P = 0.24 or the placebo condition t(3.05) = −1.52; CI −44.5 to 15.5; P = 0.22 (Fig. 3b). For results including all ASC dimensions, see Supplemental Fig. S3.

## Discussion

The present randomised trial comparing a single moderate dose of psilocybin with placebo with equal amounts of psychological support in patients with MDD yielded significant between-group differences at all time points post-treatment over two weeks. The mean between-group differences two weeks post-treatment were −13.0 points on the MADRS and −10.5 points on the BDI. Both the clinician-rated and the self-reported depressive symptom severity scales favoured psilocybin over placebo: at 14 days follow-up, 58% (MADRS) and 54% (BDI) of participants in the psilocybin condition met the criteria for treatment response compared to 16% (MADRS) and 12% (BDI) of participants in the placebo condition. All effects reported from pairwise comparisons remained significant even when applying the Bonferroni method to account for multiple comparisons. The present results support the hypothesis that a single, moderate dose of psilocybin produces clinically significant antidepressant effects in MDD patients compared to a placebo condition controlling for adjunctive psychological support.

The rapid and enduring antidepressant effects of psilocybin-assisted therapy in this study are comparable to those reported in previous trials using multiple and higher doses of psilocybin in patients with MDD, highlighting that a) multiple administrations within a short period, b) high doses, and c) two instead of one therapist accompanying the intervention may not provide additional benefit in the first two weeks after treatment.^6^^,^^8^ Reported effect sizes for efficacy were generally lower in the most recently published trial.^9^ In fact, it is not uncommon to observe decreased efficacy estimates with increasing sample sizes which calls for further investigation of the influence of clinical characteristics (e.g., chronification of disease) and therapy design (e.g., optimal dose and dosing regimen) on treatment response. Furthermore, in the present study, psilocybin-assisted therapy appears to produce a similar rapid onset of antidepressant action as ketamine when comparing response rates obtained 48 h after treatment (69%) with those reported for ketamine obtained at 24 h post-treatment (71%).^17^ However, after 1 week, ketamine's response rates have been found to drop to approximately 35%, suggesting therapeutic advantages of a single, moderate dose psilocybin over ketamine.^17^^,^^18^

Symptom improvements on secondary outcomes obtained during the 2-week post-treatment course were generally more pronounced and clinically significant in the psilocybin as compared to the placebo condition. At visit 7 (+14d), the CGI scale classified the placebo group as moderately ill and the psilocybin group as borderline ill. The global severity index of the SCL-90-R closely follows the CGI trajectory suggesting acceptable convergent validity of the global psychopathology markers used in the present study. Consistent with previous findings in MDD and terminal cancer patients with end-of-life distress, the present trial also showed that psilocybin-assisted therapy significantly reduced anxiety on the SCL-90-R subscale as well as on HAM-A scale at visit 7 (+14d).^6^^,^^19^ Aligning with this, recent studies demonstrated that psilocybin-assisted therapy could bias negative emotion processing to the positive through modulation of limbic structures and associated neural networks.^11^ Hence, the present findings add further evidence to the hypothesis that psilocybin-assisted therapy may have transdiagnostic anxiolytic properties.^20^ However, further studies across different psychiatric conditions are warranted to confirm this assumption.

At the dose tested, psilocybin-assisted therapy produced only moderate and transient cardiostimulant effects, as reported previously in dose–response studies using equivalent doses.^21^^,^^22^ In fact, psilocybin increased systolic and diastolic blood pressure over 5 h, but the heart rate and the rate pressure product as a measure of cardiovascular challenge did not differ statistically from placebo. Hence, despite the possibility of a short-lasting moderate elevation of blood pressure, in participants without pre-existing medical conditions, no cardiovascular complications must be expected from administration of psilocybin in moderate dosages. Moreover, in the present trial, psilocybin was found to have a mild adverse event profile. Of a total of eight adverse events, the most frequently reported was mild headache (11%) which resolved completely within two days after drug administration. The incidence of headache in this study was substantially lower than the rate for mild to moderate headache (24–60%) reported in other clinical trials in MDD using higher doses of psilocybin.^4^^,^^6^^,^^8^^,^^9^ To what extent the incidence of headache depends on dose and/or other psychological and physiological factors needs further investigation. In the present study, suicidal ideation, including potential emergence of suicidal behaviour was assessed by a trained clinician at each study visit. No cases of suicidal behaviour occurred during the trial period of approximately one month. Moreover, at 2-week post-treatment, the scores for suicidal ideation in the psilocybin group corresponded to no suicidal ideation in 24/26 participants, while in the placebo condition, 7/26 reported scores ≥1. The present findings suggest that a moderate dose of psilocybin in a clinical setting with psychological support may not increase the risk for suicidal behaviours in pre-screened patients not displaying acute signs of intention or plan regarding suicidal ideation.

As shown in Fig. 3, most of the participants in the psilocybin group experienced substantial treatment-induced subjective effects, while most participants in the placebo group reported only minor subjective effects. The intensity of the psilocybin-induced subjective effects assessed by the ASC global score (for subscales, see Supplemental Fig. S3) did not correlate with the reduction in depressive symptomatology at 2-week post-treatment. Hence, the present results do not corroborate the assumption that the degree of the drug-induced subjective effects predicts the beneficial outcome in MDD.^23^ Noteworthy, four participants in the placebo group also responded to the treatment. Two of them displayed marked subjective effects on the ASC scale (global > 30%) while the two other placebo responders showed minor alterations (global < 10%). To what extent additional non-pharmacological factors (i.e., psychological aspects) have shaped subjective drug effects and may contribute to the beneficial therapeutic outcome remains to be further investigated. In fact, the necessity of the subjective experience of psychedelics for the enduring therapeutic effects has recently been challenged and is yet debated.^24^^,^^25^ Thus, these present findings must be interpreted with caution. Further mechanistic studies including more specific measures of cognitive and emotional processing may help to clarify this important issue.

The majority of participants enrolled (94%) were Caucasians. Although this reflects the ethnic population distribution in Switzerland, larger trials including more diverse populations are needed to expand the applicability of the findings to races other than white. Another limitation is that the current design did not empirically control for non-pharmacological aspects of the treatment, such as the quality of therapeutic alliance, quality of psychological support during sessions, and expectancy effects regarding trial group assignment. Further research on the impact of non-pharmacological factors is needed to determine better inter-individual differences in responses (Supplemental Fig. S1) and to identify specific therapeutic targets. Furthermore, clinician-rated endpoints were assessed by physicians involved in the study. For further discussion on expectancy effects, see Supplement.

In sum, the results of the present study suggest that a single, moderate dose of psilocybin may be as efficacious in reducing depressive symptoms as the repeated higher doses administered in previous studies while at the same time inducing less adverse events. However, this interpretation needs to be confirmed in future dose–response studies also including long-term follow-up assessments. The intervention was well-tolerated by all participants. Secondary endpoints suggest broad improvements across a variety of psychiatric symptoms. Treatment response was not associated with the intensity of subjective drug effects highlighting the need for further investigations into the psychological and neuronal mechanisms of action of psychedelic substances. Finally, more research into the dose-dependent mechanisms of action of psilocybin is needed to optimise the psychological treatment model embedding psilocybin-assisted therapy.

## Contributors

F.X.V., K.H.P., L.J., E.S., R.v.R. contributed to the conceptualisation of the study. F.X.V., K.H.P., R.v.R. conducted the formal analysis. Data collection was carried out by E.M.S., J.J., A.S., N.M.R., K.Z., A.N., P.N., R.v.R. K.H.P. & R.v.R. verified all underlying data and wrote the original draft. All authors reviewed & edited the manuscript, had full access to all data and accept responsibility for the decision to submit for publication.

## Data sharing statement

Primary endpoints and demographic information collected for the study, including de-identified individual participant data and a data dictionary defining each field in the set, will be made available immediately after publication with no end date to researchers who provide a methodologically sound proposal. Proposals should be directed to the corresponding author. Independently, the study protocol and informed consent form will be uploaded to clinicaltrials.gov after publication.

## Declaration of interests

R.v.R. is currently an employee of, and owns stock in Reconnect Labs AG. E.S. is currently President of Swiss Mental Health Care, President Swiss Society of Anxiety and Depression, and President Swiss Academic Psychiatry, and received consulting fees from Lundbeck Switzerland, Janssen Switzerland, Recordati Switzerland, Takeda Switzerland, Schwabe Switzerland&Germany, and Otsuka Switzerland, and received honoraria for lectures from Lundbeck Switzerland, Janssen Switzerland, Takeda Switzerland, Schwabe Switzerland&Germany, and Otsuka Switzerland, and received support for attending meetings from Lundbeck Switzerland, Janssen Switzerland, and Schwabe Switzerland, and owns stock in Abcellera. L.J. received royalties from published books, received payment as keynote speaker from a speakers bureau (www.speakers.ch), and from a university different from the UZH for lectures. J.J. currently owns stock in Compass Pathways PLC. K.H.P. is currently an employee of Boehringer Ingelheim GmbH & Co KG, and received honoraria for lectures from Johns Hopkins Medical School, USA, and California Institute of Integral Studies, USA, and is currently Chief Scientist for the Heffter Research Institute, and scientific advisor for the MIND foundation. All other contributors to this manuscript declare no conflicting interests.

## Appendix A Supplementary data

Supplemental Tables and Figures

Study Protocol

Summary German
