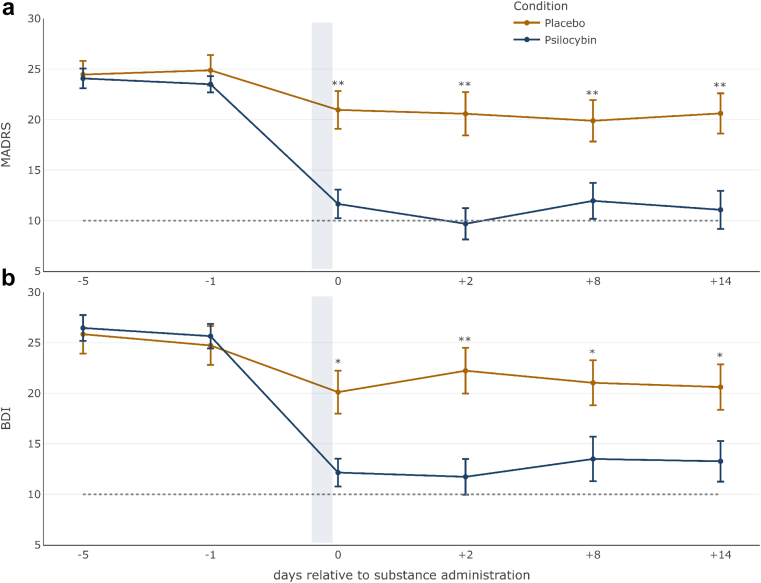# Corrigendum to ‘Single-dose psilocybin-assisted therapy in major depressive disorder: a placebo-controlled, double-blind, randomised clinical trial’

**DOI:** 10.1016/j.eclinm.2023.101841

**Published:** 2023-01-30

**Authors:** Robin von Rotz, Eva M. Schindowski, Johannes Jungwirth, Anna Schuldt, Nathalie M. Rieser, Katharina Zahoranszky, Erich Seifritz, Albina Nowak, Peter Nowak, Lutz Jäncke, Katrin H. Preller, Franz X. Vollenweider

**Affiliations:** a Neurophenomenology of Consciousness Lab, Department of Psychiatry, Psychotherapy and Psychosomatics, Psychiatric Hospital, University of Zurich, Zürich, Switzerland; b Department of Psychiatry, Psychotherapy and Psychosomatics, Psychiatric Hospital, University of Zurich, Zürich, Switzerland; c Division Neuropsychology, Department of Psychology, University of Zürich, Zürich, Switzerland

We wish to provide a correction of the colour representation of Fig. 2 in order to harmonise the colours representing the Psilocybin and the Placebo condition with the corresponding figure caption and with the colour codes from all other figures across the manuscript. Fig. 2 now depicts the Psilocybin condition in blue and the Placebo condition in yellow. The error occurred as a result of an oversight. This change does not affect the results presented in the paper and the figure legend remains unchanged.

**Figure:**