# Supplementary material for: Single-dose psilocybin-assisted therapy in major depressive disorder: A placebo-controlled, double-blind, randomised clinical trial
Source: eClinicalMedicine. 2022 Dec 28;56:101809. doi: 10.1016/j.eclinm.2022.101809 (PMC9830149; doi:10.1016/j.eclinm.2022.101809)
Supplement: Supplemental Tables and Figures [file mmc1.pdf]

Supplementary Materials

Recruitment

Participants were recruited through advertisements on the webpage of the Psychiatric University Hospital Zurich in conjunction with distribution of flyers and public advertisements in local and national newspapers. Additionally, several ambulatory clinics and private practitioners in Switzerland were invited to refer participants with an acute current depressive episode to the study.

Assessment of demographic information

All demographic variables (summarised in **Table 1**) were assessed at the medical screening visit (visit 1) and consisted of information on age, gender, ethnicity, verbal IQ, and depressive symptoms severity, as well as concomitant medication before trial enrolment, and history of prior experiences with psychedelic agents. The Mehrfachwahl-Wortschatz-Intelligenztest (MWT-B), a commonly applied instrument to infer verbal intelligence, was used to derive individual verbal IQ scores. Sum scores refer to the amount of correctly identified German words with a maximum of 37 points. Sum scores were subsequently compared to the appropriate norm table in order to estimate verbal intelligence.[1] Greater scores indicate higher verbal IQ. Concomitant medication was assessed by the screening physician in a structured interview and subsequently categorised into antidepressant medication and prescription medication. Prescription medication includes all pharmaceuticals only available on prescription, excluding antidepressant medication. Notably, the number of antidepressant medications refers to the time point before discontinuation as required by the inclusion criteria. The number of prior experiences with psychedelic substances was derived using a structured interview. Every substance primarily acting as a 5-HT-2A receptor agonist was included into the score.

Additional study procedures

The telephone pre-screening was carried out by specifically trained study team members. It consisted of a structured interview to collect information on demographics, symptom severity, and somatic health. Furthermore, the potential participant was provided general information on the trial. 94% of all pre-screened participants were either determined not eligible or declined participation after the pre-screening (**Figure 1**). Pre-screened participants were invited for an in-person medical screening visit (visit 1) to determine eligibility.

One specifically trained therapist was responsible for all visits of a specific participant to establish consistency across study visits and build trust in the therapeutic relationship. Study related procedures such as administering questionnaires were completed by a research assistant.

In addition to the outcomes reported in this manuscript, participants underwent three one-hour sessions of functional magnetic resonance imaging, four neuropsychological assessments. Three blood samples were taken over the course of the study. Furthermore, participants completed clinical and psychological follow-up assessments. These data will be published at a later date.

Sample size power calculation

Previous clinical studies investigating rapid antidepressant efficacy of ketamine in depressed patients in randomised placebo-controlled trials showed effect sizes of Cohens' d=0·68-1·46 for MADRS scores.[2,3] With the conservative assumption of f=0·5, an alpha-probability of 0·05 (two-sided) and a power of 0·8 (mixed-effects model of variance), we would need at least 22 individuals per treatment arm to detect significant differences. We therefore suggested a sample size of N=30 individuals per treatment arm given the lack of previous efficacy studies of psilocybin in depression at the time. Due to operational reasons the study was terminated after having reached 26 participants per treatment condition in accordance with the power analysis.

Calculations of psychometric scales

MADRS: Construct validity of the German version of the MADRS is favourable compared to similar instruments (e.g., Hamilton Depression Rating Scale) while maintaining acceptable inter-rater reliability rates.[4] The items from this structured clinical interview range from zero to six on a 7-point Likert-scale and a sum score is used for the calculation of MDD severity, with a maximum total score of 60 points. Higher scores indicate greater depression severity.

BDI: The questionnaire has excellent psychometric properties and consists of 21 items ranging from zero to three. Self-reported scores are summed up across all items with a maximum of 63 points. Higher scores indicate greater depression severity.[5,6]

ASC: The ASC questionnaire is a well-established instrument to quantify subjective alterations of the state of consciousness by using 94 visual analogue items ranging from 0-100. The ASC questionnaire assesses five main dimensions of ASC termed oceanic (self) boundlessness; dreadful ego dissolution, visual restructuralization, auditory alterations, and vigilance reduction, which can be further described by 11 second order dimensions termed: experience of unity; spiritual experiences; blissfulness; insightfulness; disembodiment; impaired cognition and control; anxiety; elementary imagery; complex imagery; audio-visual synesthesia; and changed meaning of percepts.[7] Greater scores indicate greater distortion of everyday consciousness.

SCL-90-R: The 90 items included in the instrument range from zero to four on a 5-point Likert scale and can be aggregated into nine distinct scales (somatization, obsessive-compulsive, interpersonal sensitivity, anger-hostility, anxiety, depression, psychoticism, paranoid ideation, and phobic anxiety). Additionally, a global severity index is calculated providing an aggregated overview of all covered symptoms by summing up all items and dividing the sum by the number of items with a possible maximum score of four points. Greater scores indicate greater severity of symptoms.

CGI: The CGI consists of two items measuring clinical impressions of overall severity of psychopathology and improvements from baseline. Improvement was not reported in the manuscript in order to reduce redundancy. The severity item is rated on a scale ranging from two to nine (1 = not assessable) by an experienced clinician at each study visit to estimate global severity and of clinical impressions. Greater scores indicate greater severity of psychopathology.

HAM-A: The total score represents the sum of all 14 item scores assessed on a 5-point Likert-scale ranging from zero to four. Greater scores indicate greater severity of symptoms.

C-SSRS: At the medical screening, the life-time version of the instrument was administered to gain additional insights into latent dispositions for suicidality. For all other study visits the follow-up version was used, referring to the timeframe since the last visit. The main characteristic derived from the scale evaluates the intensity of acute suicidal ideation. The underlying conceptual definitions of this questionnaire are based on the Columbia Suicide History Form.[8] The intensity of acute suicidal ideation is assessed on a scale ranging from zero to a maximum of five. A score of zero indicates no acute suicidal ideation and a score of five points at the presence of active suicidal ideation with specific plan and intent.

Statistical analysis: Model assumptions

The following assumptions were tested prior to each ANOVA being calculated: a) Shapiro-Test for testing the empirical distribution of the present data to be normally distributed at each timepoint across conditions; b) Levene's test for the estimation of homogeneity of variances at each timepoint across conditions; c) Mauchly's test for uncovering violations of the sphericity assumption.

Statistical analysis: Secondary endpoints

To investigate effects of treatment condition between all study visits for secondary endpoints, mixed models analysis of variance were applied to investigate the interaction between treatment condition and study visit for CGI, HAM-A, and C-SSRS. Because the SCL-90-R was only assessed at visits 2 and 7, missing values were not imputed and participants with missing values were removed from the population analysed. Mixed-effects models analysis of covariance (ANCOVA) were used analogously to the primary endpoint analysis. Post-hoc analysis consisted of two-sample Welch's t-tests comparing treatment conditions at visit 7. Cohens' *d* was used to estimate effect sizes. All statistical tests used $P<0{\cdot}05$, two-tailed to determine statistical significance.

Statistical analysis and data management software

Mixed effects models of variance and subsequent post-hoc analyses were calculated using the rstatix r-package.[9] Figures were produced using the r-packages plotly, ggpubr, and ggplot2.[10-12] Study data were collected and managed using REDCap electronic data capture tools.[13,14]

Convergent Validity

Convergent validity between self-reported (BDI) and clinician-rated (MADRS) instruments was calculated using Pearson's correlation coefficients for each timepoint and group. Assessment of convergent validity between self-reported and clinician-rated MDD severity as measured by BDI and MADRS, resulted in a correlation coefficient of r=0·82; P<0·0001.

Primary endpoints: Individual trajectories

In addition to analysing mean trajectories, we focused on inter-individual differences in symptom trajectories over time (**Supplemental Figure 1**). MADRS individual distribution trajectories described in terms of variance σ^2 [range] over time for psilocybin: visit 2 (-5d)=25·6 [13; 29]; visit 4 (0d)=52·0 [1; 31]; visit 7 (+14d)=62·9 [0; 29] and in the placebo condition: visit 2 (-5d)=49·9 [11; 38]; visit 4 (0d)=91·6 [7; 45]; visit 7 (+14d)=106·3 [1; 45]. BDI individual distribution trajectories described in terms of variance σ^2 [range] over time in the psilocybin condition: visit 2 (-5d)=44·8 [11; 39]; visit 4 (0d)=49·8 [0; 30]; visit 7 (+14d)=77·3 [2; 37] and the placebo condition: visit 2 (-5d)=91·9 [10; 44]; visit 4 (0d)=117·0 [2; 45]; visit 7 (+14d)=77·3 [2; 37].

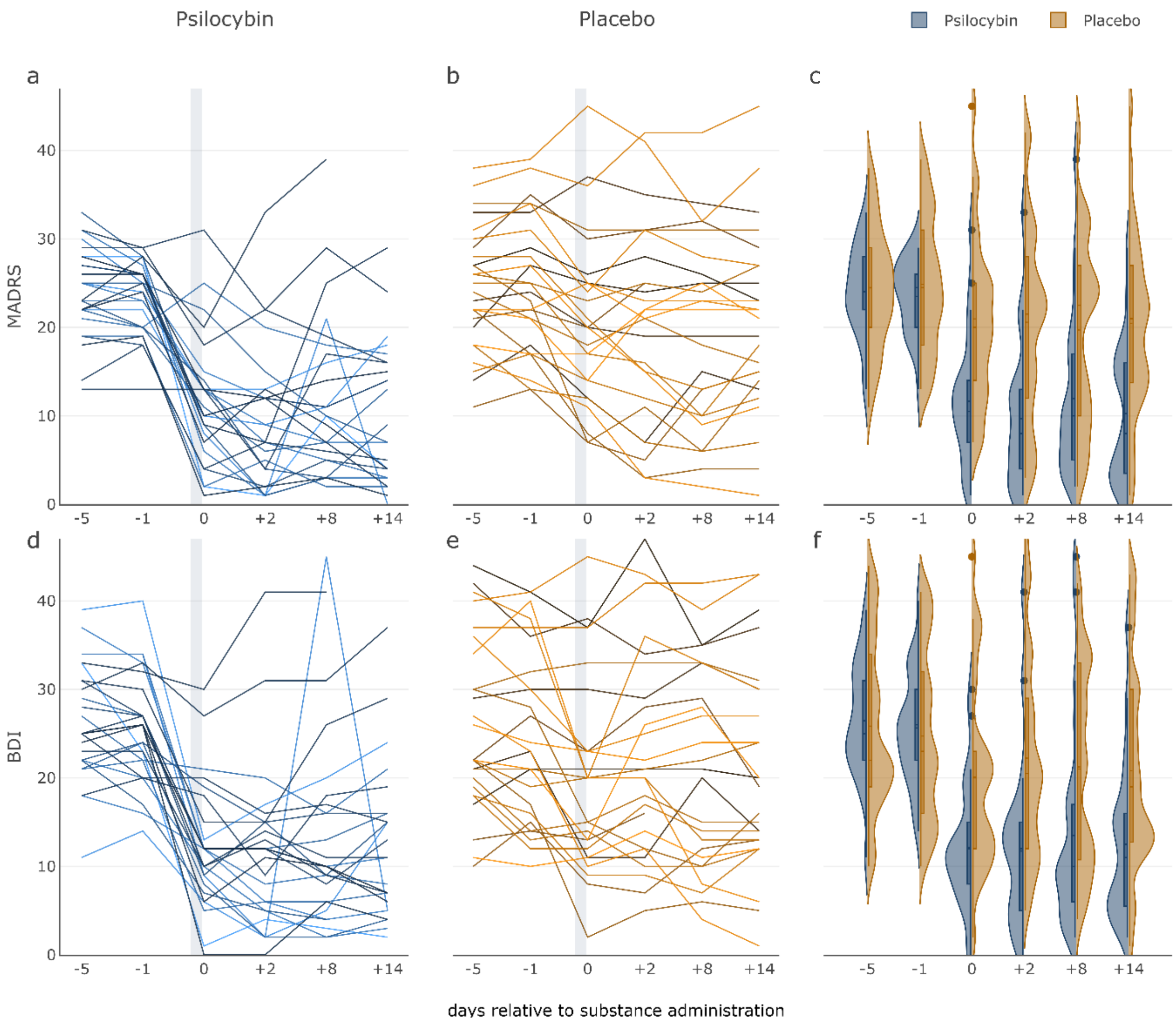

Supplemental Figure 1. Individual trajectories of MADRS and BDI scores at every study visit. **A**: MADRS sum scores individual trajectories of participants in the psilocybin condition; **B**: MADRS sum scores individual trajectories of placebo participants; **C**: Split density plots with embedded boxplots depicting the distribution of MADRS sum scores at each study visit (left: psilocybin; right: placebo); **D**: BDI sum scores individual trajectories of participants in the psilocybin condition; **E**: BDI sum scores individual trajectories of placebo participants. **F**: Split density plots with embedded boxplots depicting the distribution of BDI sum scores at each study visit (left: psilocybin; right: placebo); density estimation in **C** and **F** was obtained with a gaussian density estimator kernel utilising a bandwidth of 2.1 which is slightly below silvermann's rule of thumb to facilitate detection of outliers.[15] Boxplots within density distributions depict median (center line), interquartile range (box) and 1.5* interquartile range (whiskers). Outliers are visualised if distance to median is larger than 1.5* interquartile range. The grey bar depicts the time of substance administration.

Questionnaire	Scale	PLACEBO Baseline mean (SD)	PLACEBO 14 days mean (SD)	PSILOCYBIN Baseline mean (SD)	PSILOCYBIN 14 days mean (SD)	T-TEST mean difference	T-TEST 95% CI	T-TEST P-value	T-TEST Cohens' *d*
**SCL-90-R**	Somatization	0·58 (0·41)	0·55 (0·56)	0·43 (0·36)	0·35 (0·38)	-0·09	-0·07 to 0·47	0·14	0·42
	Obsessive-compulsive	1·26 (0·57)	1·04 (0·76)	1·36 (0·43)	0·79 (0·47)	-0·57	-0·11 to 0·62	0·16	0·40
	Interpersonal sensitivity	1·15 (0·80)	1.05 (0·76)	1·29 (0·75)	0·83 (0·64)	-0·46	-0·18 to 0·62	0·28	0·31
	Anger-hostility	0·65 (0·54)	0·61 (0·60)	0·56 (0·49)	0·38 (0·33)	-0·19	-0·05 to 0·51	0·099	0·48
	Anxiety	0·72 (0·63)	0·72 (0·62)	0·58 (0·34)	0·38 (0·33)	-0·20	0·05 to 0·62	0·024	0·67
	Depression	1·75 (0·77)	1·43 (0·80)	1·67 (0·52)	0·84 (0·62)	-0·83	0·18 to 1·01	0·0056	0·83
	Psychoticism	0·50 (0·40)	0·45 (0·43)	0·54 (0·37)	0·26 (0·27)	-0·30	0·02 to 0·43	0·030	0·64
	Paranoid Ideation	0·57 (0·46)	0·53 (0·54)	0·74 (0·64)	0·35 (0·40)	-0·39	-0·09 to 0·46	0·174	0·39
	Phobic Anxiety	0·45 (0·52)	0·33 (0·31)	0·20 (0·27)	0·11 (0·14)	-0·09	0·08 to 0·36	0·0031	0·91
	Global Severity Index	0·93 (0·44)	0·80 (0·49)	0·87 (0·33)	0·50 (0·33)	-0·33	0·06 to 0·53	0·017	0·71
**HAM-A**	Total score	16·08 (7·28)	12·58 (7·62)	15·35 (5·66)	7·58 (6·03)	-7·77	1·17 to 8·83	0·012	0·73
**CGI**	Severity	5·65 (0·89)	5·19 (1·36)	5·50 (0·58)	3·88 (1·40)	-1·62	0·54 to 2·07	0·0012	0·95
**CSSR-S**	Intensity	0·54 (0·81)	0·46 (0·95)	0·50 (0·76)	0·15 (0·37)	-0·35	-0·10 to 0·71	0·13	0·43

The symptom checklist 90-revised (SCL-90-R) consists of nine subscales and one global index and included N=25 participants per condition; Hamilton-Anxiety Scale (HAM-A) is summarised as a total score; clinical global impressions (CGI) provides an indication-independent severity scale; Colombia-Suicidality severity rating scale (C-SSRS) was used to detect acute suicidality on a low-threshold by deriving intensity of ideation. Each questionnaire was administered at baseline five days before drug administration (except SCL-90-R, which was assessed during medical screening) and two weeks after the trial intervention. Mean differences from baseline, 95% confidence intervals, and P-values were derived using two-samples Welch's t-test. Cohens' *d* estimates effect sizes between treatment conditions at visit 7 (+14d).

Supplemental Table 1. Statistical analysis of secondary endpoints.

Vital signs

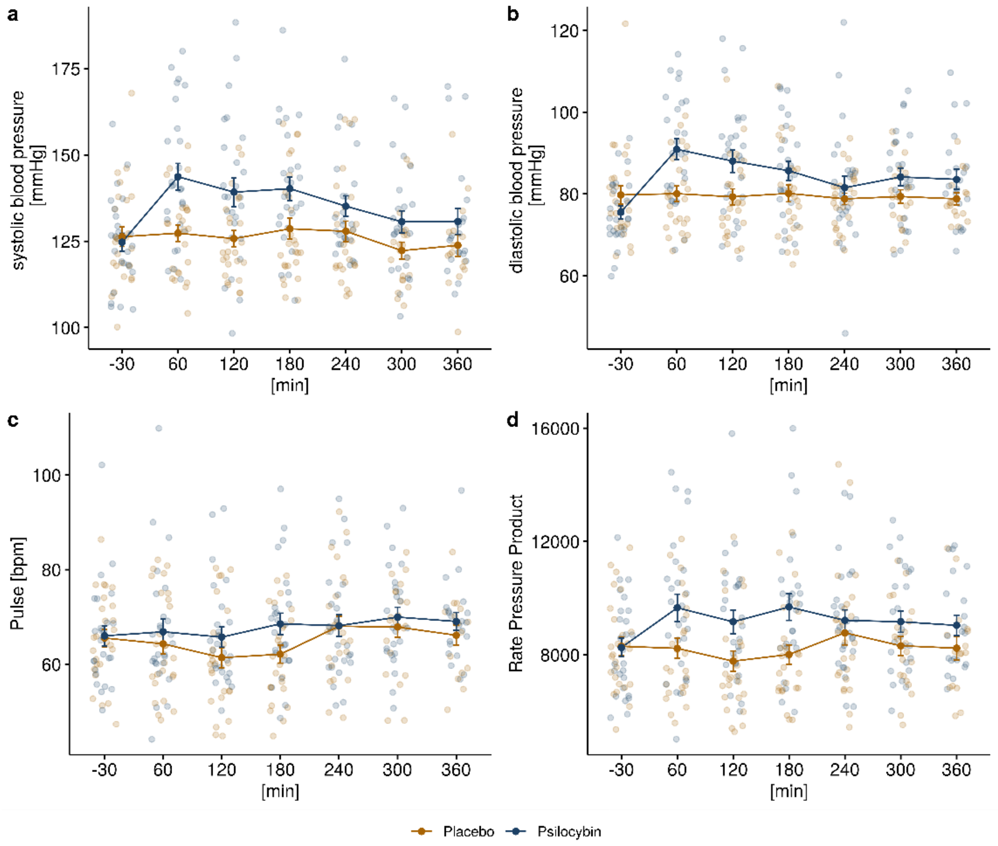

Supplemental Figure 2. Vital signs during administration day. **A**: Dotplots depicting systolic (left) and diastolic (right) blood pressure [mmHg] over the course of acute drug effects; **B**: Mean ± SE of pulse rate over the course of acute drug effects [beats per minute; bpm]. Jittered dots represent individual measurements; **C:** Mean ± SE rate pressure product (RPP) over the course of acute drug effects. Jittered dots represent individual measurements. RPP surrogates drug-induced cardiovascular challenges.

Correlation analysis

Pearson's correlation was applied to test the association between subjective effects induced by psilocybin and symptom change scores from visit 2 (-5d) to visit 7 (+14d). The placebo condition was not included in the analysis to prevent violation of assumptions due to skewed distribution towards zero (see **Figure 3**). Correlations were calculated for all ASC questionnaire dimensions (see **Supplemental Figure 3**).

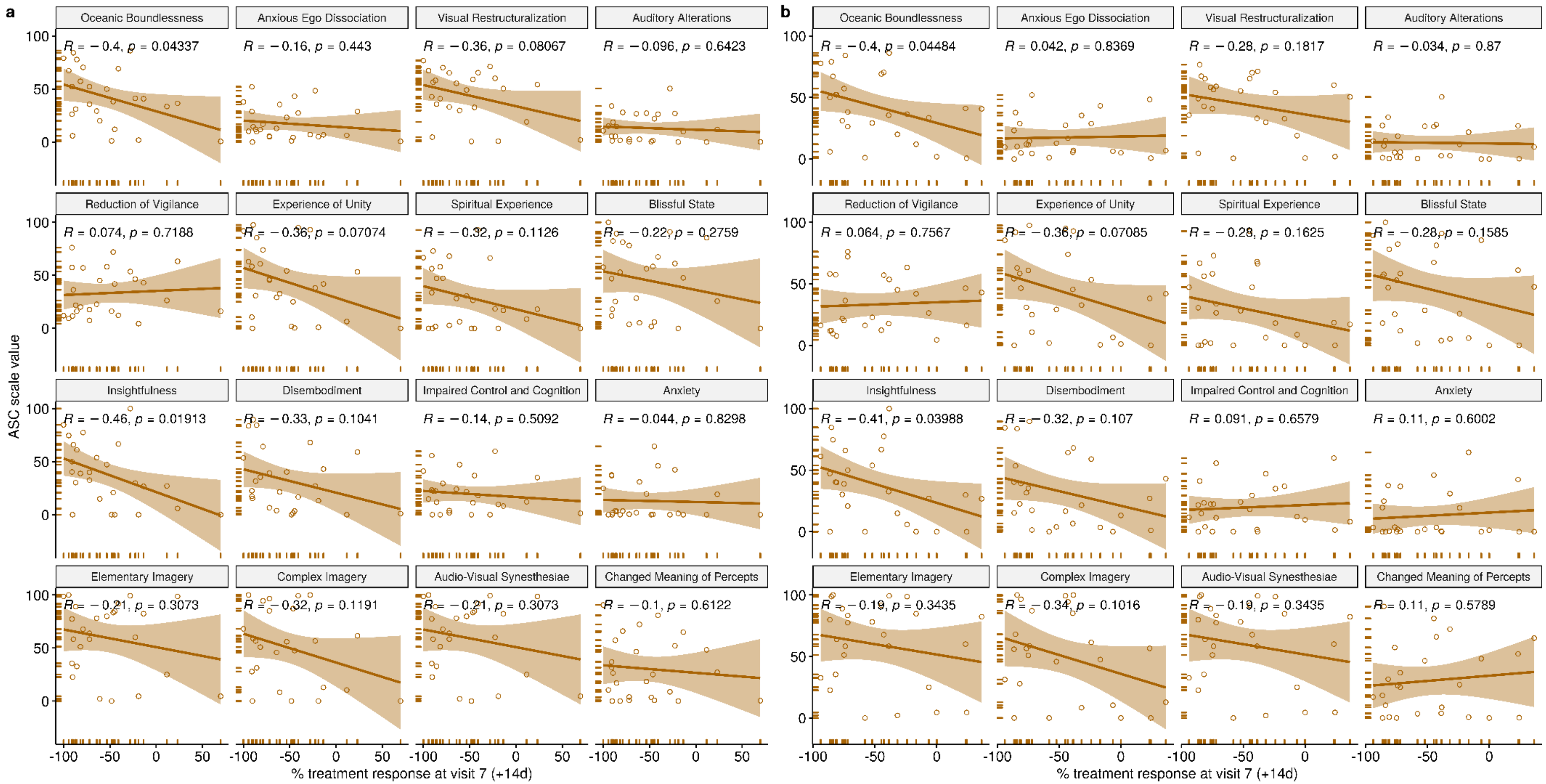

Supplemental Figure 3. Associations of MADRS and BDI change scores with ASC questionnaire dimensions. Significant associations between changes in depressive symptoms (visit 7) and intensity of subjective effects induced by psilocybin were found for oceanic boundlessness and insightfulness. Reported p-values are uncorrected. None of the correlations remained significant after Bonferroni-correcting for multiple comparisons. N=26.

	Psilocybin (N=26)	Placebo (N=26)
**Headache**	4 (15%)	0
resolved in (mean days)	1·75	
**Dizziness**	2 (8%)	0
resolved in (mean days)	1·00	
**Nausea**	1 (4%)	0
resolved in (mean days)	1·00	
**Diarrhea**	1 (4%)	0
resolved in (mean days)	4·00	
**Common Cold**	0	2 (8%)
resolved in (mean days)		2·50
**Cystitis**	0	1 (4%)
resolved in (mean days)		3·00

Adverse events reported from a total of N=52 participants over the course of the study, excluding acute, transient symptoms directly related to the well-known psychotropic effects of psilocybin. Causal relatedness to the active pharmaceutical compound administered and duration to completely resolve were rated for each adverse event.

Supplemental Table 2. Overview of adverse events reported.

Expectancy

The content of and time spent to provide psychological support including appropriate preparation and integration of the experience during administration day is often insufficiently reported in trials investigating efficacy in experience-based therapies.[16] The amount of time spent with a clinician is likely to correlate with treatment response.[17] Therefore, it was expected that both conditions in the present study would display increasing symptom alleviation over time. However, the placebo group did not respond to an a priori expected extent, and symptom severity did not significantly differ from baseline scores. This points towards potential blinding issues regarding the drug conditions. The growing interest towards psychedelic therapy expands beyond scientific inquiry. During the last decade online searches about psychedelic therapy have quadrupled.[18] This spotlight can trigger major expectations of participants, which in turn might bear the potential to influence the outcome of clinical trials.[19] In this study, we observe both improvement and worsening of depressive symptoms in the placebo group (**Supplemental Figure 1**). This may be due to unblinding during or after the administration visit. If a participant

is convinced to be part of the control group, disappointment is inevitable, and in turn a nocebo effect may be observed, leading to MDD symptomatology aggravation.

Protocol discrepancies

The authors became aware of discrepancies within the protocol (sections 15.4.1. and 15.4.2.) after locking the database, whereas in the first section the study population was defined according to the intention-to-treat (ITT) principle, while the latter section only included patients that completed all study visits into the analysis. The ITT principles are considered best practice for the selection of the efficacy population and were therefore applied in the analysis reported in the present manuscript. In addition, the primary endpoint definition differed between the protocol (MARDS only) and the clinical trial registry (clinicaltrials.gov, MARDS & BDI). Analysing two primary endpoints was chosen because it is more conservative.The statistical analysis was adapted after expert review of this paper and therefore differs from the statistical methods described in the protocol. Both methods reveal the same results.

Supplemental references

1 Lehrl S. Mehrfachwahl-Wortschatz-Intelligenztest: MWT-B. Spitta, 1999.

2 Zarate CA Jr, Singh JB, Carlson PJ, *et al.* A Randomized Trial of an N-methyl-D-aspartate Antagonist in Treatment-Resistant Major Depression. *Arch Gen Psychiatry* 2006; **63**: 856–64.

3 aan het Rot M, Collins KA, Murrough JW, *et al.* Safety and Efficacy of Repeated-Dose Intravenous Ketamine for Treatment-Resistant Depression. *Biol Psychiatry* 2010; **67**: 139–45.

4 Schmidtke A, Fleckenstein P, Moises W, Beckmann H. Studies of the reliability and validity of the German version of the Montgomery-Asberg Depression Rating Scale (MADRS). *Schweiz Arch Neurol Psychiatr Zurich Switz 1985* 1988; **139**: 51–65.

5 Hautzinger M. 26 Verhaltenstherapie bei unipolaren und bipolaren affektiven Störungen. *Verhal Grundlagen-Methoden-Anwendungsgebiete 59 Tabellen* 2006.

6 Schwab J, Bialow M, Clemmons R, Martin P, Holzer C. The Beck Depression Inventory with Medical Inpatients. *Acta Psychiatr Scand* 1967; **43**: 255–66.

7 Studerus E, Gamma A, Vollenweider FX. Psychometric Evaluation of the Altered States of Consciousness Rating Scale (OAV). *PLoS ONE* 2010; **5**: e12412.

8 Oquendo MA, Halberstam B, Mann JJ. Risk factors for suicidal behavior. *Stand Eval Clin Pract* 2003; **22**: 103–29.

9 Kassambara A. rstatix: Pipe-Friendly Framework for Basic Statistical Tests. 2021 https://rpkgs.datanovia.com/rstatix/.

10 Kassambara A. ggpubr: ‘ggplot2’ Based Publication Ready Plots. 2020 https://rpkgs.datanovia.com/ggpubr/.

11 Sievert C. Interactive Web-Based Data Visualization with R, plotly, and shiny. , 2020. Florida: Chapman and Hall/CRC, 2020 https://plotly-r.com.

12 Wickham H. ggplot2: Elegant Graphics for Data Analysis. New York: Springer-Verlag New York, 2016 https://ggplot2.tidyverse.org.

13 Harris PA, Taylor R, Thielke R, Payne J, Gonzalez N, Conde JG. Research electronic data capture (REDCap)—A metadata-driven methodology and workflow process for providing translational research informatics support. *J Biomed Inform* 2009; **42**: 377–81.

14 Harris PA, Taylor R, Minor BL, *et al.* The REDCap consortium: Building an international community of software platform partners. *J Biomed Inform* 2019; **95**: 103208.

15 Silverman BW. Density Estimation for Statistics and Data Analysis, Chapman and Hall, London, 1986. *Crossref Á* 1986.

16 Muthukumaraswamy SD, Forsyth A, Lumley T. Blinding and expectancy confounds in psychedelic randomized controlled trials. *Expert Rev Clin Pharmacol* 2021; **14**: 1133–52.

17 Grawe K. Psychological therapy. Seattle, WA: Hogrefe & Huber Publishers, 2002.

18 Google Trends. Google Trends. https://trends.google.com/trends/explore?date=all&q=psychedelic%20therapy (accessed July 14, 2022).

19 Kaertner LS, Steinborn MB, Kettner H, *et al.* Positive expectations predict improved mental-health outcomes linked to psychedelic microdosing. *Sci Rep* 2021; **11**: 1941.
