# Supplementary material for: Single-dose psilocybin-assisted therapy in major depressive disorder: A placebo-controlled, double-blind, randomised clinical trial
Source: eClinicalMedicine. 2022 Dec 28;56:101809. doi: 10.1016/j.eclinm.2022.101809 (PMC9830149; doi:10.1016/j.eclinm.2022.101809)
Supplement: Study Protocol [file mmc2.pdf]

Clinical Study Protocol

PHASE II, RANDOMIZED, DOUBLE BLIND, PLACEBO CONTROLLED, PARALLEL GROUP, SINGLE CENTER STUDY OF PSILOCYBIN EFFICACY IN MAJOR DEPRESSION

"Clinical, Neurocognitive, and Emotional Effects of Psilocybin in Depressed Patients - Proof of Concept"

**Study Type:**	Intervention with Investigational Medicinal Product (IMP)
**Study Categorisation:**	Clinical Trial with IMP Category C
**Study Registration:**	ClinicalTrials.gov (intended registry)
**Study Identifier:**	PSIDEPR-128 (institutional identifier)

Sponsor-Investigator and Principal Investigator:

Prof. Dr. med. Franz X. Vollenweider
Department of Psychiatry, Psychotherapy and Psychosomatics
Neuropsychopharmacology and Brain Imaging
Psychiatric Hospital, University of Zurich
Lenggstrasse 31
CH-8032 Zürich,
Switzerland
E-mail: vollen@bli.uzh.ch
Phone: +41 44 384 24 04

Co-Investigator:

Dr. phil. Katrin Preller
Department of Psychiatry, Psychotherapy and Psychosomatics
Neuropsychopharmacology and Brain Imaging
Psychiatric Hospital, University of Zurich
Lenggstrasse 31
CH-8032 Zürich,
Switzerland
E-mail: preller@bli.uzh.ch
Phone: +41 44 384 23 12

**Investigational Product:**	Psilocybin
**Protocol Version and Date:**	Version 7, 30.09.2020

CONFIDENTIAL

The information contained in this document is confidential and the property of the Psychiatric University Hospital Zürich. The information may not - in full or in part - be transmitted, reproduced, published, or disclosed to others than the applicable Independent Ethics Committee(s) and Competent Authority(ies) without prior written authorization from the Psychiatric University Hospital Zürich, except to the extent necessary to obtain informed consent from those participants who will participate in the study.

1 SIGNATURE PAGES

**Study number**	ClinicalTrials.gov (NCT03715127)
**Study Title**	”Phase II, randomized, double blind, placebo controlled, parallel group, single center study of psilocybin efficacy in major depression”

Sponsor-Investigator/ Principal Investigator:

The Sponsor-Investigator and trial statistician have approved the protocol version (7 dated 30.09.2020), and confirm hereby to conduct the study according to the protocol, current version of the World Medical Association Declaration of Helsinki, ICH-GCP guidelines and the local legally applicable requirements.

Prof. Dr. med. Franz X. Vollenweider

Zurich, 30.09.2020

Place/Date	Signature

Local Principal Investigator at study site:

I have read and understood this trial protocol and agree to conduct the trial as set out in this study protocol, the current version of the World Medical Association Declaration of Helsinki, ICH-GCP guidelines and the local legally applicable requirements.

**Site**	Department of Psychiatry, Psychotherapy and Psychosomatics<br>Neuropsychopharmacology and Brain Imaging<br>Psychiatric Hospital, University of Zurich<br>Lenggstrasse 31<br>CH-8032 Zürich,<br>Switzerland
**Principal investigator**	Prof. Dr. med. Franz X. Vollenweider

Zurich, 30.09.2020

Place/Date	Signature

TABLE OF CONTENTS

1 SIGNATURE PAGES ... 2
2 STUDY SYNOPSIS ... 8
3 ABBREVIATIONS ... 12
4 STUDY SCHEDULE ... 13
5 STUDY ADMINISTRATIVE STRUCTURE ... 17
5.1 Sponsor-Investigator and Principal Investigator ... 17
5.2 Co-Investigators ... 18
5.3 Statistician ("Biostatistician") ... 18
5.4 Laboratory ... 19
5.4.1 On-Site Laboratory ... 19
5.4.2 Collaboratoring Laboratory ... 19
5.5 Monitoring Institution and Data Safety Monitoring Committee ... 20
5.6 Any other relevant Committee, Person, Organisation, Institution ... 20
6 ETHICAL AND REGULATORY ASPECTS ... 20
6.1 Study Registration ... 20
6.1.1 Birth date ... 21
6.2 Categorisation of Study ... 21
6.3 Competent Ethics Committee (CEC) ... 21
6.4 Competent authority (CA) ... 21
6.5 Ethical Conduct of the Study ... 21
6.6 Declaration of Interest ... 21
6.7 Patient Information and Informed Consent ... 22
6.8 Participant Privacy and Confidentiality ... 22
6.9 Early Termination of the Study ... 23
6.10 Protocol Amendments ... 23
7 INTRODUCTION ... 23
7.1 Background and Rationale ... 32
7.2 Investigational Product and Indication ... 32
7.3 Preclinical Evidence ... 33
7.4 Clinical Evidence to Date ... 34
7.5 Dose Rationale ... 34
7.6 Explanation for Choice of Comparator (or Placebo) ... 35
7.7 Risk / Benefits ... 35
7.7.1 Potential Risks ... 35
7.7.2 Mitigation of risks ... 38
7.7.3 Potential Benefits ... 39
7.8 Justification of Choice of Study Population ... 39
8 STUDY OBJECTIVES ... 40
8.1 Overall Objective ... 40
8.2 Primary Objective ... 40
8.3 Secondary Objectives ... 41
8.4 Safety Objectives ... 41

9 STUDY OUTCOMES 41
9.1 Primary Outcome 41
9.2 Secondary Outcomes....................42
9.3 Safety Outcomes....................44
10 STUDY DESIGN AND COURSE OF STUDY 45
10.1 General Study Design and Justification of the Design 45
10.2 Methods of Minimising Bias....................45
10.2.1 Randomisation....................45
10.2.2 Blinding Procedures....................46
10.2.3 Other Methods of Minimising Bias....................46
10.3 Unblinding Procedures (Code break) 46
11 STUDY POPULATION 46
11.1 Eligibility Criteria....................46
11.1.1 Inclusion Criteria 46
11.1.2 Exclusion Criteria....................47
11.2 Recruitment, Screening, and Payment 48
11.3 Assignment to Study Groups....................49
11.4 Criteria for Withdrawal/ Discontinuation of Participants 49
12 STUDY INTERVENTION....................49
12.1 Identity of Investigational Product(s)....................49
12.1.1 Experimental Intervention 50
12.1.2 Control Intervention 50
12.1.3 Packaging, Labelling and Supply (Re-Supply) 51
12.1.4 Storage Conditions 52
12.2 Administration of Experimental and Control interventions....................52
12.2.1 Experimental Intervention 52
12.2.2 Control Intervention 52
12.3 Dose Modifications....................53
12.4 Compliance with Study Intervention 53
12.5 Data Collection and Follow-up for Withdrawn Participants 53
12.6 Trial Specific Preventive Measures 53
12.7 Concomitant Intervention(s) 54
12.8 Study Drug Accountability 55
12.9 Return or Destruction of Study Drug....................55
13 STUDY ASSESSMENTS 56
13.1 Study Flow Chart/ Table of Study Procedures and Assessments 56
13.2 Assessments of Outcomes....................60
13.2.1 Assessment of Primary Outcome....................60
13.2.2 Assessment of Secondary Outcomes 60
13.2.3 Assessment of Other Outcomes of Interest....................61
13.2.4 Assessment of Safety Outcomes 61
13.2.5 Assessments in Participants Who Prematurely Stop the Study....................62
13.3 Procedures at Each Visit 62
13.3.1 Pre-Screening (Phone Call) 62

13.3.2 Visit 1 or Screening Visit 62
13.3.3 Visit 2 or Pre-investigation Visit 62
13.3.4 Visit 3 or Baseline Measures Visit 63
13.3.5 Visit 4 or Treatment Session Visit 63
13.3.6 Visit 5 or Post-Treatment Measures Visit 63
13.3.7 Visit 6 or Follow-up Visit 64
13.3.8 Visit 7 or Follow-up Measures and Closure Visit 64
14 SAFETY 65
14.1 Definition of (Serious) Adverse Events and Other Safety Related Events 65
14.2 Recording of (Serious) Adverse Events and Other Safety Related Events 67
14.3 Assessment of (Serious) Adverse Events and Other Safety Related Events 67
14.4 Reporting of Serious Adverse Events and Other Safety Related Events 68
14.5 Follow up of (Serious) Adverse Events 69
15 STATISTICAL METHODS 70
15.1 Hypothesis 70
15.2 Determination of Sample Size 70
15.3 Statistical Criteria of Termination of Trial 71
15.3.1 Criteria of stopping for benefit 71
15.3.2 Criteria of stopping for futility 71
15.3.3 Criteria of stopping for harm 71
15.4 Planned Analyses 71
15.4.1 Datasets to be Analysed, Analysis Populations 71
15.4.2 Primary Analysis 71
15.4.3 Secondary Analyses 71
15.4.4 Interim Analyses 72
15.4.5 Safety Analysis 72
15.4.6 Deviation(s) from the Original Statistical Plan 72
15.5 Handling of Missing Data and Drop-Outs 72
16 DATA QUALITY ASSURANCE AND CONTROL 72
16.1 Data Handling and Record Keeping / Archiving 73
16.1.1 Case Report Forms 73
16.1.2 Specification of Source Documents 73
16.1.3 Record Keeping / Archiving 74
16.2 Data Management 74
16.2.1 Data Management System 74
16.2.2 Data Security, Access and Back-up 74
16.2.3 Analysis and Archiving 75
16.2.4 Data Validation 75
16.3 Monitoring 75
16.4 Audits and Inspections 75
16.5 Confidentiality, Data Protection 75
16.6 Storage of Biological Material and Related Health Data 76
17 PUBLICATION AND DISSEMINATION POLICY 76
18 FUNDING AND SUPPORT 76

19 INSURANCE 76
20 REFERENCES 77

2 STUDY SYNOPSIS

**Sponsor / Sponsor-Investigator**	Prof. Dr. med. Franz X. Vollenweider
**Study Title:**	Phase II, randomized, double blind, placebo controlled, parallel group, single center study of psilocybin efficacy in major depression
**Short Title / Study ID:**	Clinical, neurocognitive and emotional effects of psilocybin in depressed patients: proof of concept / PSIDEPR-128
**Protocol Version and Date:**	Version 6 of 20.08.2019
**Trial registration:**	ClinicalTrials.gov (NCT03715127)
**Study category and Rationale**	Clinical study with IMP Category C
**Clinical Phase:**	Clinical Phase II
**Background and Rationale:**	Major depression is characterized by specific neurocognitive and emotional impairments. These deficits may underlie its vulnerability, symptom formation and treatment response. We and others have previously shown that psilocybin acts as a serotonin receptor agonist in the brain, and that the effects of psilocybin may improve neurocognitive and emotional functioning in healthy volunteers. Therefore, psilocybin may provide a novel approach to treatment of depression. Given that currently available drugs for the treatment of depression have delayed onset of action, are not effective in all patients, and are often associated with unwanted side effects, we consider this proof-of-concept study as important and promising.
**Objective(s):**	To evaluate effects of psilocybin on clinical symptoms, neurocognitive and emotional measures in major depression.
**Outcome(s):**	Primary outcome measures:<br>• Depression severity on Montgomery Asberg Depression Rating Scale (MADRS)<br>Secondary outcome measures:<br>• Neurocognitive and emotional brain functioning markers for depression using functional Magnetic Resonance Imaging (fMRI)<br>• Standardized cognitive assessment using Multifaceted Empathy Task Battery<br>• Plasma & Serum BDNF<br>• Depression symptom profile measures (e.g. anhedonia, anxiety, rumination)
**Study design:**	Randomized, double blind, placebo controlled, parallel group, single center study
**Key Inclusion / Exclusion criteria:**	Inclusion criteria:<br>• Male or female in- or outpatients at the age of 18-60<br>• DSM-IV-diagnosis of major depressive disorder<br><br>Exclusion criteria:<br>• Bipolar disorder (DSM-IV)<br>• Psychotic features or any psychotic disorder as defined in the DSM-IV<br>• Current psychopharmacological treatment, including antidepressant medication or other psychotropic medication<br>• Pregnant or breastfeeding women

**Measurements and procedures:**	Patients diagnosed with major depression will be investigated in a single-centre, double-blind, placebo-controlled, parallel-group design clinical trial contrasting the acute and persisting effects of psilocybin to those of placebo in major depression. Patients will be randomly assigned to psilocybin or placebo group with a 1:1 allocation ratio.<br><br>The study comprises a total of 7 visits during a total study duration of 3-8 weeks and includes the following visits:<br>• screening visit<br>• pre-investigation visit<br>• baseline measures visit<br>• treatment visit<br>• post-treatment measures visit<br>• follow-up visit<br>• follow-up measures & closure visit<br><br>In addition, two follow-up measurements will guarantee monitoring of long-lasting changes in symptomology and ensure all potential side-effects can be captured. It is implemented as an anonymous online-survey that can be done from home. The assessments will take place 6 weeks after the administration of the substance (45 days post-treatment), three months and six months post-treatment, respectively.<br><br>The following primary and additional outcome measurements will be conducted at screening, at baseline, and following drug administration (2, 8, and 14 days post-treatment):<br>• Montgomery Asberg Depression Rating Scale<br>• Hamilton Anxiety Rating Scale<br>• Clinical Global Impressions Scale<br><br>The following secondary outcome measurements will be conducted at baseline, and following drug administration (2 and 14 days post-treatment):<br>• Multifaceted Empathy Task<br>• Resting State FMRI<br>• FMRI Amygdala Reactivity Paradigm<br>• FMRI Negative Feedback Paradigm (Probalistic Reversal Learning Task)<br><br>The following secondary outcome measurements will be conducted at baseline, and following drug administration (2 and 8 days post-treatment):<br>• Brain Derived Neurotrophic Factor (BDNF)<br><br>The following secondary outcome measurements will be conducted at screening, at baseline, and following drug administration, as well as during the 6 month follow-up survey (2, 8, 14, 42, 84 and 168 days post-treatment):<br>• Snaith-Hamilton-Pleasure Scale<br>• Hamilton Anxiety Scale<br>• Rumination-Reflection Questionnaire<br>• Hopelessness Scale
**Study Product / Intervention:**	Psilocybin (0.215mg/kg orally); treatment arm 1; one-time application of a single dose
**Control Intervention (if applicable):**	Placebo (100% mannitol orally); treatment arm 2; one-time application of a single dose

**Number of Participants with Rationale:**	30 patients in arm 1 and 30 patients in arm 2, total N=60 (determined by power analysis)
**Study Duration:**	4 years
**Study Schedule:**	First-patient-in: 01/2015 (planned)<br>Last-patient-out: 01/2019 (planned)
**Investigator(s):**	Franz X. Vollenweider, Prof. Dr. med. (Sponsor- & Principal Investigator)<br>Department of Psychiatry, Psychotherapy and Psychosomatics<br>Neuropsychopharmacology and Brain Imaging<br>Psychiatric Hospital, University of Zurich<br>Lenggstrasse 31, ZH-8032 Zürich, Switzerland<br>E-mail: vollen@bli.uzh.ch<br>Phone: +41 44 384 24 04<br><br>Dr. med. Andres Ort<br>Department of Psychiatry, Psychotherapy and Psychosomatics<br>Psychiatric Hospital, University of Zurich<br>Lenggstrasse 31<br>CH-8032 Zürich,<br>Switzerland<br>E-mail: sekretariat.seifritz@bli.uzh.ch<br>Phone: +41 44 384 23 12<br><br>Erich Seifritz, Prof. Dr. med. (Co-Investigator)<br>Department of Psychiatry, Psychotherapy and Psychosomatics<br>Psychiatric Hospital, University of Zurich<br>Lenggstrasse 31<br>CH-8032 Zürich,<br>Switzerland<br>E-mail: sekretariat.seifritz@bli.uzh.ch<br>Phone: +41 44 384 23 12<br><br>Katrin Preller, Dr. phil. (Co-Investigator)<br>Department of Psychiatry, Psychotherapy and Psychosomatics<br>Neuropsychopharmacology and Brain Imaging<br>Psychiatric Hospital, University of Zurich<br>Lenggstrasse 31<br>CH-8032 Zürich,<br>Switzerland<br>E-mail: preller@bli.uzh.ch<br>Phone: +41 44 384 26 25<br><br>Robin von Rotz, MSc (Co-Investigator)<br>Department of Psychiatry, Psychotherapy and Psychosomatics<br>Neuropsychopharmacology and Brain Imaging<br>Psychiatric Hospital, University of Zurich<br>Lenggstrasse 31<br>CH-8032 Zürich,<br>Switzerland<br>E-mail: robin.vonrotz@bli.uzh.ch<br>Phone: +41 44 384 26 16
**Study Centre(s):**	Single centre: Department of Psychiatry, Psychotherapy and Psychosomatics, Neuropsychopharmacology and Brain Imaging, Psychiatric Hospital, University of Zurich, Switzerland

**Statistical Considerations:**	The effects of psilocybin (compared to placebo) will be analyzed statistically using mixed-model analyses of variance (ANOVA). The statistical threshold for all analyzes will be $p<0.05$. Data analysis of fMRI data will be performed using GLM as implemented in SPM (Statistical Parametric Mapping, Wellcome Department of Cognitive Neurology, London, UK). Sample size is determined by power analysis.
**GCP Statement:**	This study will be conducted in compliance with the protocol, the current version of the Declaration of Helsinki, the ICH-GCP as well as all national legal and regulatory requirements.

3 ABBREVIATIONS

5-HT	5-Hydroxytryptamine, Serotonin
AE	Adverse Event
AMPA-R	Amino-3-hydroxy-5-methyl-4-isoxazolepropionic acid, a glutamate receptor
BOLD	Blood Oxygenation Level Dependent
BDNF	Brain Derived Neurotrophic Factor
CA	Competent Authority (e.g. Swissmedic)
CEC	Competent Ethics Committee
ClinO	Clinical Trials Ordinance
CRF	Case Report Form
eCRF	Electronic Case Report Form
CTCAE	Common terminology criteria for adverse events
DSUR	Development safety update report
ECG	Electrocardiography
FMRI	Functional Magnetic Resonance Imaging
GCP	Good Clinical Practice
H0	Null hypothesis
H1	Alternative hypothesis
ICH	International Conference on Harmonization
IMP	Investigational Medicinal Product
ISF	Investigator Site File
ITT	Intention to Treat
LHR	Law on human research
MDD	Major Depressive Episode
NMDA-R	N-methyl-D-aspartate receptor, a glutamate receptor
PI	Principal Investigator
SAE	Serious Adverse Event
SDV	Source Data Verification
SmPC	Summary of Product Characteristics
SNCTP	Swiss National Clinical Trial Portal
SOP	Standard Operating Procedure
SSRI	Selective Serotonin Reuptake Inhibitor
SUSAR	Suspected Unexpected Serious Adverse Reaction
TMF	Trial Master File

4 STUDY SCHEDULE

Flow chart of study schedule:

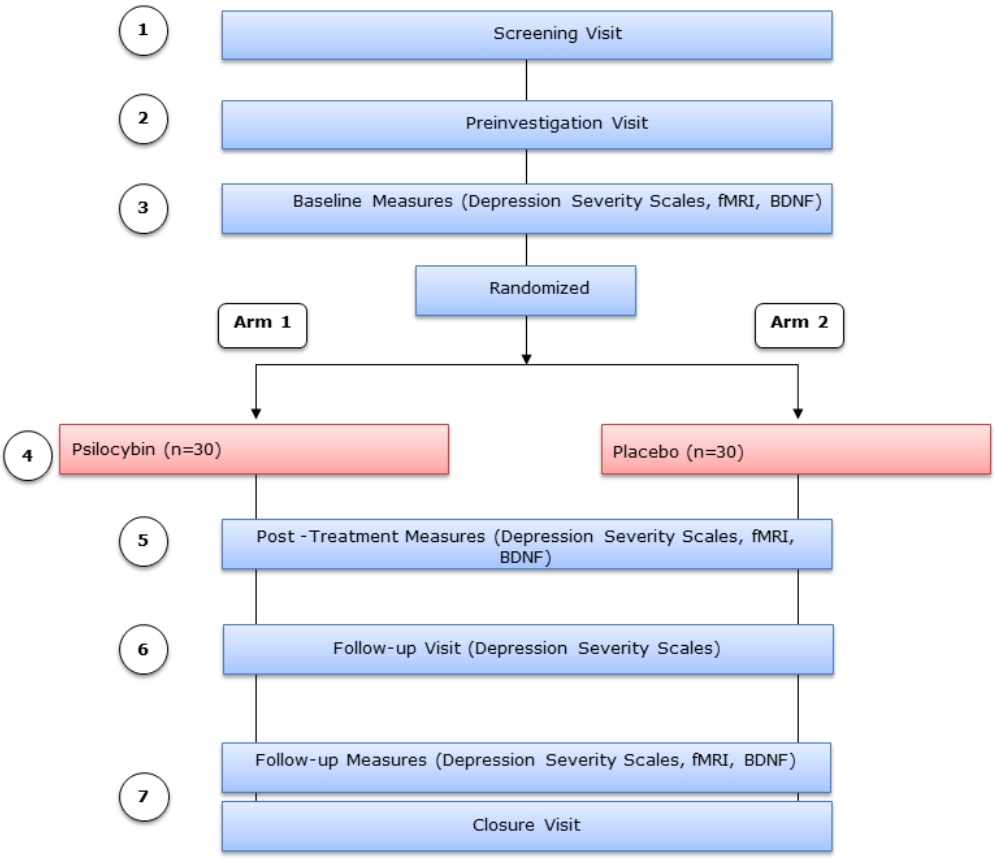

Tabular listing of study schedule:

Study Periods	*Screening/ Preinvestigation Visits*			*Treatment Period*	*Postinvestigation Visits*			*Follow-up surveys*		
Visit	*1*	*2*	*3*	*4*	*5*	*6*	*7*	*8*	*9*	*10*
Time (t)	*t0-42d (-/37d)*	*t0-4d (+/-2d)*	*t0-24h (+/-0d)*	*t0*	*t0+48h (+/- 0d)*	*t0+8d (+/-1d)*	*t0+14d (+/-2d)*	*t0+42d (+/-5d)*	*t0+84d (+/-5d)*	*t0+168d (+/- 5d)*

Patient Information and Informed Consent	x									
Biographical Anamnesis	x									
Medical/ Psychiatric History, including Family History	x									
Suicidality (C-SSRS)	x	x	x	x	x	x	x			
Symptom Checklist (SCL 90-R)	x							x	x	x
Structured Clinical Interview (SCID I and II)	x									
Frankfurt Self-Concept Scale (FSCS)	x						x	*x*	*x*	*x*
Verbal IQ (MWT-B)	x									
In-/Exclusion Criteria	x									
MRI Safety Form			x		x		x			
Physical Examination	x									
Echocardiogram	x									
Vital Signs	x	x	x	x	x	x	x			

Body Weight	x	x	x	x	x	x	x			
Concomitant Medication Form	x	x	x	x	x	x	x	x	x	x
Drug Screening	x		x		x		x	x	x	x
Drug Urine Test	x		x		x		x			
Routine Laboratory Tests[1]	x									
Urine Pregnancy Test[2] *(Women only)*	x									
Randomizati on			x							
Psychologic al Counselling Session		x	x	x	x	x	x			
Tape Recordings	x	x	x	x	x	x	x			
Résumé written by participants	x									
Protocol of subjective experiencing during drug treatment session, written by participants				x						
Administer Study Medication				x						

[1] Hemoglobin, white blood cell count (WBC), sodium (Na), potassium (K), calcium (Ca), glucose, creatinine, aspartate aminotransferase (AST), alanine aminotransferase (ALT), gamma-glutamyltransferase (GGT), thyroid stimulating hormone (TSH)

[2] human corionic gonadotropin (hCG)

Altered States of Consciousness Questionnaire (5D-ASC)				x						
Stundenbogen (STEPP, STEPT)		x	x	x	x	x	x			
FMRI tasks			x		x		x			
Multifaceted Empathy Task			x		x	x	x			
Blood & Serum Brain-Derived Neurotrophic Factor (BDNF) levels			x	x	x	x				
Relaxation exercise		x	x	x	x	x	*x*			
Clinical Global Impressions (CGI)	x	x	x	x	x	x	x			
Montgomery-Asberg Depression Scale (MADRS)	x	x	x	x	x	x	x			
Emotion-Regulation Questionnaire			x		x		x	x	x	x
Beck Depression Inventory (BDI)	x	x	x	x	x	x	x	x	x	x

Snaith-Hamilton-Pleasure Scale (SHAPS)			x	x	x	x	x	x	x	x
Hamilton Anxiety Scale (HAMA)	*x*	*x*	x	x	x	x	x			
Rumination-Reflection Questionnaire			x	x	x	x	x	x	x	x
Hopelessness Scale (HS-Krampen)			x	x	x	x	x	x	x	x
Treatment Emergent Symptom Scale (DOTES, TWIS)/ Adverse Events	x	x	x	x	x	x	x			
Therapy-Questionnaire	x							x	x	x
Stress Questionnaire								x	x	x

5 STUDY ADMINISTRATIVE STRUCTURE

5.1 Sponsor-Investigator and Principal Investigator

Franz X. Vollenweider, Prof. Dr. med.
Department of Psychiatry, Psychotherapy and Psychosomatics
Neuropsychopharmacology and Brain Imaging
Psychiatric Hospital, University of Zurich
Lenggstrasse 31, ZH-8032 Zürich, Switzerland

E-mail: vollen@bli.uzh.ch
Phone: +41 44 384 24 04

5.2 Co-Investigators

Andres Ort, Dr. med.
Department of Psychiatry, Psychotherapy and Psychosomatics
Neuropsychopharmacology and Brain Imaging
Psychiatric Hospital, University of Zurich
Lenggstrasse 31
CH-8032 Zürich,
Switzerland
E-mail: andres.ort@bli.uzh.ch
Phone: +41 44 384 26 03

Erich Seifritz, Prof. Dr. med.
Department of Psychiatry, Psychotherapy and Psychosomatics
Psychiatric Hospital, University of Zurich
Lenggstrasse 31
CH-8032 Zürich,
Switzerland
E-mail: sekretariat.seifritz@bli.uzh.ch
Phone: +41 44 384 23 12

Katrin Preller, Dr. phil.
Department of Psychiatry, Psychotherapy and Psychosomatics
Neuropsychopharmacology and Brain Imaging
Psychiatric Hospital, University of Zurich
Lenggstrasse 31
CH-8032 Zürich,
Switzerland
E-mail: preller@bli.uzh.ch
Phone: +41 44 384 26 25

Robin von Rotz, MSc
Department of Psychiatry, Psychotherapy and Psychosomatics
Neuropsychopharmacology and Brain Imaging
Psychiatric Hospital, University of Zurich
Lenggstrasse 31
CH-8032 Zürich,
Switzerland
E-mail: robin.vonrotz@bli.uzh.ch
Phone: +41 44 384 26 16

5.3 Statistician (“Biostatistician”)

Katrin Preller

Department of Psychiatry, Psychotherapy and Psychosomatics
Neuropsychopharmacology and Brain Imaging
Lenggstrasse 31
CH-8032 Zürich,
Switzerland
Phone: +41 44 384 26 25
E-mail: preller@bli.uzh.ch

5.4 Laboratory

5.4.1 On-Site Laboratory

Albina Nowak, PD Dr. (Head)
University Hospital of Psychiatry
Medical Centre/ Laboratory
Lenggstrasse 31
CH-8032 Zürich,
Switzerland
Phone +41 (0)44 384 23 21

Our on-site laboratory will analyze routine laboratory tests, drug urine test, and pregnancy test. These laboratory tests are used for medical screening and study protocol adherence (drug urine repeat) purposes. All samples taken will be immediately destroyed after analysis. Routine laboratory tests include the following parameters:

- Hemoglobin
- white blood cell count (WBC)
- sodium (Na)
- potassium (K)
- calcium (Ca)glucose
- creatinine
- aspartate aminotransferase (AST)
- alanine aminotransferase (ALT)
- gamma-glutamyltransferase (GGT)
- thyroid stimulating hormone (TSH)

5.4.2 Collaboratoring Laboratory

Anne Eckert, Prof., PhD (Head)
Neurobiology Laboratory
For Brain Aging and Mental Health
Assoc. Res. Group Dept. Biomedicine Univ. of Basel
Psychiatric University Clinics Basel
Wilhelm Klein-Strasse 27
CH-4025 Basel
Phone: +41 61 325 54 87

Fax: +41 61 325 55 77
E-mail: anne.eckert@upkbs.ch

Our collaborating laboratory will analyze plasma and serum brain-derived neurotrophic factor (BDNF) levels. Plasma and serum BDNF levels are unrelated to any safety issues for this study. All samples taken will be immediately destroyed after analysis.

5.5 Monitoring Institution

Clinical Trial Unit
Universität Basel
Departement Klinische Forschung
c/o Universitätsspital Basel
Schanzenstrasse 55
4031 Basel, Schweiz

The monitoring institution will monitor study data according to GCP, and as documented in separate documents: 1) the Monitoring Agreement, 2) the Monitoring Plan & Checklist (see "Anhang 12, Monitoring" for these documents. The CTU is independent from the Sponsor, and there are no competing interests.

5.6 Any other relevant Committee, Person, Organisation, Institution

Not applicable.

6 ETHICAL AND REGULATORY ASPECTS

Before this study will be conducted, the protocol, the proposed participant information and consent form as well as other study-specific documents will be submitted to a properly constituted Competent Ethics Committee (CEC) and Competent Authorities (Swissmedic/BAG) in agreement with local legal requirements, for formal approval. Any amendment to the protocol must as well be approved.

The decision of the CEC and Swissmedic/BAG concerning the conduct of the study will be made in writing to the Sponsor-Investigator before commencement of this study. The clinical study can only begin once approval from all required authorities has been received. Any additional requirements imposed by the authorities shall be implemented.

6.1 Study Registration

The study will be registered in the international trial registry ClinicalTrials.gov (NCT03715127). Additionally, the study will be registered in the national study database SNCTP (Swiss National Clinical Trials Portal) on www.kofam.ch.

6.1.1 Birth date

According to new data protection legislation, the use of complete birth date on study forms is not allowed anymore.

6.2 Categorisation of Study

This clinical trial comes under the Category C (ClinO Art. 19) because psilocybin is not authorized as a medical product in Switzerland.

6.3 Competent Ethics Committee (CEC)

Approval from the appropriate constituted Competent Ethics Committee is sought for each study site in the clinical trial. The reporting duties and allowed time frame are respected. No substantial changes are made to the protocol without prior Sponsor, CEC, CA approval, except where necessary to eliminate apparent immediate hazards to study participants.

Premature study end or interruption of the study is reported within 15 days. The regular end of the study is reported to the CEC within 90 days, the final study report shall be submitted within one year after study end. Amendments are reported according to chapter 2.10.

6.4 Competent authority (CA)

The Sponsor will obtain approval from Swissmedic before the start of the clinical trial. Reporting will be done within the allowed time frame.

Planned or premature study end are reported within 90 and 15 days, respectively. The final report will be submitted to the CA within one year after the end of the study. Amendments are reported according to chapter 2.10.

6.5 Ethical Conduct of the Study

The study will be carried out in accordance with principles enunciated in the current version of the Declaration of Helsinki, the guidelines of Good Clinical Practice (GCP) issued by ICH, and Swiss competent authority's requirements. CEC and competent authorities will receive annual safety and interim reports and be informed about study stop/ end in agreement with local requirements.

6.6 Declaration of Interest

All investigators declare no conflict of interest.

6.7 Patient Information and Informed Consent

The investigator must explain to each participant the nature of the study, its purpose, the procedures involved, the expected duration, the potential risks and benefits and any discomfort it may entail. Each participant must be informed that the participation in the study is voluntary and that he/she may withdraw from the study at any time and that withdrawal of consent will not affect his/her subsequent medical treatment.

The participant must be informed that his/her medical records may be examined by authorized individuals other than their treating physician.

All participants for this study will be provided a participant information sheet and a consent form describing this study and providing sufficient information for participants to make an informed decision about their participation in this study.

The participant information sheet and the consent form will be submitted with the protocol for review and approval for the study by the CEC and by Swissmedic. The formal consent of a participant, using the approved consent form, must be obtained before that participant is submitted to any study procedure.

The participant should read and consider the statement before signing and dating the informed consent form, and should be given a copy of the signed document. The consent form must also be signed and dated by the investigator (or his designee) and it will be retained as part of the study records.

Before participants make their final decision on study participation, they must have at least 24 hours time for consideration.

Prior to their enrolment, participants will be asked for consent concerning the potential re-use of their data. This serves to maximize the scientific value of the acquired data and to avoid unnecessary repetitions of measurements, thus reducing the burden on the participants should they wish to take part in future studies.

6.8 Participant Privacy and Confidentiality

The investigator affirms and upholds the principle of the participant's right to privacy and that they shall comply with applicable privacy laws. Especially, anonymity of the participants shall be guaranteed when presenting the data at scientific meetings or publishing them in scientific journals.

Individual subject medical information obtained as a result of this study is considered confidential and disclosure to third parties is prohibited. Subject confidentiality will be further ensured by utilising subject identification code numbers to correspond to treatment data in the computer files.

Such medical information may be given to the participant’s personal physician or to other appropriate medical personnel responsible for the participant’s welfare, if the patient has given his/her written consent to do so.

For data verification purposes, authorised representatives of the Sponsor (-Investigator), a competent authority (e.g. Swissmedic), or an ethics committee may require direct access to parts of the medical records relevant to the study, including participants’ medical history.

6.9 Early Termination of the Study

The Sponsor-Investigator may terminate the study prematurely according to certain circumstances, e.g.:

- ethical concerns,
- insufficient participant recruitment,
- when the safety of the participants is doubtful or at risk, respectively,
- alterations in accepted clinical practice that make the continuation of a clinical trial unwise,
- early evidence of benefit or harm of the experimental intervention

6.10 Protocol Amendments

The Principal Investigator, Prof. Dr. med. Franz Vollenweider, is allowed to amend the protocol or to provide suggestions for a protocol amendment. Important protocol modifications (e.g., changes to eligibility criteria, outcomes, analyses) will be communicated to all relevant parties (e.g., investigators, CEC, competent authorities, trial participants, trial registries, journals, regulators), if applicable. Substantial amendments are only implemented after approval of the CEC and CA respectively. Under emergency circumstances, deviations from the protocol to protect the rights, safety and well-being of human participants may proceed without prior approval of the sponsor and the CEC/CA. Such deviations shall be documented and reported to the sponsor and the CEC/CA as soon as possible. All Non-substantial amendments are communicated to the CA as soon as possible if applicable and to the CEC within the Annual Safety Report (ASR).

7 INTRODUCTION

The majority of existing therapies for major depression increase levels of brain monoamine neurotransmitters such as serotonin and norepinephrine (typically by inhibiting reuptake, e.g. selective serotonin reuptake inhibitors, SSRIs). Although a substantial number of depressed patients show reduced or remitted depression symptoms after treatment with these medications, approximately 30-50% of patients fail to respond fully and as many as 10-30% remain resistant to these and other treatment options. A growing body of evidence (Molendijk et al. 2011; Brunoni et al. 2008; Wolkowitz et al. 2011) suggests that traditional antidepressants, as well as novel medications effective in depression, exert their therapeutic efficacy via the indirect, downstream action of glutamate and neurotrophins, such as the brain-derived neurotrophic factor (BDNF), which has been related to neuronal survival, synaptic signalling and synaptic consolidation (Sanacora et al. 2008; Skolnick et al. 2009; Cryan und O'Leary 2010; Duman und Voleti 2012; Brunoni et al. 2008; Molendijk et al. 2011; Sanacora et al. 2008) (**Figure 1**).

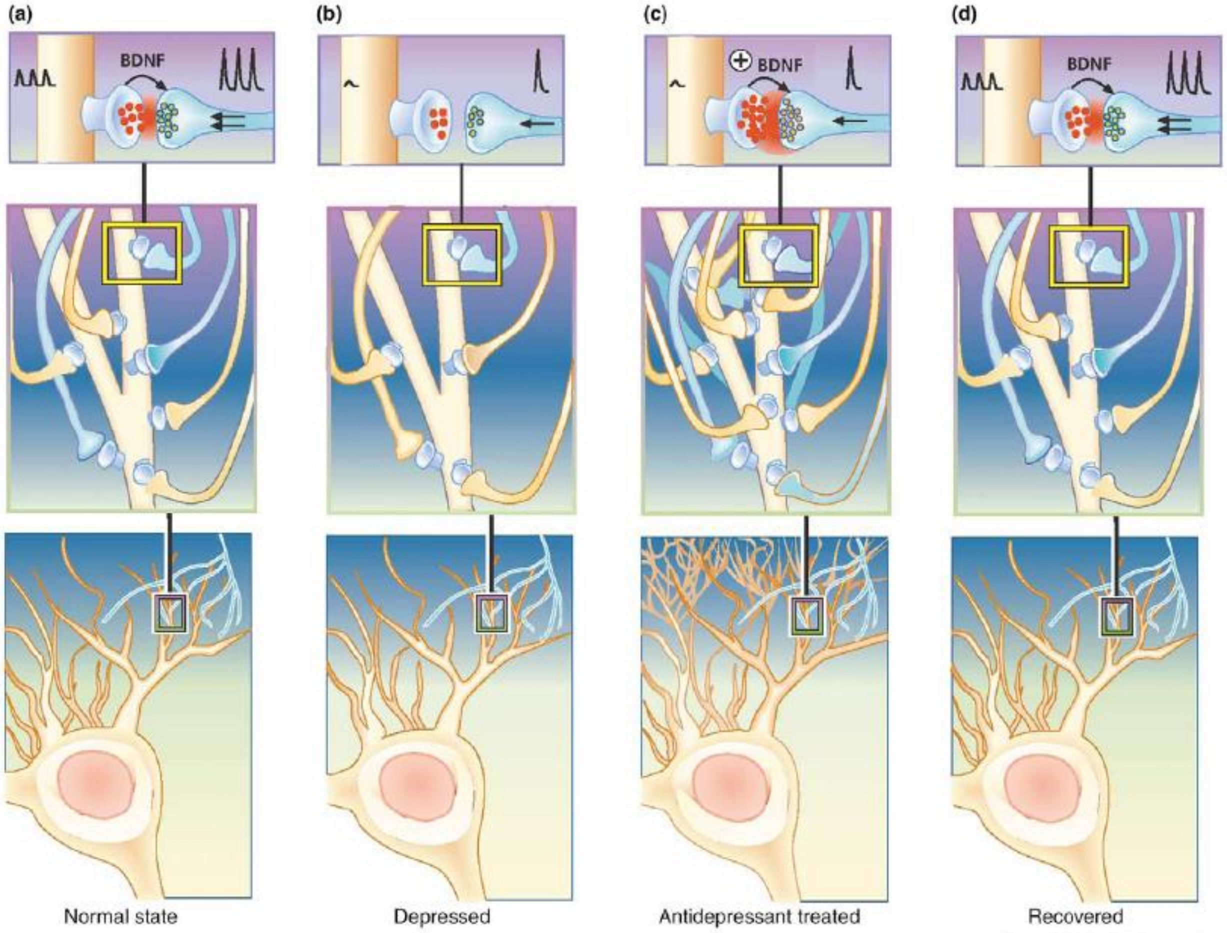

Figure 1. Effect of antidepressant treatment on activity-dependent connectivity in the human brain. In depression, connectivity is reduced because of insufficient activity-dependent BDNF-release (b). Antidepressant treatment enhances BDNF release which leads to use-dependent pruning of inactive synaptic contacts and synaptogenesis (c). This eventually stabilizes the neurocircuitry underlying emotion processing (such as the amygdala, hippocampus, and prefrontal cortex) and leads to complete mood recovery (d) (Castrén 2004).

The most effective and well-researched glutamate-based antidepressant is ketamine, a nonselective NMDA-receptor antagonist. Several double-blind, placebo-controlled studies have demonstrated the efficacy of ketamine in producing rapid (within hours) but time-limited (up to 1 week) antidepressant effects that are statistically and clinically robust in patients with treatment-resistant major depression and bipolar disorder (Berman et al. 2000; Zarate et al. 2006b; Zarate et al. 2006a; Diazgranados et al. 2010a; Diazgranados et al. 2010b) (**Figure 2**).

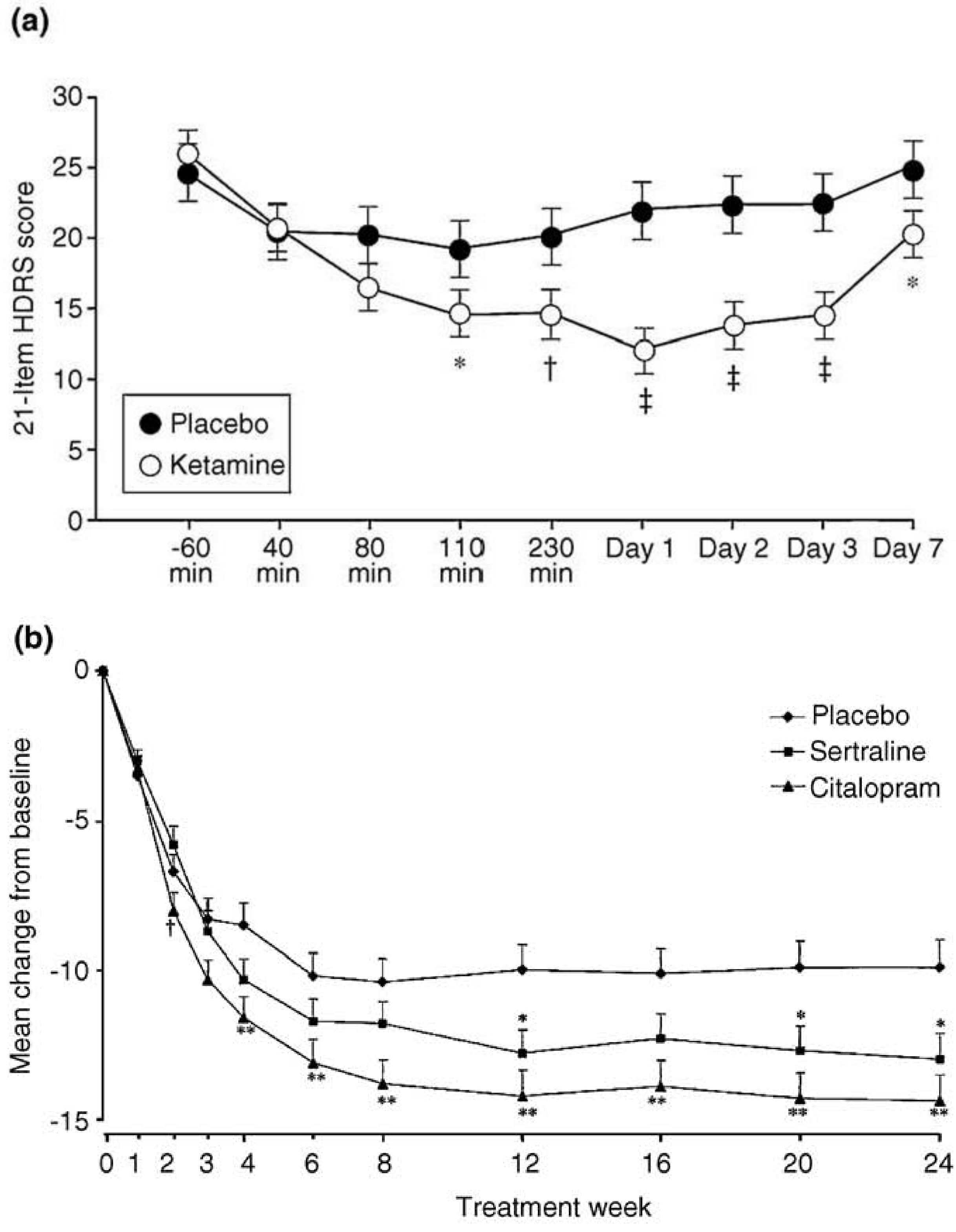

Figure 2. Comparison between antidepressant effects of ketamine (a) and SSRIs (b) in depressed patients. Ketamine (single dose) decreases depression score (HDRS) rapidly (within hours), whereas SSRIs (continuous treatment) do not (Skolnick et al. 2009).

Additional open-label or non-placebo-controlled studies of ketamine in specific patient populations (e.g., acute suicidal ideation; ECT-resistant major depression) have provided

preliminary data suggestive of rapid antidepressant effects within 4 hours of infusion (Phelps et al. 2009; Price et al. 2009; Diazgranados et al. 2010a; Diazgranados et al. 2010b). Pre-clinical work suggests that the mechanistic pathway for these therapeutic effects is likely an increase in glutamate release, followed by stimulation of AMPA receptors (relative to NMDA receptors, which are blocked by ketamine), and subsequent AMPA/ glutamate-driven neuroplasticity, including synthesis of brain-derived neurotrophic factor (BDNF) and synaptogenesis (Moghaddam et al. 1997; Maeng et al. 2008; Li et al. 2010; Liu et al. 2012). In support of this view, a recent clinical study (Haile et al. 2014) demonstrated that ketamine significantly increased plasma BDNF levels in responders compared to non-responders 240 min post-treatment, and that Montgomery-Asberg Depression Scale (MADRS) scores were negatively correlated with BDNF (**Figure 3**).

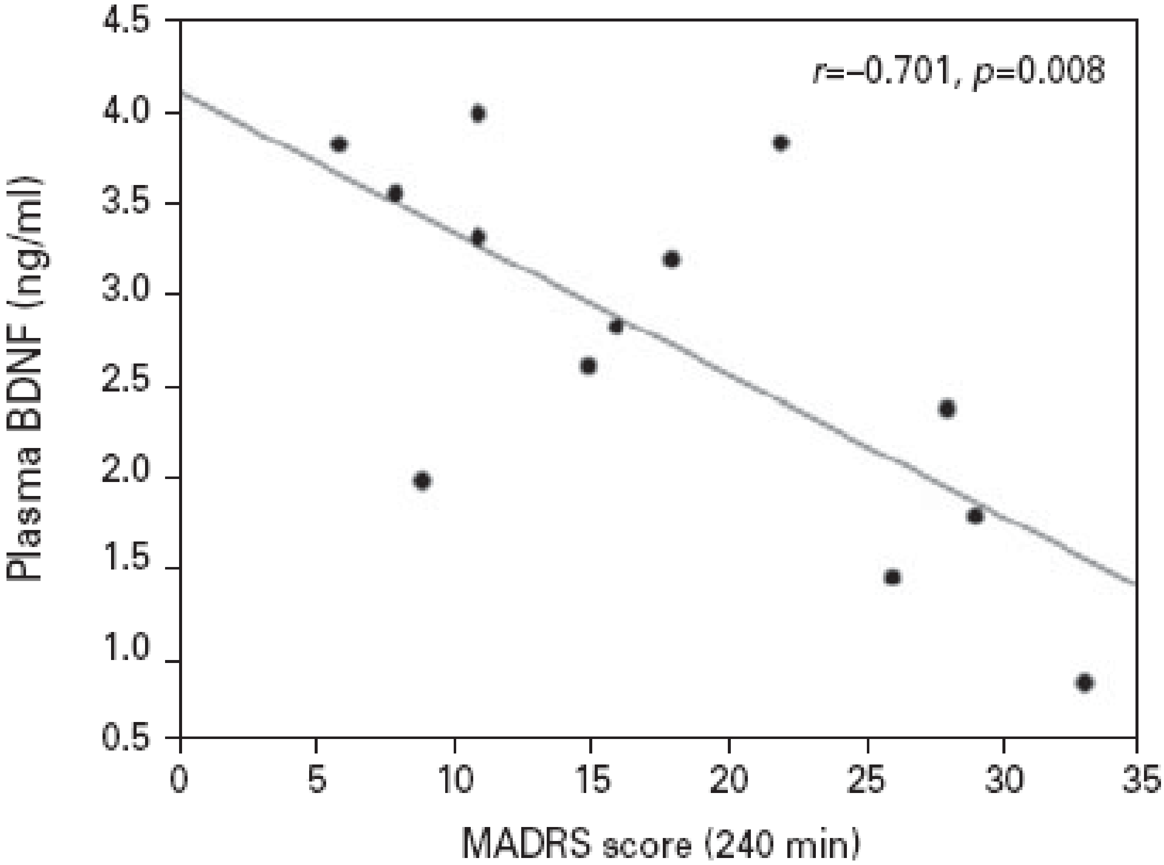

Figure 3. Plasma BDNF-levels and depression score (MADRS) 240 minutes after a single dose of ketamine in depressed patients. Increase of plasma BDNF-levels was strongly correlated with decrease of depression scores (Haile et al. 2014).

Most interestingly, preclinical animal and human studies suggest that serotonin (5-HT) receptor agonists such as LSD and psilocybin have a similar glutamate- and BDNF-enhancing effects in layer V of the prefrontal cortex (Muschamp et al. 2004; Vollenweider und Kometer 2010b) (**Figure 4**).

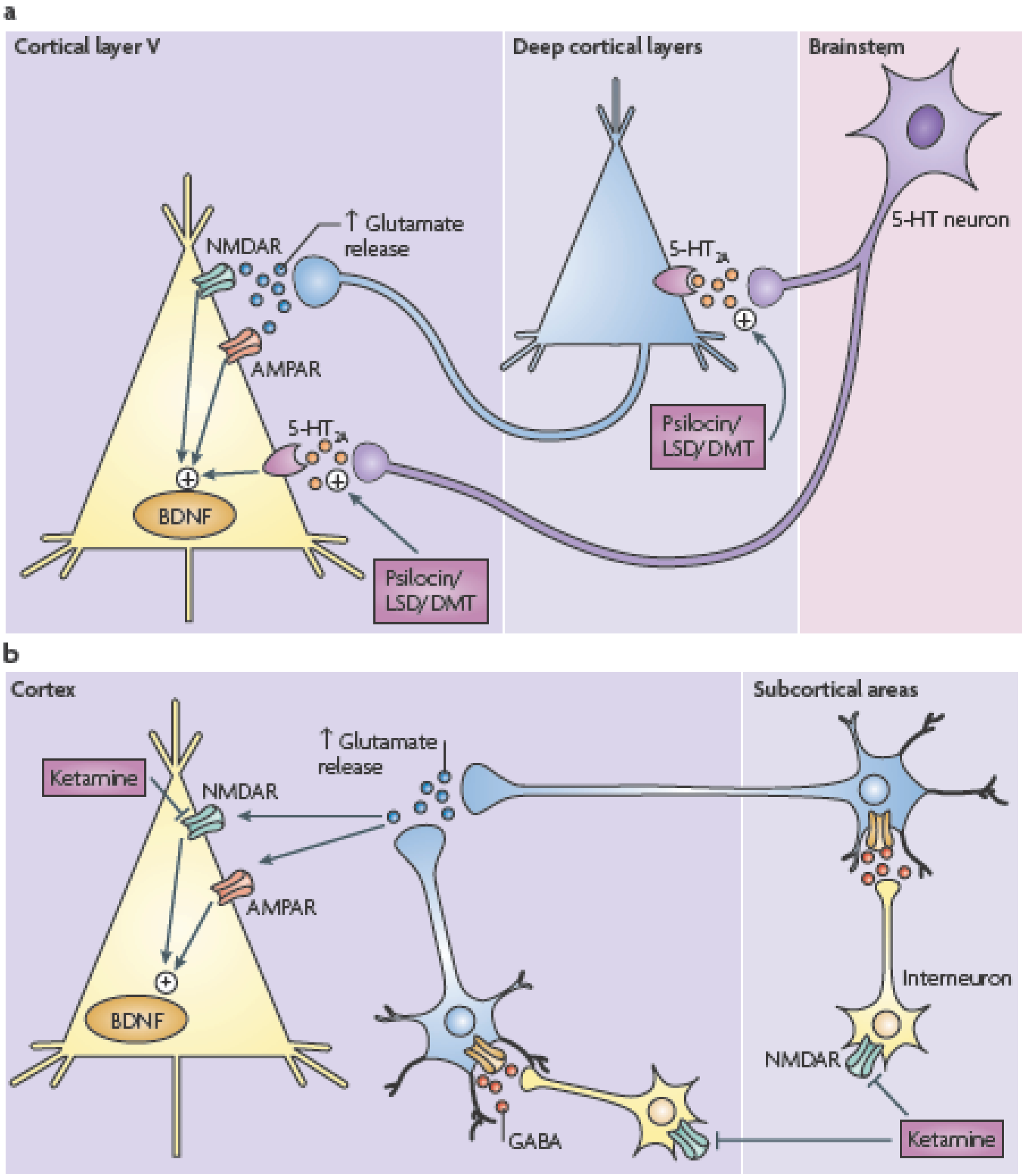

Figure 4. Activation of the prefrontal cortex by psilocybin (a) and ketamine (b). Both substances increase glutamate-release in PFC, leading to AMPAR activation and release of BDNF. In addition, psilocybin directly activates 5-HT2A receptors of layer V pyramidal neurons, which additionally stimulates BDNF-release (Vollenweider und Kometer 2010b).

Congruent with these findings, human neuroimaging studies have shown that both psilocybin and ketamine activate metabolic activity within certain prefrontal cortical areas which are thought to have a top-down control over emotion and stress responses through its connections to the amygdala (Vollenweider et al. 1998; Vollenweider et al. 1997). Reduced prefrontal glutamate levels in the PFC have recently been reported in patients with major depression (Hasler et al. 2007; Walter et al. 2009). On the other hand it has

been shown that treatment with selective serotonin reuptake inhibitors (SSRIs) modulates neural activity within key regions of the emotion processing circuits, such as the amygdala and the PFC (Outhred et al. 2013) (**Figure 5**). Furthermore, SSRIs increase the functional connectivity between the amygdala and the PFC resulting in an attenuation of the amygdala during emotional and self-referential tasks in patients with depression (Fu, Cynthia H Y et al. 2004; Grimm et al. 2009; Nejad et al. 2013).

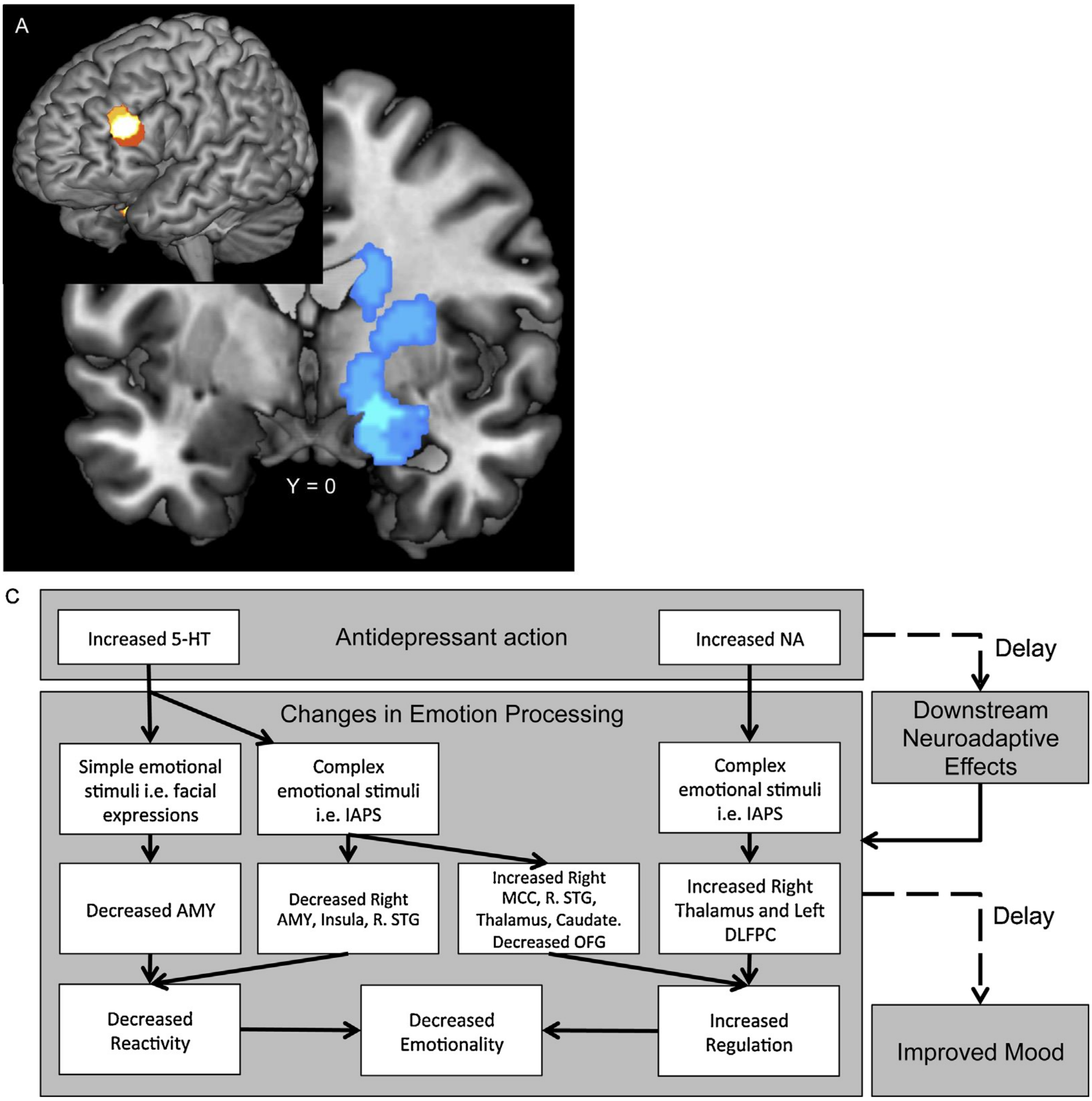

Figure 5. Meta-analysis of acute effects of SSRIs on emotion neurocircuits in healthy subjects. SSRIs increase left dorsolateral prefrontal cortex (DLPFC) and decrease right amydaloid-hippocampal region (A). This eventually leads to a decrease of amygdala hyperactivity and an increase of mood regulation (Outhred et al. 2013).

Along this line we (Schmidt et al. 2013; Kometer et al. 2012; Bernasconi et al. 2013; Kraehenmann et al. 2014) have recently shown that a single dose of psilocybin rapidly induces positive mood and reduces electrophysiological and blood oxygenation level-dependent (BOLD) fMRI responses to negative stimuli in the amygdala and the medial

PFC in healthy subjects. In addition, Carhart-Harris et al. (Carhart-Harris et al. 2012b) assessed the acute effects of psilocybin during resting state using task-free BOLD fMRI in healthy volunteers. They showed that psilocybin deactivates the medial PFC (and other default mode network regions, such as the posterior cingulate cortex)- a hub region known to be hyperactive in depression and normalized after effective treatment (**Figure 6**).

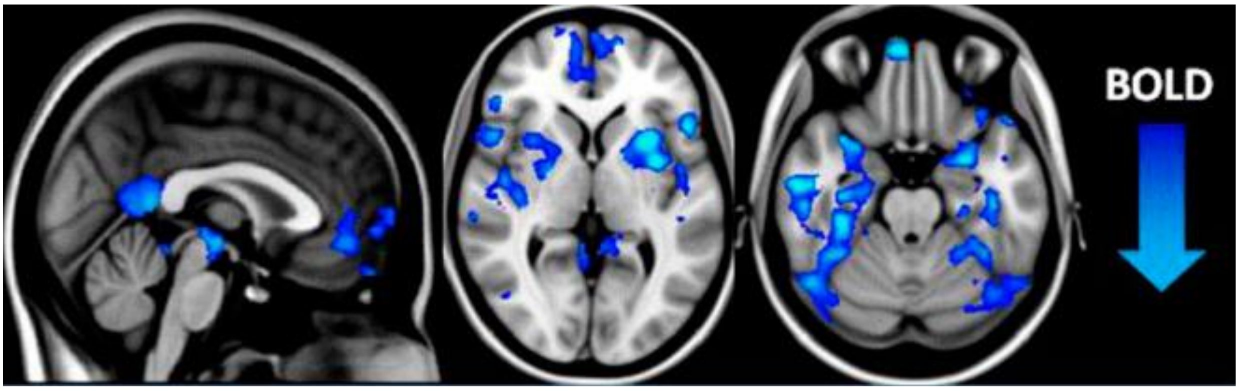

Figure 6. Brain resting state deactivations after psilocybin. A single dose of psilocybin decreases activity in PFC and PCC (Carhart-Harris et al. 2012b).

Finally, Kraehenmann et al. (Kraehenmann et al. 2014) assessed the effects of acute administration of a low-to moderate dose of psilocybin (.16mg/kg) on mood state and amygdala reactivity using BOLD fMRI in a double-blind, randomized, placebo-controlled, cross-over study in 25 healthy volunteers. They found that acute treatment with psilocybin decreased amygdala reactivity during negative-emotion processing, and that this is associated with a parallel increase of positive mood state (**Figure 7**).

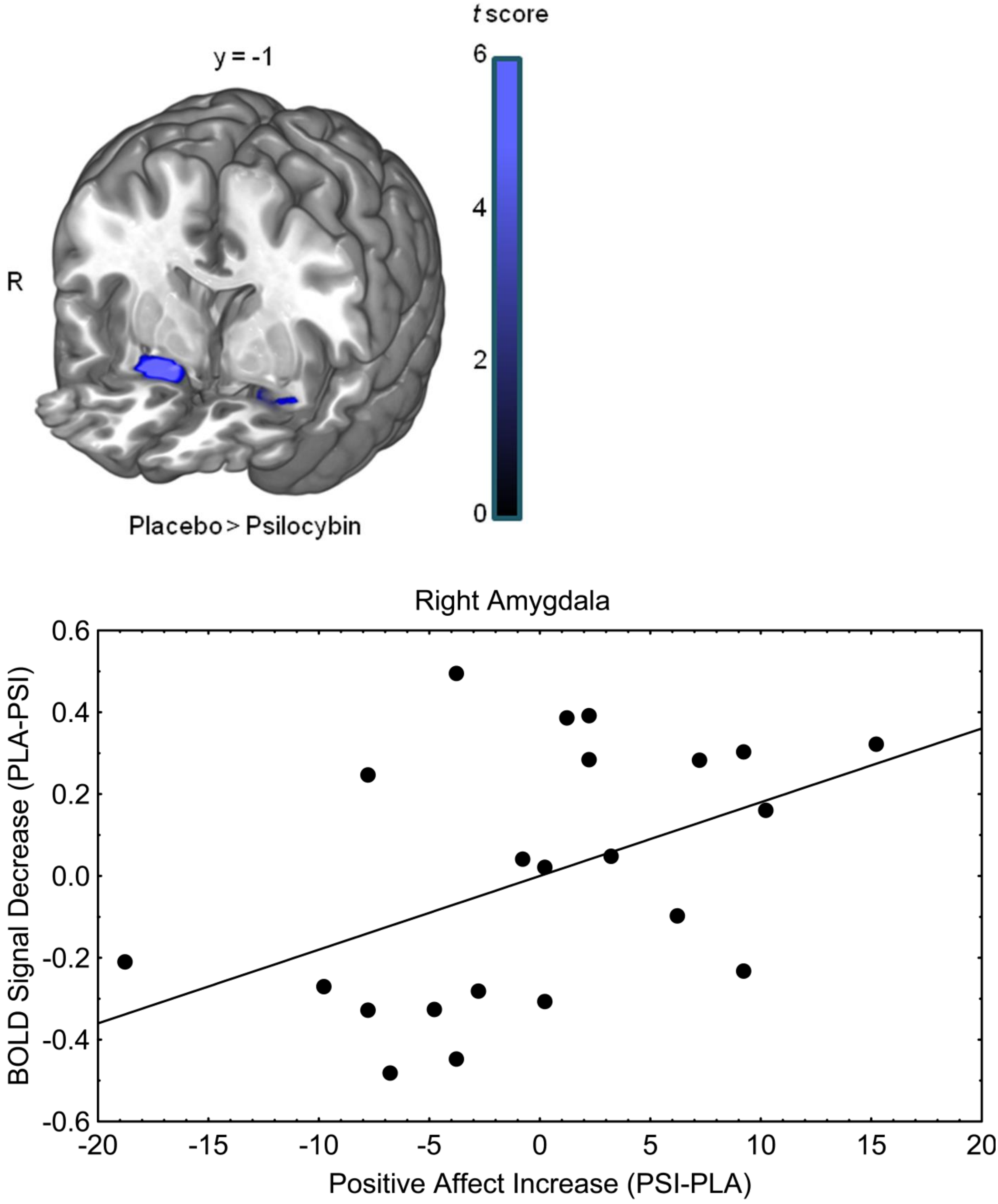

Figure 7. Psilocybin effects during emotion processing. Psilocybin decreases amygdala reactivity to negative stimuli in relation to positive-mood induction (Kraehenmann et al. 2014).

Taken together, broad preclinical evidence (Vollenweider et al. 1998; Vollenweider et al. 1997; Catlow et al. 2013; Bernasconi et al. 2013; Schmidt et al. 2013; Kometer et al. 2012; Kraehenmann et al. 2014; Griffiths et al. 2011; Griffiths et al. 2006a; Griffiths et al. 2008b) indicates that psilocybin has antidepressant efficacy, as it rapidly reduces neural responses to negative stimuli and enhances prefrontal top-down control over limbic

regions which may be important for a fast recovery from depression. In support of this view, a recent clinical trial (Grob et al. 2011a) of the effect of psilocybin in patients with depression and anxiety related to advanced-stage cancer found that a single dose of psilocybin significantly decreased anxiety and increased positive mood state for up to 6 months. Importantly, thousands of scientific papers published by 1965 described positive results in more than 40’000 patients who had taken psilocybin (formerly known as Sandoz’ Indocybin®) with minimal side effects and a high level of safety (Tylš et al. 2014; Baumeister et al. 2014). Compared to the antidepressant ketamine, psilocybin may have some advantages in regard to its acute and long-term effects. In particular, preclinical and clinical studies indicate that psilocybin does not lead to emotional blunting and may have longer lasting antidepressant effects than ketamine (see Table below).

**Characteristics**	**Ketamine**	**Psilocybin**
Antidepressant efficacy	Rapid, but lasting only 1 week (Murrough et al. 2013; Zarate et al. 2006a)	To be investigated in this study; presumably rapid and lasting >2 weeks (Grob et al. 2011a; Griffiths et al. 2011)
Emotional blunting	Yes (Naughton et al. 2014)	No (Schmidt et al. 2013)
Cognitive deficits (mismatch negativity)	Yes (Naughton et al. 2014)	No (Schmidt et al. 2013)
Abuse potential	Yes (Naughton et al. 2014)	No (Studerus et al. 2011b)
Main Route of Administration	Intravenously	Orally

Given such strong evidence of therapeutic efficacy and clinical safety of psilocybin, several clinical studies are currently being under way worldwide, including studies for treatment of depression, anxiety, and addiction (Kupferschmidt 2014) (see **Figure 7** for an overview).

A selection of recent and planned trials with psychedelic drugs

LOCATION	SUBSTANCE	INDICATION	TREATMENT	NUMBER OF PATIENTS	STATUS
Harbor-UCLA Medical Center, Los Angeles, California	Psilocybin	Existential anxiety related to cancer	Two experimental sessions a few weeks apart, one with psilocybin and one with placebo	12	Published in 2011
Private practice in Solothurn, Switzerland	LSD	Anxiety in patients with life-threatening disease	Psychotherapy, including two sessions on LSD	12	Published in 2011
Two independent clinics in Mexico	Ibogaine	Opiate addiction	Ibogaine-assisted psychotherapy	30	Completed in 2012; unpublished
New York University, New York City	Psilocybin	Existential anxiety related to cancer	Nine preparatory psychotherapy sessions; two dosing sessions (one psilocybin, one placebo)	32	Ongoing since 2009
Johns Hopkins University, Baltimore, Maryland	Psilocybin	Existential anxiety related to cancer	Several psychotherapy sessions, including one on psilocybin	44	Ongoing since 2009
Johns Hopkins University	Psilocybin	Nicotine addiction	Several preparatory sessions, then three daylong sessions with psilocybin	15 in an open-label pilot; 80 in a controlled trial against nicotine patch	Pilot completed; paper under review
Imperial College London	Psilocybin	Depression	Psychotherapy with two sessions on oral psilocybin	12 in an open-label pilot; 60 in a controlled trial	Expected to start by the end of 2014
University of Alabama, Birmingham	Psilocybin	Cocaine dependence	Several preparatory sessions, then one daylong session with psilocybin	40	Expected to start by the end of 2014

Source: ClinicalTrials.gov; participating researchers

Figure 7. Selection of recent and planned trials with psychedelic drugs worldwide. A psilocybin treatment study in depressed patients is expected to start by the end of 2014 in England (Kupferschmidt 2014).

7.1 Background and Rationale

Considering preclinical and clinical findings, several researchers have called for continued research into novel medications that mimic ketamine's therapeutic effects by either enhancing glutamate transmission or activating AMPA receptors (Sanacora et al. 2008; Skolnick et al. 2009; Cryan und O'Leary 2010; Duman und Voleti 2012). However, trials with clinically-available glutamate modulators have so far either failed to produce antidepressant effects (e.g., memantine; (Zarate et al. 2006b) or showed inconsistent, time-limited effects (e.g., traxoprodil produced significant antidepressant effects 5 days after infusion, but not at earlier or later assessment points; Preskorn et al. 2008). It is of interest to explore other pharmacological compounds that might have potent antidepressant effects (e.g., via direct stimulation of post-synaptic serotonin receptors) and downstream glutamate activity.

Converging evidence from preclinical, clinical and neuroimaging studies suggests that stimulation of post-synaptic serotonin (5-HT_{2A}) receptors may lead to downstream increases in extracellular glutamate (Vollenweider und Kometer 2010a). Thus, selective 5-HT_{2A} agonists are promising candidates for studying the mechanisms of rapid antidepressant effects because they have both direct serotonergic and indirect glutamatergic effects. The proposed study of psilocybin treatment for major depression will directly test the efficacy of a 5HT_{2A}-agonist in modulating prefrontal-limbic circuitries implicated in the pathophysiology of major depression and producing acute and persistent therapeutic outcomes in patients with major depression.

The safety, tolerability and behavioral potency of the selective 5-HT_{2A} agonist psilocybin has been validated in several independent studies in healthy volunteers and in patients with depression and anxiety related to cancer diagnosis (Griffiths et al. 2006b; Griffiths et al. 2008a; Grob et al. 2011b; Studerus et al. 2011a). The profile of acute and persistent effects (1 and 14-month follow-up) of psilocybin on mood, behaviors, and attitudes suggests antidepressant-like effects. Moreover, psilocybin has lower abuse liability and toxicity than other behaviorally potent glutamate-modulators (e.g., ketamine; Gable 1993) and is generally not associated with long-term perceptual, cognitive, or neurological dysfunction (Studerus et al. 2011a). Thus, psilocybin is a logical focus for study because it is a potent 5-HT_{2A} agonist that has demonstrated safety and tolerability, immediate and persistent mood effects, a greater direct effect on the serotonin system than SSRIs, and an indirect effect on glutamate.

7.2 Investigational Product and Indication

Investigational Product Name: Psilocybin
Chemical Name: 3-(2-(dimethylammonio)ethyl)-1*H*-indol-4-yl hydrogen phosphate
Synonyms: CY-39, Indocybin®, CHEBI:8614
Chemical Family: zwitterionic alkaloid
Molecular Weight: 284.25 g/mol
Chemical Formula: $C_{12}H_{17}N_2O_4P$

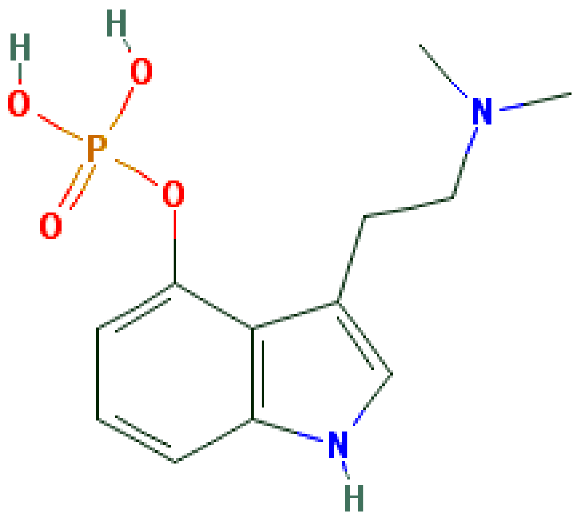

Form: crystalline powder
Color: white to off-white
Odor: odorless
Physical and Chemical Properties:

PH: 5,2 in 50 % aq. ethanol
Melting Point: 210°C-230°C
Solubility: soluble in water and methanol, not soluble in non-polar organic solvents
Stability: stable under standard conditions (tightly closed at 2°C to 8°C)

Psilocybin is the major of two hallucinogenic components of Teonanacatl, the sacred mushroom of Mexico. Psilocybin was first isolated from psilocybe mushrooms by Hofmann in 1957, and later synthesized by him in 1958 (Passie et al. 2002). Soon after, synthetic psilocybin was marketed by Sandoz under the name Indocybin® for basic psychopharmacological and therapeutic clinical research (Studerus et al. 2011b). Psilocybin is a potent serotonin-2A (5-HT_{2A}) agonist that has demonstrated safety and tolerability in both preclinical and clinical studies (Tylš et al. 2014). For details, see the Investigator's Brochure.

7.3 Preclinical Evidence

Psilocybin was marketed by Sandoz under the name Indocybin® for basic pharmacological and clinical research (Studerus et al. 2011b). Psilocybin belongs to a class of drugs referred to as hallucinogens or psychedelics. Specifically, it is a serotonergic hallucinogen, along with other tryptamines such as dimethyltryptamine (DMT), ergolines, such as LSD and phenethylamines, such as mescaline. Currently, research in human and nonhuman animals suggests that serotonergic hallucinogens produce most of their effects as a result of serotonin-2A ($5HT_{2A}$) agonism, with contributions also from agonism at $5HT_{2C}$ and $5HT_{1A}$ receptors (Nichols 2004). Like other members of this class, acute administration of psilocybin induces profound changes in perception, cognition and emotion during 4-6 hours after oral intake. Psilocybin was used in psychiatric and psychological research and as a valuable adjunct in psychodynamic-oriented psychotherapy (Leuner 1966) during the early to mid-1960s up until its scheduling in 1970 in the US. Research into the effects of psilocybin resumed in the mid-

1990s, and it is currently the preferred compound for use in studies of the effects of serotonergic hallucinogens (Baumeister et al. 2014). Since 1990, our and other laboratories have conducted more than 35 preclinical and clinical studies investigating the neurocognitive and emotional effects of psilocybin (Vollenweider et al. 1997; Vollenweider et al. 1998; Vollenweider und Kometer 2010b; Kraehenmann et al. 2014; Carhart-Harris et al. 2012b; Carhart-Harris et al. 2012a; Carter et al. 2005; Hasler et al. 2004; Griffiths et al. 2011). Along this line, we and others (Vollenweider et al. 1998; Vollenweider et al. 1997; Catlow et al. 2013; Bernasconi et al. 2013; Schmidt et al. 2013; Kometer et al. 2012; Kraehenmann et al. 2014; Griffiths et al. 2011; Griffiths et al. 2006a; Griffiths et al. 2008b) have demonstrated that there is strong preclinical evidence that psilocybin may have antidepressant efficacy, as it rapidly reduces neural responses to negative stimuli and enhances prefrontal top-down control over limbic regions which may be important for a fast recovery from depression.

7.4 Clinical Evidence to Date

The majority of clinical studies with psilocybin took place between the mid-1950s and 1960s shortly after its synthesis by the Swiss chemist Albert Hofmann in 1958. Most clinical studies were using Sandoz's Indocybin®, and several thousand scientific papers published by 1965 described positive results in more than 40'000 patients who had taken psychedelics with minimal side effects and a high level of safety (Grinspoon und Bakalar 1981). Up to now, approximately 2000 subjects have received single or repeated doses of psilocybin for treatment of neurotic disorder, alcohol dependence, autism, and anxiety related to terminal cancer. For example, in a recent clinical study, moderate doses (0.215 mg/kg) were given as an anxiolytic and antidepressant in terminally ill cancer patients without clinically significant side effects (Grob et al. 2011a). Case reports and clinical trials have also showed that psilocybin might be effective in the treatment of obsessive-compulsive disorder (OCD), with therapeutic effects lasting up to six months (Moreno et al. 2006). Other ongoing clinical studies may prove that psilocybin stimulates motivation to overcome alcohol and nicotine addiction (ClinicalTrials.gov Identifier NCT01534494, NCT01943994).

7.5 Dose Rationale

A single dose of 0.215mg/kg will be used. This dose is much smaller than the high doses (.3mg/kg and .4mg/kg) recently used in double-blind, placebo-controlled clinical studies, e.g. for treatment of obsessive-compulsive disorder (Moreno et al. 2006) and addiction (ClinicalTrials.gov Identifier NCT01943994, NCT01534494). 0.2mg/kg corresponds to a moderate dose for a psilocybin reaction in humans (Passie et al. 2002). This dose was chosen because it has reliably shown to alter state of consciousness, in particular by altering perception and mood, while being well tolerated in both healthy subjects and psychiatric patients (Passie et al. 2002). In particular, a recent clinical study in terminally ill cancer patients suffering from anxiety and depression showed that a 0.2mg/kg dose of psilocybin was therapeutically effective and well tolerated (Grob et al. 2011a).

7.6 Explanation for Choice of Comparator (or Placebo)

This study investigates the effects of psilocybin in depressed patients. Therefore, pharmaceutically inactive placebo is used in a parallel group randomized double-blind study design. All patients entering the trial will be off psychotropic medications for the duration of the trial, in order to prevent confounding effects by concomitant medication. Because of the relatively short trial duration (8 weeks from study entry till closure visit) and the counselling sessions at each trial visit, adequate and sufficient psychological support is provided for both the active and the comparator group.

7.7 Risk / Benefits

7.7.1 Potential Risks

7.7.1.1 Risks of experimental medications

In this study psilocybin will be used as the putatively active treatment. Psilocybin will be given as a single, moderate dose orally. Psilocybin has been used safely in numerous previous human studies (Tylš et al. 2014). In carefully conducted clinical research settings, with careful screening, preparation and support, the risks of psilocybin administration are low (Tylš et al. 2014; Studerus et al. 2011b; Moreno et al. 2006; Johnson et al. 2008). Classic hallucinogens including psilocybin have the lowest physiological toxicity and lowest dependence potential of all well-known drugs of abuse (Gable 2004). However, there are some rare but potentially adverse reactions that need to be appropriately addressed to maximize safety. In general, adverse reactions are clearly dose-dependent and most of them mostly occur at higher doses than the dose used in this study (Hasler et al. 2004).

- Experiences with hallucinogens may provoke a variety of positive and negative emotional responses following the acute drug experience, which can be unsettling. Anxiety and dysphoria may occur during psilocybin intoxication. These symptoms generally respond well to psychological support and reassurance.
- Increased blood pressure and heart rate during intoxication (mean increase of approximately 20 mm Hg systolic, 12 mm Hg diastolic, mean increase in pulse of approximately 10 bpm) (Griffiths et al. 2006a). This side effect is common, but unlikely to be clinically significant with careful screening for hypertension and cardiovascular disease.
- Transient psychotic symptoms (delusions, paranoia, hallucinations) have been reported during psilocybin intoxication. We are unaware of any reports of persistent psychotic symptoms associated with the use of psilocybin, but such symptoms have been reported following use of LSD (Strassman 1984). Individuals with history of psychosis or vulnerability (e.g., family history of schizophrenia) are thought to be at higher risk for psychotic reactions to hallucinogens and will be excluded, but we cannot rule out a very small risk of such reactions to psilocybin in normal individuals
- Hallucinogen persisting perception disorder (HPPD, “flashbacks”) can be caused by hallucinogen use. The incidence of this disorder is thought to be very low, and associated primarily with use of LSD (Halpern und Pope 2003). To date, however, no cases of HPPD have occurred in volunteers given psilocybin in current research studies (Studerus et al. 2011b). The risk of HPPD occurring after psilocybin

administration can be reduced by screening participants for potential risk factors such as substance dependence and through excluding people reporting HPPD or other significant adverse events after prior use of hallucinogens, as done in this study.

- Headaches are common following psilocybin administration (Studerus et al. 2011b; Johnson et al. 2012). The headaches are not severe, resolve within a day, and appear to respond to over-the-counter pain medications (Johnson et al. 2012).
- Psilocybin, like other classic hallucinogens, is an abusable drug. It is theoretically possible that a participant could develop a pattern of psilocybin misuse due to his/her positive experience in the trial, although this has not been reported in previous studies in which psilocybin or other classic hallucinogens have been administered to humans (Studerus et al. 2011b). Psilocybin has the lowest dependence potential of all well-known drugs of abuse (Gable 2004).

There is considerable clinical experience with psilocybin over more than 50 years, including its use in randomized controlled trials with terminally ill patients suffering from depression and anxiety due to cancer (Tylš et al. 2014). Few safety issues were noted in all these studies.

7.7.1.2 Risks of remaining unmedicated during the study phase

Participants remain unmedicated during the study (3 weeks). This bears the risk of worsening of the condition (depression) and subjective well-being, and an increase of associated risks (e.g. suicidality). However, given that only mildly or moderately, but not severely, depressed patients are included in the study, and given that patients are still receiving psychotherapeutic treatment and are under close supervision, the risks may be minimized.

7.7.1.3 Risks of rescue medications

Use of these medications is expected to occur uncommonly if at all in this study. Acute adverse reactions to psilocybin initially will be managed by increasing psychological support (“talking down”). Rescue medication will be available for onetime administration if needed at all:

- Adalat retard® (nifedipine) 1x 10mg administered orally for hypertension. Common side effects include headache, constipation, vasodilatation, and edema
- Valium® (diazepam) 1x 5-10mg administered orally for severe anxiety. The most common effects are sedation, dizziness, weakness, and unsteadiness. Rare but serious adverse events that could occur with a single administration include respiratory depression, apnea, and anaphylaxis.
- Zyprexa® (olanzapine) 1x 5-10 mg administered orally for psychotic symptoms (delusions, hallucinations, disorganized behavior) that pose a significant danger. Common side effects are somnolence, dizziness, hypotension, akathisia, extrapyramidal symptoms, dry mouth, tremor, nausea, and vomiting. Rare but serious side effects include seizures, neuroleptic malignant syndrome and tardive dyskinesia (the latter two are extremely unlikely with one or two small doses)

7.7.1.4 Risks of blood draws

For most people, needle punctures for blood draws do not cause any serious problems. However, they may cause bleeding, bruising, discomfort, infections and/or pain at the needle site, or dizziness.

7.7.1.5 Risks of assessment procedures

There are no known psychological risks associated with the questionnaires used in the study, all of which have been used extensively in clinical populations. It is possible that discussion of biographical history, significant life events, relationship issues, substance use, and psychiatric symptoms may cause emotional discomfort in some participants. To minimize such discomfort, the following steps will be taken. The consent form will fully inform the participants about the nature of the information to be disclosed in the protocol, and the participants will be informed in the consent form that they can refuse to answer any questions or withdraw from the study at any time. Participants will be informed that all information is confidential. One of the investigators of the project will be available to meet with any participant who becomes distressed about any aspect of the protocol and wishes to discuss this.

7.7.1.6 Risks of functional Magnetic Resonance Investigation (fMRI)

The fMRI investigation is a modern, multi-tested and established non-invasive technique which uses specifically designed equipment. According to current knowledge, the fMRI investigation does not bear any health risks. Although the fMRI investigation does not expose subjects to radiation, metal parts or electronic implants in the body may bear a safety risk because of the electromagnetic field in the scanner and are therefore contraindicated for this investigation (an MRI safety screening will be performed before each scanning session). Scanners with 3.0Tesla field strength are routinely being used in hospitals for many years. To date, no adverse health effects have occurred. Still, subjects should avoid abrupt movements whilst lying in the scanner because this could temporarily induce dizziness or metallic taste in the mouth. Occasionally, subjects may briefly see light flashes (“phosphenes”). For the applied radio waves, limit values similar to mobile phones are applied and are strictly adhered to. Thereby, potential heating of the body is avoided. To protect against knocking noise, all subjects will use appropriate ear protection. During scanning, subjects can communicate with the investigator via intercom at any time. Although no side effects for unborn children have occurred, no fMRI investigation should be performed at healthy, pregnant subjects for research purposes. Therefore, a pregnancy test will be done in female subjects prior to study inclusion. All participants will be informed about possible risks.

7.7.1.7 Risks to confidentiality

Medical records, the investigator’s study related files and correspondence, and the informed consent documentation which identify subjects may be inspected by the competent authority or CEC, respectively for quality assurance purposes. Because of the need to release information to these parties, absolute confidentiality cannot be guaranteed. The results of this research project may be presented at meetings or in publications. However, the identity of individual subjects will not be disclosed in those presentations.

7.7.2 Mitigation of risks

7.7.2.1 Management of risks due to experimental medications

Risks of hallucinogen administration are thought to be minimized by a) careful selection of participants, b) extensive preparation c) presence of qualified study personnel during the psilocybin sessions, d) a safe and reassuring physical environment, and e) follow-up for possible residual adverse effects. These preventive measures are incorporated into our protocol as follows (see also Section 8.6):

a) Participant selection: exclusion criteria are designed to exclude patients who would be at elevated risk for adverse events due to psilocybin. These criteria are listed in Section 7.1., and include both medical exclusions and psychiatric exclusions for serious psychopathology, history of violent or suicidal behavior, and family history of psychosis or suicide.
b) Participant preparation: in addition to the information and discussion provided in the informed consent process during the initial screening visit, participants' will be psychologically assessed and prepared by a trained clinician who is a physician or psychologist. Preparation will include 1) open-ended questions to establish rapport, learn about the participant's history, belief system, and values, assess motivation and expectations for the study, and discuss any previous experience with hallucinogens; 2) detailed information about the physiological and psychological effects of psilocybin; 3) emphasis that the purpose of the psilocybin sessions is to help overcome depressive symptomatology via psilocybin's pharmacological and psychological effects, and discussion of how this could work; 4) advice as to how to deal with dysphoric reactions to psilocybin, should they occur; 5) identification of any personally meaningful items that the participant will bring to the session (e.g., photographs, images, objects of personal or religious significance); and 6) discussion of ground rules for the session, including adherence to the protocol (compliance with dietary restrictions, e.g. alcohol, caffeinated drinks, and other psychoactive substances; effective contraception; restricted use of concomitant medication; transport and care by significant others after psilocybin session).
c) Because this study involves use of psilocybin in a clinical population as part of an intervention intended to be therapeutic, the study therapists, who is a physician or psychologist, will serve as the monitor who will attend and interact with the patient during the psilocybin session.
d) The psilocybin session will take place on-site in an environment that is appropriate for hallucinogen administration sessions. To the extent possible, the room used for the session will be specially prepared for the session to provide a warm, quiet and home-like rather than a stark clinical quality because of the large influence that setting can have on the subjective effects of hallucinogens. Patients will be allowed to lie on a couch or to move freely. Interaction with the patients will be supportive and non-directional. To establish a quiet, relaxing, introspective ambience, no cell phones, laptops, or other electronic devices will be allowed during the session.
e) During treatment sessions, study personnel will be responsible for monitoring participants for possible clinical deterioration or other problems, and for implementing appropriate courses of action.
f) Follow-up sessions after the psilocybin session consist of the following elements: 1) writing of a protocol of subjective experiences by the patients (whenever possible at the psilocybin treatment day); the protocols will be discussed with the

patient to foster integration of the experiences into their lives, to promote insight into depression symptomatology, and to induce attitudinal reorientation; 2) open-ended inquiry concerning the psilocybin session (and what has happened since); 3) invitation to reflect on the experience; discussion of what has changed as a result of the session; 5) discussion of how the session has affected the participant's symptoms; and 6) assessment of mental status and potential adverse effects, including suicidality.

g) An online survey six weeks, one and three months after the investigation will ensure monitoring of subtle changes in symptomology and the subjective need for therapeutic interventions over a longer time-course.

7.7.2.2 Minimization of risk to confidentiality

Confidentiality of research material will be ensured by storing the research materials in locked cabinets. Material will be available only to project staff, and only as needed. All project staff will be thoroughly trained in issues relating to confidentiality. Participants will be identified in case report forms (CRFs) by an identification code.

7.7.3 Potential Benefits

7.7.3.1 Potential Benefits to Participants

Participants may or may not experience clinical benefit from this study. Aspects of study participation likely to be beneficial include free medical and psychiatric evaluations, the attention and support of participating in a clinical trial, and a course of psychological counseling for major depression.

7.7.3.2 Risk Benefit Assessment

Risks to individual participants appear to be balanced by the likely benefits of study participation, and outweighed by the potential benefits to others.

7.7.3.3 Importance of the Knowledge to be Gained

There is an urgent need to develop more effective methods to help people who suffer from major depression. The knowledge gained through this study could point the way to an entirely new treatment for major depression. As indicated above, these potential benefits (in addition to the potential benefits to individual participants) appear to justify the risks to individual participants.

7.8 Justification of Choice of Study Population

Because this study investigates whether psilocybin has therapeutic efficacy in patients diagnosed with major depression, the choice of study population is restricted to this clinical population and cannot be obtained from healthy volunteers without compromising study results. Only patients capable of giving informed consent will be included.

8 STUDY OBJECTIVES

8.1 Overall Objective

The overall purpose of this study is to test the efficacy of the selective 5-HT_{2A} agonist psilocybin compared to placebo in adults with major depression.

About 30-50% of patients fail to respond fully to treatment with available antidepressant medication, and as many as 10-30% remain resistant to these and other treatment options. Therefore, it is of interest to explore novel pharmacological compounds that might have potent antidepressant effects (e.g., via direct stimulation of post-synaptic serotonin receptors) and downstream glutamate/ BDNF activity. Converging evidence from preclinical, clinical and neuroimaging studies suggests that stimulation of post-synaptic serotonin (5-HT_{2A}) receptors may lead to downstream increases in extracellular glutamate and BDNF (Vollenweider und Kometer 2010a). Thus, selective 5-HT_{2A} agonists are promising candidates for studying the mechanisms of rapid antidepressant effects because they have both direct serotonergic and indirect glutamatergic effects. The proposed study of psilocybin treatment for major depression will directly test the efficacy of a 5HT_{2A}-agonist in modulating prefrontal-limbic circuitries implicated in the pathophysiology of major depression and producing acute and persistent therapeutic outcomes in patients with major depression.

The safety, tolerability and behavioral potency of the selective 5-HT_{2A} agonist psilocybin has been validated in several independent studies in healthy volunteers and in patients with depression and anxiety related to cancer diagnosis (Griffiths et al. 2006b; Griffiths et al. 2008a; Grob et al. 2011b; Studerus et al. 2011a). The profile of acute and persistent effects (1 and 14-month follow-up) of psilocybin on mood, behaviors, and attitudes suggests antidepressant-like effects. Moreover, psilocybin has lower abuse liability and toxicity than other behaviorally potent glutamate-modulators (e.g., ketamine; Gable 1993) and is generally not associated with long-term perceptual, cognitive, or neurological dysfunction (Studerus et al. 2011a). Thus, psilocybin is a logical focus for study because it is a potent 5-HT_{2A} agonist that has demonstrated safety and tolerability, immediate and persistent mood effects, a greater direct effect on the serotonin system than SSRIs, and an indirect effect on glutamate/ BDNF.

8.2 Primary Objective

The study seeks primarily to determine effects of a moderate dose of psilocybin (0.215mg/kg) compared to placebo on clinical depression severity in depressed patients. <u>Hypothesis 1: Psilocybin will be well-tolerated, and will produce rapid (within hours) and sustained (>7 days post-treatment) antidepressant effects in depressed patients.</u> Antidepressant effects will primarily be measured by the Montgomery Asberg Depression Scale (MADRS).

8.3 Secondary Objectives

The study further seeks to characterize the effects of psilocybin compared to placebo on depression symptom profile. In particular, it is of interest to determine the symptom-profile in depression which responds well to psilocybin treatment. Hypothesis 2: Given its profound acute effects on subjective experiencing and emotions, psilocybin will particularly have positive effects on anhedonia, anxiety, rumination, hopelessness, coping with stress and the subjective need for therapeutic interventions. These symptom clusters will be measured by the Snaith-Hamilton-Pleasure Scale, the Hamilton Anxiety Scale, the Rumination-Reflection Questionnaire, Emotion Regulation Questionnaire, the Hopelessness Scale, Coping Questionnaire and the Therapy-Questionnaire.

In addition, the study aims at characterizing the effects of psilocybin compared to placebo on neurobehavioral and brain functioning markers of depression. Hypothesis 3: Psilocybin will improve neurocognitive measures of depression severity (Multifaceted Empathy Task), decrease resting-state functional connectivity, increase task-related BOLD fMRI signals in the prefrontal cortex, and decrease task-related BOLD fMRI signals in the amygdala.

Finally, the study seeks to determine the effects of psilocybin compared to placebo on a neurotrophic marker of antidepressant efficacy (BDNF). Hypothesis 4: Psilocybin will increase blood/ serum BDNF levels in relation to a decrease of MADRS depression severity.

8.4 Safety Objectives

Although this study is not a systematic investigation of safety, this study will also clinically assess safety and tolerability of psilocybin treatment in depressed patients. In this study psilocybin will be used as the active treatment. Psilocybin will be given as a single, moderate dose orally. Psilocybin has been used safely in numerous previous human studies (Tylš et al. 2014). Recent clinical studies using psilocybin have shown that the risks of psilocybin administration are low (Tylš et al. 2014; Studerus et al. 2011b; Moreno et al. 2006; Johnson et al. 2008). The following safety endpoints will be used at each visit: occurrence of adverse events, psychological well-being, suicidality, vital signs, body weight, and use of concomitant medication.

9 STUDY OUTCOMES

9.1 Primary Outcome

The study seeks primarily to determine whether psilocybin compared to placebo will produce rapid and sustained antidepressant effects in depressed patients. The primary endpoint will be depression severity, as measured by the score in the **Montgomery Asberg Depression Scale (MADRS)** at screening, at baseline, and following psilocybin administration (2, 8, and 14 days post-treatment). The primary efficacy value will be the

change from baseline in MADRS total score. The clinician-rated MADRS is one of the most commonly used symptom severity scales to evaluate the efficacy of antidepressant treatment (Zimmerman et al. 2004; Schmidtke et al. 1988), and has been used in recent clinical trials of ketamine efficacy in depression (Nugent et al. 2014; Carlson et al. 2013). The MADRS has high inter-rater reliability and bears the advantage of greater sensitivity to change than the Hamilton Depression Rating Scale. Treatment response will be defined as total score improvement of at least 50% from baseline on the MADRS. A MADRS score ≤10 will be prospectively determined definitions of depression remission (Hawley et al. 2002).

In addition to the MADRS, treatment outcome will also be rated using the standard the **Beck Depression Inventory (BDI)** (Hautzinger et al. 2006), and the **Clinical Global Impressions Scale (CGI)** (Guy 1976) at screening, at preclinical visit, at baseline, and following psilocybin administration (2, 8, and 14 days post-treatment). In addition, the BDI will be administered six weeks, three and six months post-treatment in order to capture long-term effects on depressive symptomology and the subjective need for therapeutic interventions.

9.2 Secondary Outcomes

The study further seeks to characterize the effects of psilocybin compared to placebo on <u>depression symptom profiles</u>. The effects of psilocybin on these secondary endpoints will be measured by the Snaith-Hamilton-Pleasure Scale, the Hamilton Anxiety Scale, the Rumination-Reflection Questionnaire, Emotion Regulation Questionnaire, and the Hopelessness Scale at Preclinical Visit, at baseline, and following psilocybin administration (2, 14, 42, 86 and 168 days post-treatment):

a) **The Snaith-Hamilton-Pleasure Scale (SHAPS)** is a 14-item self-report scale evaluating anhedonia, i.e. the inability to experience pleasure or related reduction of ability to react to pleasurable stimuli, which has been considered to be one of the key features for major depression (Franz et al. 1998).
b) **The Hamilton Anxiety Scale (HAMA)** is a 14-item clinician-rated scale evaluating anxiety states and changes (HAMILTON 1959).
c) **The Rumination-Reflection Questionnaire (RSQ)** is a 25-item self-report scale evaluating rumination and reflection, i.e. repeated passive thoughts about negative emotions. Ruminative behavior in depression may prolong and intensify depressed mood and maintain a current depressive episode (Nolen-Hoeksema 2000).
d) **The Hopelessness Scale (HS-Krampen)** is a 20-item self-report scale evaluating negative expectations about oneself, the environment, and the future life (Krampen 1994).
e) **The Emotion Regulation Questionnaire (ERQ)** is a 10-item self-report scale that differentiates between suppression and reappraisal as main strategies regarding the regulation of emotions.
f) **Therapy-Questionnaire (TQ)** is a 9-item self-report scale that aims to capture the subjective need and effective utilization of therapeutic services.
g) **The Stress Questionnaire (CQ)** is 4-item self-report scale that measures potential stressors, the burden it elicits and the progress done regarding this issue. It is adapted from the Stress & Coping Inventory (Satow, 2012).

In addition, the study aims at characterizing the effects of psilocybin compared to placebo on neurocognitive and brain functioning markers of depression at baseline, and following psilocybin administration (2, 8 and 14 days post-treatment):

a) The Multifaceted Empathy Task (MET) is a PC-assisted test that assesses neurocognitive and emotional aspects related to empathy. It consists of 40 photorealistic stimuli showing people in different emotionally charged situations (20 positive, 20 negative). Each picture is presented 3 times with a different question to assess the 3 different components of empathy. Cognitive empathy is operationalized by the question “What is this person feeling?” and participants have to identify the correct mental state from a list of 4 choices. Explicit emotional empathy is operationalized by the question “How concerned are you for this person” (negative valence pictures) and “How happy are you for this person” (positive valence pictures) with a 9-point Likert scale (1=not at all; 9=very much), respectively. To allow for the measurement of emotional empathy while reducing subjects’ tendencies to give socially desirable answers, an implicit emotional empathy condition was also included, which is operationalized by the question “How calm/aroused does this picture make you feel?” with a 9-point Likert scale (1=very calm; 9=very aroused).

b) **Functional Magnetic Resonance Imaging (FMRI)** will be used to measure BOLD signal changes at baseline, and following psilocybin administration (2 and 14 days post-treatment).
    a. **Resting State FMRI.** The resting state is a task free procedure. Participants are instructed to observe a fixation cross on the screen inside the scanner and to rest, without engaging in any specific task or mental activity. Under such “task-free” conditions, it is assumed that BOLD changes are caused by spontaneous and intrinsic neuronal activity within a network of brain regions (Fox und Raichle 2007). Accumulating evidence indicates that resting-state brain activity is consistently disrupted in mood disorders and might even be a core feature of major depression (Northoff et al. 2011). Previous studies revealed elevated resting state functional connectivity in depression (Sheline et al. 2010) which is normalized following antidepressant treatment with serotonergic medication. Previous studies (Carhart-Harris et al. 2012b) have shown that psilocybin may decrease resting state functional connectivity in healthy volunteers. The measurement will take about 10 minutes.
    b. **FMRI Amygdala Reactivity Paradigm.** The amygdala is a key structure in the serotonergic neurocircuitry of emotion processing. Amygdala hyperactivity in response to negative stimuli and a relation between amygdala activity and negative mood states have consistently been found in depressed patients. Further, it has been found that amygdala hyperactivity in patients with depression decreases after antidepressant treatment with SSRIs and this was associated with mood changes toward positive states. Using BOLD FMRI, we (Kraehenmann et al. 2014) have recently shown that psilocybin decreased amygdala reactivity during negative-emotion processing, and that this was associated with a parallel increase of positive mood state in healthy volunteers. Task: Subjects a required to select one of the two IAPS (International Affective Picture System) pictures at the bottom of a stimuli triplet that matched the target picture at the top of the triplet. Selection will be indicated by pressing one of two buttons on a magnetic resonance (MR)-compatible response device with the dominant hand. A shape discrimination task will be performed as a

sensorimotor control and baseline task. This requires matching of geometric shapes (circles, ovals, and rectangles) analogous to the picture discrimination task. Both tasks will be shown as alternating blocks without intermittent pauses. The measurement will take about 10 minutes.

c. **FMRI Self Referential Processing Paradigm.** A disturbed sense of self is a core feature of depression. The medial prefrontal cortex, which has a central role in self-appraisal processes, is often implicated in the illness, and was found to have a « hyperregulatory » influence on the rest of the network when measuring depressed patients. In order to quantify this processes, an fMRI task with three different experimental condition was developed by Davey et al. (2017). In the self-appraisal condition, participants will be presented with a personality adjective and asked whether or not the word describes them. Words are drawn from a frequently used list of personality adjectives. In the external attention condition, participants viewed eight blocks of sixwords, also presented for 5 seconds each, and responded to the question “Does this word have four or more vowels?”. Selection will be indicated by pressing one of two buttons on a magnetic resonance (MR)-compatible response device with the dominant hand. In order to infer the causal architecture of a network of neural regions, a Dynamic Causal Modeling approach will be used to calculate the regulatory influence of the medial prefrontal cortex.

c) **Brain Derived Neurotrophic Factor (BDNF).** Rapid upregulation of the neuroplasticity marker BDNF is implicated as a critical component of the antidepressant mechanism of ketamine and other antidepressants (Haile et al. 2014; Mikoteit et al. 2014). BDNF is a neurotrophin important in facilitating and supporting certain neuronal populations during development and mediating synaptic plasticity associated with learning and memory (Poo 2001). It has been shown that successful pharmacotherapy in patients with depression is associated with an increase in blood BDNF levels and is negatively correlated with MADRS depression scores (Haile et al. 2014). For BDNF sampling, blood (5ml from antecubital vein) will be collected at baseline, and following psilocybin administration (0, 2, and 7 days post-treatment), always at the same time in the afternoon. Two probes per sampling will be collected from each patient into Vacutainer tubes and the tubes will be appropriately labeled. After 30 minutes of clotting time, the whole blood will be centrifugated at 1000xg for 30minutes to separate and collect the serum. Aliquots will be kept at -80°C until assaying. Serum BDNF levels will be assessed with an enzyme-linked immunoabsorbent assay (ELISA) kit in duplicates at the Neurobiology Laboratory for Brain Aging and Mental Health, Psychiatric University Clinic, University of Basel, Switzerland.

9.3 Safety Outcomes

In this study, the efficacy of psilocybin treatment for major depression will be systematically assessed. Although this study is not a systematic investigation of safety, this study will also assess safety and tolerability of psilocybin treatment in depressed patients. The following safety endpoints will be used at each visit: occurrence of adverse events (AEs), psychological and physical well-being, suicidality, vital signs, body weight, and use of concomitant medication. All observed or volunteered abnormal safety

endpoints will be recorded in the CRF. The investigator will promptly review documented AEs or other abnormal safety endpoints to determine if

- the abnormal abnormal safety endpoint should be classified as an AE,
- if there is a reasonable possibility that the AE was caused by the investigational drug, and
- if the AE meets the criteria for a serious AE (SAE).

The Investigator is responsible for SAE reporting to the CEC.

10 STUDY DESIGN AND COURSE OF STUDY

10.1 General Study Design and Justification of the Design

2 x 30 patients diagnosed with major depression (30 participants for each study arm) will be investigated in a single-centre, double-blind, placebo-controlled, parallel-group design clinical trial contrasting the acute and persisting effects of psilocybin to those of placebo in major depression. Participants will be randomly assigned to psilocybin or placebo group with a 1:1 allocation ration as per a computer generated randomisation. In the psilocybin treatment arm, participants will receive a single moderate dose of psilocybin (0.215mg/kg). The drug will be administered during an 8 hour sessions in an outpatient setting under close medical and psychiatric monitoring. The drug administration sessions will occur in the context of an unstructured psychological counseling intervention, with the addition of standardized preparation before and follow-up after the psilocybin administration sessions. Extensive screening and baseline assessment will be completed, including thorough safety screening and assessment of participant characteristics that could potentially moderate treatment response. Clinical outcomes and changes in several potential mediators of treatment effect, including neurocognition (MET), emotion regulation (FMRI), and treatment response markers (BDNF), will be measured at baseline and following treatment. The study procedures comprise a total of 7 visits during a total duration of 4-8 weeks and includes the following visits: screening visit (ca. 5h), pre-investigation visit (ca. 4h), baseline measures visit (ca. 6h), treatment visit (ca. 8h), post-treatment measures visit (ca. 6h), follow-up visit (ca. 3h), follow-up measures & closure visit (ca 7h). The total amount of time needed for the whole study is about 40 hours. Three online surveys after six weeks, as well as one and three months, respectively, will complement the clinical assessment by providing the opportunity to capture long-term changes in symptomology and the subjective need for therapeutic interventions.

10.2 Methods of Minimising Bias

The study will be performed in a placebo-controlled, parallel-group randomized (random allocation of treatment arm) and double-blind manner.

10.2.1 Randomisation

The resident pharmacy who will have no other role in the study and will not communicate with the participants and the investigators about the study procedures will randomly

allocate the participants to either the psilocybin or the placebo treatment arm with a 1:1 allocation ratio as per a computer generated randomisation schedule.

10.2.2 Blinding Procedures

All individual-related data will be encrypted. The subject code will be generated by the investigator at study inclusion and will be individually allocated. Psilocybin and placebo will be administered in gelatin capsules of identical appearance. A lab member who will have no other role in the study and will not communicate with the subjects and the investigators about the study and keep a list of the randomization codes, which will allow rapid de-codification in case of emergency. An emergency code break will be available to the investigators (see 6.3).

10.2.3 Other Methods of Minimising Bias

All questionnaires and MR-sequences applied in this study are widely used and well-validated measures.

10.3 Unblinding Procedures (Code break)

In circumstances under which unblinding is permissible, e.g. for SAEs or in case of a medical emergency, an Emergency Code Break in sealed envelopes will be available to the investigators. This Code Break should be opened only in emergency situations when the identity of the investigational product must be known by the investigator in order to provide appropriate medical treatment.

11 STUDY POPULATION

Male or female outpatients (aged 18-60 years) who meet DSM-IV (American Psychiatric Association 2011) criteria for major depressive disorder (as determined at screening by the Mini-International Neuropsychiatric Interview) (Sheehan et al. 1998) are eligible for the study. Patients are required to have been experiencing a major depressive episode for ≥ 4 weeks prior to study entry and have a score of ≥ 10 on the Montgomery-Asberg Depression Rating Scale (MADRS) (Montgomery und Asberg 1979; Schmidtke et al. 1988) at both screening and baseline visits. A total number of 60 participants (30 for each treatment arm) will be enrolled by the investigator. Based on the completion date of the study, an expected enrolment goal will be 3-4 participants per month. If the enrolment goals are not met, recruitment rate will be increased via intensification of recruitment strategies.

11.1 Eligibility Criteria

11.1.1 Inclusion Criteria

Participants fulfilling all of the following inclusion criteria are eligible for the study:

- Capable of giving informed consent

- Informed consent as documented by signature
- Male and female in- and outpatients 18 years to 60 years of age
- Right-handedness
- DSM-IV-diagnosis of mild or moderate major depressive episode without psychotic features (based on clinical assessment and confirmed by the SCID Interview)
- Score of ≥ 10 and ≤40 on the Montgomery-Asberg Depression Rating Scale (MADRS) at both screening and baseline visits.
- Drug free from any psychotropic medication for at least two weeks (or five weeks for fluoxetine) before enrolling in the study
- Judged clinically not to be a serious suicide risk
- Good physical health with no unstable medical conditions, as determined by medical history, physical examination, routine blood labs, electrocardiogram, urineanalysis, and urine toxicology
- Normal level of language comprehension and German or Swiss-German as first language
- Willing to refrain from drinking alcohol the day before testing days, from drinking alcohol and caffeinated drinks during the testing days and from consuming psychoactive substances 2 weeks before enrolling in the study and for the remainder of the study
- Women of childbearing potential must be using an effective, established method of contraception for the entire study duration, such as oral, injectable, or implantable contraceptives, or intrauterine contraceptive devices. Note: female participants who are surgically sterilised / hysterectomised or post-menopausal for longer than 2 years are not considered as being of child bearing potential.
- Have a family member or friend who can pick them up and stay with them overnight after the psilocybin administration sessions (driving is forbidden at drug treatment days)

11.1.2 Exclusion Criteria

The presence of any one of the following exclusion criteria will lead to exclusion of the participant:

- Lifetime history of bipolar disorder (I, II, not otherwise specified)
- Lifetime history of schizophrenia, schizoaffective disorder, or psychosis not otherwise specified
- History of DSM-IV drug or alcohol dependence or abuse (except for caffeine or nicotine) within three months prior to enrollment
- Comorbid Axis I anxiety disorder diagnoses will be permitted if they do not require current treatment
- Family history of schizophrenia or schizoaffective disorder, or bipolar disorder type 1 (first or second degree relatives)

- Lifetime history of hallucinogen use on more than 10 occasions
- Getting psychotherapeutic or psychological treatment from third parties during the study is forbidden
- Abnormal electrocardiogram
- Any unstable illness as determined by history or laboratory tests
- BMI <17 or >35
- Uncorrected hypo- or hyperthyroidism
- Women who are pregnant or breast feeding, or have the intention to become pregnant during the course of the study
- Contraindications to magnetic resonance imaging (MRI safety form)
- During the study, new use or dose changes of already existing concomitant medication without prior informing the investigators is forbidden
- Allergy, hypersensitivity, or other adverse reaction to previous use of psilocybin or other hallucinogens
- High risk of adverse emotional or behavioral reaction based on investigator's clinical evaluation (e.g., evidence of serious personality disorder, antisocial behavior, serious current stressors, lack of meaningful social support)
- Participation in another study with investigational drug within the 30 days preceding and during the present study

11.2 Recruitment, Screening, and Payment

Participants will be recruited from referrals from local inpatient and outpatient psychiatric units or through advertisements placed in the local or national newspapers; flyers placed in community locations where this is permitted; the Internet (e.g. www.depressionen.ch; www.depri.ch; zadz.ch/forschung/; www.diskussionsforum-depression.de/forum-depression; www.forum-depressionen.de; www.hilferuf.de/forum; www.beobachter.ch/foren) and local and national referrals from physicians. Those who are interested will be pre-screened using IRB-approved pre-screening forms including basic demographic data and questions related to inclusion and exclusion criteria. Those who pass pre-screening will be scheduled for a screening visit. Details of the prescreening and screening process are described in section 13.3.

Prior to every visit at the Psychiatric University Clinic Zurich, we will phone the patients and ask them about their risk of COVID-19 exposure. This will include infection relevant symptoms, body temperature and contact with people with COVID-19 or people in quarantine. If there is a risk of COVID-19 infection, the visit will be postponed for ten days. These measures reflect the guidelines from the direction of the Psychiatric University Clinic Zurich. They will be updated regularly according to the local situation of the pandemic and will strictly follow the current guidelines of the Bundesamt für Gesundheit (BAG).

Participants will not be paid for study participation. However, transport costs to and from the study visits and food/drinking in the on-site cafeteria during visits will be reimbursed if a receipt is provided.

11.3 Assignment to Study Groups

Participants will be randomly assigned to psilocybin or placebo group with a 1:1 allocation ration as per a computer generated randomisation schedule. Each participant will be assigned a randomisation code which corresponds to numbers printed on the kit containing the study drug, and which will be matched to a confidential treatment allocation number by a lab member not involved in the study to assign participants either to psilocybin or to placebo. Allocation concealment will be ensured, as the randomisation codes will not be released until the patient has been recruited into the trial, which takes place after all baseline measurements have been completed. The participants, investigators, and sponsor personnel will be masked to treatment allocation. Drug kits will be prepared and labeled with the randomisation code by the resident pharmacy. The psilocybin and placebo capsules will be similar in number, colour, shape, size, texture, and taste. To guarantee masking throughout the study, the treatment allocation number will be kept in a confidential key-locked place on-site and will only be accessible by the resident pharmacy. In case of a medical emergency the treatment allocation number can be accessed by the investigators (see 6.3). Access to the treatment allocation number will be controlled and documented.

11.4 Criteria for Withdrawal/ Discontinuation of Participants

The study may be discontinued by the participant at any time without disclosure of reasons. The study may also be discontinued by the investigator if the participant does not comply with the study-specific agreements, if further study participation would bear a risk to the health of the participant, or in case of pregnancy. If the study is discontinued, drop-out date and reasons must be documented in the case report form (CRF). Participants who discontinue the study will be replaced until the determined sample size of participants completing the whole study is reached. Please see section 9.2.5 for description of follow-up procedures.

12 STUDY INTERVENTION

12.1 Identity of Investigational Product(s)

Participants will be randomly allocated to one of two study arms and will receive either a single dose of active drug (psilocybin) or placebo (100% mannitol) at visit no. 4 (see section 9.1). No other treatments or diagnostic agents will be administered during the study. Psilocybin and placebo will be administered orally as white, oval-shaped, 2cm long, smooth, tasteless gelatin capsules. The psilocybin and placebo capsules will be similar in number, colour, shape, size, texture, and taste. Psilocybin dosage will be relative to the body weight of the participants at baseline visit (visit no. 3) corresponding to a onetime target dose of 0.215mg/kg body weight po. Psilocybin capsules will contain either 1mg or 5mg psilocybin. For each participant, a combination of a certain number of 1mg and 5mg capsules will be used in order to best approximate the target dose. The smallest number of capsules will be used (e.g. 2 x 5mg rather than 1 x 5mg + 5 x 1mg capsules), and the

absolute dose given should not fall below the target dose (e.g. target dose for a 80 kg participant = 17.2 mg, composite dose given = 3 x 5mg + 3 x 1mg = 18mg). Drug preparation and labeling of drug kits will be done by a lab member not involved in the study.

12.1.1 Experimental Intervention

Pharmaceutically pure psilocybin will be used. Psilocybin will be provided by Compass Pathways Ltd, Wheatsheaf Cottage, Liphook, Hampshire GU30 7EG London, Englandwith the authorization of the Federal Office of Public Health (BAG), Bern. The investigator will pay for the product (no contract). The drug was synthesized by Compass Pathways Ltd, Wheatsheaf Cottage, Liphook, Hampshire GU30 7EG London, England according to Good Manufacturing Practice. Psilocybin from this batch will be prepared as white capsules of identical appearance to the placebo capsules by the Kantonsapotheke Zürich (KAZ), 8006 Zürich.

Treatment A (test drug)

- Generic name: **psilocybin** (4-Phosphoryloxy-N,N-dimethyltryptamine, CY-39, indocybin)
- Source of psilocybin:
  Compass Pathways Ltd Wheatsheaf Cottage Liphook, Hampshire GU30 7EG London, England
  https://compasspathways.com/
- Source of capsules:
  Kantonsapotheke Zürich (KAZ)
  Spöndlistrasse 9
  8006 Zürich
  Tel. +41 (0)44 266 25 40
  Fax +41 (0)44 266 45 46

- Pharmaceutical form: white, oval-shaped, 2cm long, smooth, tasteless gelatin capsules of 1mg and 5mg psilocybin.

12.1.2 Control Intervention

Treatment B (placebo)

- Placebo: 100% mannitol
- Source of capsules and placebo:
  Kantonsapotheke Zürich (KAZ)
  Spöndlistrasse 9
  8006 Zürich
  Tel. +41 (0)44 266 25 40
  Fax +41 (0)44 266 45 46
- Pharmaceutical form: white, oval-shaped, 2cm long, smooth, tasteless gelatin capsules

12.1.3 Packaging, Labelling and Supply (Re-Supply)

The test drug (psilocybin) will be shipped in bulk from Compass Pathways Ltd, England, to the the investigational site, Prof. Franz X. Vollenweider, Psychiatric University Hospital Zurich. The investigational site will then forward the test drug to the Kantonsapotheke Zürich (KAZ), Spöndlistrasse 9, 8006 Zürich, for preparation of the capsules and packaging. There, psilocybin will be packaged in bottles containing 50 capsules of 1mg or 5mg psilocybin, and placebo will be packaged in bottles containing 50 capsules. All capsules will be of identical appearance. The capsules will then be sent back to the investigational site (Psychiatric University Hospital Zurich), where a lab member not involved in the study will prepare and label the participant-specific drug kits. Drug kits will contain participant-specific number of identical capsules (see 8.1) and be labeled according to Good Manufacturing Practice (EUDRALEX Volume 4 – Medicinal Products for Human and Veterinary Use). Labels will be attached to the drug kit (dark tight-closed plastic bags) and will contain the following information:

Psychiatrische Universitätsklinik Zürich

Phase II, randomized, double blind, placebo controlled, parallel group, single center study of psilocybin efficacy in major depression / PSIDEPR-128

0.215mg/kg KG Psilocybin oder Placebo Kapseln

Patienten-Nr.: XXX Randomisierungs-Nr. XXXX

Dosierung: 0.215mg/kg KG einmalig oral einnehmen.

Chargennummer:XXXXXX Verfallsdatum: XX.XX.XXXX

Für Kinder unzugänglich aufbewahren
Bei 2° bis 8° fest verschlossen lichtgeschützt lagern.

Sponsor und Hauptprüfer: Prof. Dr. med. Franz X. Vollenweider, Psychiatrische Universitätsklinik Zürich, Lenggstrasse 31, 8032 Zürich, Tel.: +41 (0)44 384 2604 (direkt) oder +41 (0)44 384 2404 (Sekretariat)

Upon receipt of the test drug, an inventory will be performed and a drug receipt log filled out and signed by the person accepting the shipment. It is important that the designated study staff counts and verifies that the shipment contains all the items noted in the shipment inventory. Any damaged or unusable study drug in a given shipment (active drug or comparator) will be documented in the study files. Active or comparator drugs will be assigned to each participant by the resident pharmacist in the study according to treatment allocation (see 7.3). To implement masking, drug kits will be prepared and labeled by the resident pharmacist who then forwards the drug kits to the investigator. The investigator will then dispense the study drug to the participants. Regular study drug reconciliation will be performed to document drug assigned; drug consumed, and drug remaining. This reconciliation will be logged on the drug accountability form, and signed and dated by the study team. Both empty drug kits (plastic bags with label) and unused study drug will be returned to the resident pharmacist for drug preparation. This will also be logged on the drug accountability form.

12.1.4 Storage Conditions

According to storage requirements of the manufacturer (temperature range 2°-8°, storage within tightly sealed devices), both psilocybin and placebo will be stored in tightly sealed plastic bottles and kept in a locked temperature controlled cooling device in a lockable room with restricted access on-site at 2-8°. The temperature of the cooling device will be regularly documented on a temperature log device.

12.2 Administration of Experimental and Control interventions

12.2.1 Experimental Intervention

In the experimental treatment arm, participants will receive a single dose of psilocybin at visit no. 4 (see section 9.1). No other treatments or diagnostic agents will be administered during the study. Psilocybin will be administered orally as white, oval-shaped, 2cm long, smooth, tasteless gelatin capsules. It has been shown (Hasler et al. 1997) that for oral administration, relative psilocybin dosage is superior to absolute dosage in terms of plasma concentration profiles and their comparability. We therefore prefer to administer doses relative to body weight rather than absolute doses of psilocybin. Psilocybin dose will be 0.215mg/kg body weight po. Although the optimal dose of psilocybin in the treatment of depression is unknown, several arguments suggest that a single moderate dose (0.215 mg/kg body weight po) would be predictive of therapeutic benefit. In our previous psilocybin studies in healthy volunteers, we showed that a low-to-moderate (0.16 mg/kg body weight po) or moderate (0.215 mg/kg body weight po) dose of psilocybin was capable to enhance mood state and emotion processing via a unique neurobiological mechanism involving the amygdala. The amygdala is a crucial target region underlying therapeutic efficacy of psilocybin in depression, because psilocybin may inhibit amygdala reactivity to negative stimuli, which may normalize negative processing biases, eventually leading to remission of depressed mood state (Kraehenmann et al. 2014; Kometer et al. 2012; Vollenweider und Kometer 2010b). Furthermore, there has been evidence from human and animal studies (Vollenweider und Kometer 2010b; Catlow et al. 2013; Baumeister et al. 2014), that especially in the lower dose range, psilocybin may have some potentially similar effects to that of antidepressants, such as selective serotonin reuptake inhibitors (SSRIs), which increase neurogenesis and neurotrophic factors (e.g. BDNF). A previous clinical study of psilocybin in patients with advanced-stage cancer and comorbid depression and anxiety showed that a onetime moderate dose of psilocybin (0.2mg/kg) significantly increased positive mood and decreased depression and anxiety (Grob et al. 2011a). In all previous clinical studies moderate and even high doses of psilocybin were well-tolerated, and none of the volunteers reported persisting adverse effects (Grob et al. 2011a; Moreno et al. 2006; Baumeister et al. 2014).

12.2.2 Control Intervention

In the control treatment arm, participants will receive a single dose of placebo at visit no. 4 (see section 9.1). No other treatments or diagnostic agents will be administered during the study. Placebo will be administered orally as white, oval-shaped, 2cm long, smooth, tasteless gelatin capsules.

12.3 Dose Modifications

This study comprises a onetime application of a single dose. Therefore, no dose modifications are planned.

12.4 Compliance with Study Intervention

This study comprises a onetime application of a single dose of psilocybin, which will be directly dispensed to the participants by the investigators at visit no. 4 (see section 9.1). No other treatments or diagnostic agents will be administered during the study. Therefore, there is no need to monitor compliance with treatment (psilocybin). To exclude current use of psychoactive drugs a urine test will be done at the beginning of visits 1, 3, 5, 7. Furthermore, a urine pregnancy test will be done for all women capable of bearing children.

12.5 Data Collection and Follow-up for Withdrawn Participants

For whatever reason a participant or an investigator decides that a participant should withdraw from the study, the investigator will make efforts to conduct an exit visit to assess the safety and well-being of the participant. The exit visit will include vital signs, safety assessments, and reasons for withdrawal. The exit visit will be performed as soon as possible after the withdrawal. If a participant does not agree to an exit visit, a telephone interview will be performed instead, and the participant will be questioned on physical well-being, safety issues, and reasons for withdrawal. When a participant withdraws from the study, the data collected on the participant to the point of withdrawal remains part of the study database and will not be removed.

12.6 Trial Specific Preventive Measures

Risks are minimized by a) careful selection of participants, b) extensive preparation c) presence of qualified study personnel during the psilocybin sessions, d) a safe and reassuring physical environment, and e) follow-up for possible residual adverse effects. These preventive measures are incorporated into our protocol as follows.

a) Participant selection: exclusion criteria are designed to exclude patients who would be at elevated risk for adverse events due to psilocybin. These criteria are listed in Section 7.1., and include both medical exclusions and psychiatric exclusions for serious psychopathology, history of violent or suicidal behavior, and family history of psychosis or suicide.
b) Participant preparation: in addition to the information and discussion provided in the informed consent process during the initial screening visit, participants' will be psychologically assessed and prepared by a psychiatrist. Preparation will include 1) open-ended questions to establish rapport, learn about the participant's history, belief system, and values, assess motivation and expectations for the study, and discuss any previous experience with hallucinogens; 2) detailed information about the physiological and psychological effects of psilocybin; 3) emphasis that the purpose of the psilocybin sessions is to help overcome depressive

symptomatology via psilocybin's pharmacological and psychological effects, and discussion of how this could work; 4) advice as to how to deal with dysphoric reactions to psilocybin, should they occur; and 5) discussion of ground rules for the session, including adherence to the protocol (compliance with dietary restrictions, e.g. alcohol, caffeinated drinks, and other psychoactive substances; effective contraception; restricted use of concomitant medication; transport and care by significant others after psilocybin session).

c) Because this study involves use of psilocybin in a clinical population as part of an intervention intended to be therapeutic, the study therapists, who is a physician or psychologist, will serve as the monitor who will attend and interact with the patient during the psilocybin session.
d) The psilocybin session will take place on-site in an environment that is appropriate for hallucinogen administration sessions. To the extent possible, the room used for the session will be specially prepared for the session to provide a warm, quiet and home-like rather than a stark clinical quality because of the large influence that setting can have on the subjective effects of hallucinogens. Patients will be allowed to lie on a couch or to move freely. Interaction with the patients will be supportive and non-directional. To establish a quiet, relaxing, introspective ambience, no cell phones, laptops, or other electronic devices will be allowed during the session.
e) During treatment sessions, study personnel will be responsible for monitoring participants for possible clinical deterioration or other problems, and for implementing appropriate courses of action.
f) Follow-up sessions after the psilocybin session consist of the following elements: 1) writing of separate protocols by the patients (whenever possible at the psilocybin treatment day) and the monitor (who will use a tape recorder to record and thereupon write a protocol of the verbal accounts of the patient during the psilocybin session); the protocols will be discussed with the patient to foster integration of the experiences into their lives, to promote insight into depression symptomatology, and to induce attitudinal reorientation; 2) open-ended inquiry concerning the psilocybin session (and what has happened since); 3) invitation to reflect on the experience; 4) discussion of what has changed as a result of the session; 5) discussion of how the session has affected the participant's symptoms; and 6) assessment of mental status and potential adverse effects, including suicidality.
g) An online survey six weeks, as well as one and three months after the investigation will ensure monitoring of subtle changes in symptomology and the subjective need for therapeutic interventions over a longer time-course.

12.7 Concomitant Intervention(s)

Concomitant interventions will be assessed at study entry and during the study in order to prevent confound or undesirable impact on patients and study endpoint measures, respectively. In general, concomitant interventions, either before or during the study, are forbidden. In particular, the following drugs or procedures are forbidden:

a) Psychotropic medication for at least two weeks (or five weeks for fluoxetine) before enrolling in the study

b) New use or dose changes of already existing concomitant medication without prior information of the investigators during the study
c) Drinking alcohol the day before testing days, or drinking alcohol and caffeinated drinks during testing days, or consuming psychoactive substances 2 weeks before and during the study
d) Getting psychotherapeutic or psychological treatment from third parties during the study
e) Participation in another study with investigational drug or psychological intervention within the 30 days preceding and during the present study
f) Any other drugs or procedures which may influence study performance or endpoint measures.

The following drugs or procedures are allowed, but must be discussed with or communicated to the study personnel:

a) Medical drugs or interventions unrelated to depression before or during the study

b) Rescue drugs during the study. Acute adverse reactions to psilocybin initially will be managed by increasing psychological support (“talking down”). Rescue medication will be available for onetime administration if needed to treat: 1) hypertension (Adalat retard® 10mg oral); 2) anxiety (Valium® 5-10 mg oral); or 3) acute psychosis posing a danger to the participant or others (Zyprexa® 5-10 mg oral). Referral for emergency treatment and/or hospitalization will be available on site. A study clinician will be available by pager or phone at all times during study participation. Use of these medications is expected to occur uncommonly if at all in this study.

All concomitant and/or rescue treatment(s) have to be recorded in the eCRF.

12.8 Study Drug Accountability

Active or comparator drugs, which will be provided to the Sponsor-Investigator, will be kept in a secure, limited access storage area under the recommended storage conditions (see Section 8.1.4). Regular study drug reconciliation will be performed to document receipt of test drug; drug assigned; drug consumed; drug remaining; and drug destruction. Drug accountability will be accurately and adequately recorded, including dates, lot number, quantities received/ returned/ missing/ destructed (see Section 8.1.3).

12.9 Return or Destruction of Study Drug

At the completion of the study, there will be a final reconciliation of drug shipped, drug consumed, and drug remaining. This reconciliation will be logged on the drug accountability form, signed and dated. Any discrepancies noted will be investigated, resolved, and documented prior to destruction of unused study drug. Drug destroyed on site will be documented in the study files.

13 STUDY ASSESSMENTS

13.1 Study Flow Chart/ Table of Study Procedures and Assessments

Flow chart of study schedule:

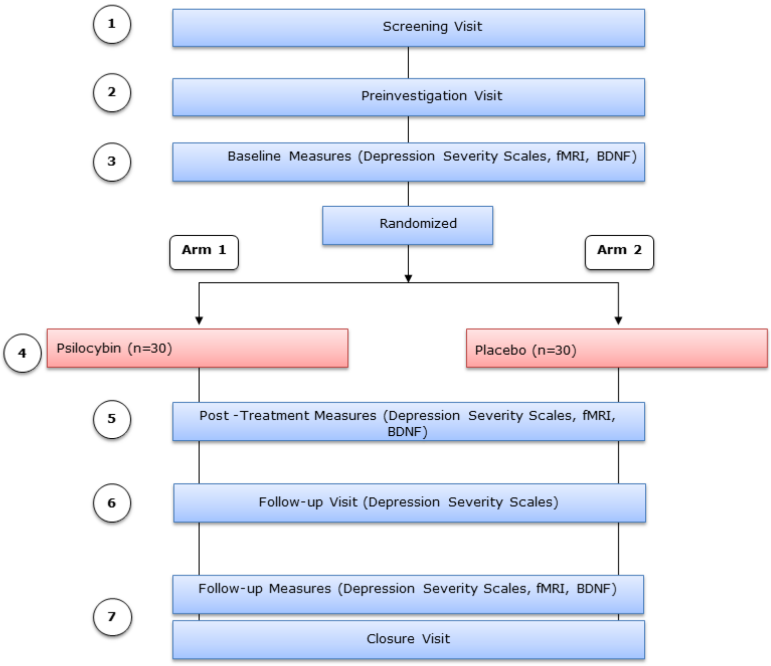

Tabular listing of study schedule:

Study Periods	***Screening/ Preinvestigation Visits***			***Treatment Period***	***Postinvestigation Visits***			***Follow-up surveys***		
Visit	***1***	***2***	***3***	***4***	***5***	***6***	***7***	***8***	***9***	***10***
Time (t)	***t0-4218d (+/37-d)***	***t0-43d (+/-2d)***	***t0-24h (+/-0dh)***	***t0***	***t0+48h (+/-0dh)***	***t0+8d (+/-1d)***	***t0+14d (+/-2d)***	***t0+42d (+/- 5d)***	***t0+84d (+/-5d)***	***t0+168d (+/- 5d)***
Patient Information and Informed Consent	*20'*									

Biographical Anamnesis		*60’*								
Medical/ Psychiatric History, including Family History		*40’*								
Suicidality (C-SSRS)	*15’*	*15’*	*15’*	*15’*	*15’*	*15’*	*15’*			
Symptom Checklist (SCL 90-R)	*20’*							*20’*	*20’*	*20’*
Structured Clinical Interview (SCID I and II)	*50’*									
Frankfurt Self-Concept Scale (FSCS)		*20’*					*20’*			
Verbal IQ (MWT-B)	*10’*									
In- /Exclusion Criteria	*15’*									
MRI Safety Form			*10’*		*10’*		*10’*			
Physical Examination	*20’*									
Echocardiogra m	*20’*									
Vital Signs	*5’*	*5’*	*5’*	*5’*	*5’*	*5’*	*5’*			
Body Weight	*5’*	*5’*	*5’*	*5’*	*5’*	*5’*	*5’*			
Concomitant Medication Form	*5’*	*5’*	*5’*	*5’*	*5’*	*5’*	*5’*	*5’*	*5’*	*5’*
Drug Urine Test	*10’*		*10’*		*10’*		*10’*			
Drug Screening	*10’*		*10’*		*10’*		*10’*	*10’*	*10’*	*10’*

Routine Laboratory Tests[3]	*10'*									
Urine Pregnancy Test[4] *(Women only)*	*10'*									
Randomization			*5'*							
Psychological Counselling Session		*60'*	*60'*	*60'*	*60'*	*60'*	*60'*			
Tape Recordings	*90'*	*60'*	*60'*	*60'*	*60'*	*60'*	*60'*			
Relaxation Exercise		*25'*	*25'*	*25'*	*25'*	*25'*	*25'*			
Résumé written by participants	*60'*									
Protocol of subjective experiencing during drug treatment session, written by participants				*60'*						
Administer Study Medication				*10' (t0)*						
Altered States of Consciousnes s Questionnaire (5D-ASC)				*20' (t0+~6h)*						
Stundenbogen (STEPP, STEPT)		*5'*	*5'*	*5' (t0+~6h)*	*5'*	*5'*	*5'*			

3 Hemoglobin, white blood cell count (WBC), sodium (Na), potassium (K), calcium (Ca), glucose, creatinine, aspartate aminotransferase (AST), alanine aminotransferase (ALT), gamma-glutamyltransferase (GGT), thyroid stimulating hormone (TSH)

4 human corionic gonadotropin (hCG)

FMRI tasks			*60’*		*60’*		*60’*			
MET			*30’*		*30’*	*30’*	*30’*			
Blood & Serum Brain-Derived Neurotrophic Factor (BDNF) levels			*5’*	*5’*	*5’*					
Clinical Global Impressions (CGI)	*10’*		*10’*	*10’ (t0+~6h)*	*10’*	*10’*	*10’*			
Montgomery-Asberg Depression Scale (MADRS)	*15’*	*15’*	*15’*	*15’ (t0+~6h)*	*15’*	*15’*	*15’*			
Emotion-Regulation Questionnaire			*15’*		*15’*		*15’*			
Beck Depression Inventory (BDI)	*15’*	*15’*	*15’*	*15’ (t0+~6h)*	*15’*	*15’*	*15’*	*15’*	*15’*	*15’*
Snaith-Hamilton-Pleasure Scale (SHAPS)		*15’*		*15’*						
Hamilton Anxiety Scale (HAMA)			*15’*	*15’ (t0+~6h)*	*15’*	*15’*	*15’*			
Rumination-Reflection Questionnaire (RRQ)			*15’*		*15’*		*15’*	*15’*	*15’*	*15’*
Hopelessness Scale (HS-Krampen)		*15’*			*15’*					

Treatment Emergent Symptom Scale (DOTES, TWIS)/ Adverse Events	*10'*	*10'*	*10'*	*10' (t0+~6h)*	*10'*	*10'*	*10'*			
Therapy Questionnaire	*5'*							*5'*	*5'*	*5'*
Stress Questionnaire								*10'*	*10'*	*10'*

13.2 Assessments of Outcomes

13.2.1 Assessment of Primary Outcome

The primary outcome will be depression severity change following psilocybin treatment compared to placebo, as measured by the score in the Montgomery Asberg Depression Scale (MADRS) at screening, at baseline, and following psilocybin administration (on visit 5, 6 and 7) during study visits. All interviews, scores, and clinical assessments will be performed by an experienced clinician (either physician or psychologist) and will be performed in the context of the study visits. The clinician-rated MADRS is one of the most commonly used symptom severity scales to evaluate the efficacy of antidepressant treatment, and has been used in recent clinical trials of ketamine efficacy in depression. In addition to the MADRS, treatment outcome will also be rated using the standard Beck Depression Inventory (BDI), and the Clinical Global Impressions Scale (CGI) at screening, at preclinical visit, at baseline, and following psilocybin administration (on visit 5, 6 and 7). In addition, the BDI will be administered six weeks as well as approximately one and three months (-/+ 10 days) post-treatment in order to capture long-term effects on depressive symptomology and the subjective need for therapeutic interventions.

13.2.2 Assessment of Secondary Outcomes

The effects of psilocybin compared to placebo on depression symptom profiles is of additional interest in this study. Similar to the assessment of the primary outcome measures, an experienced psychiatrist will rate these secondary endpoints in the context of the study visits. Secondary endpoints include: the Snaith-Hamilton-Pleasure Scale, the Hamilton Anxiety Scale, the Rumination-Reflection Questionnaire, the Emotion-Regulation Questionnaire, and the Hopelessness Scale, and will be measured at at baseline, and following psilocybin administration (2, 8, and 14 days post-treatment). In addition, the MET depression test battery is being used to assess the effects of psilocybin compared to placebo on neurocognitive markers of depression. The MET depression test battery is a highly validated and standardized computer software and will be performed by the patients in a quiet testing room with access to a computer at baseline, and following psilocybin administration (2, 8 and 14 days post-treatment). Furthermore, functional Magnetic Resonance Imaging (fMRI) is being used to assess the effects of psilocybin

compared to placebo on brain functioning markers of depression. BOLD-signal changes in response to the paradigms described in 5.2 will be compared between psilocybin and placebo condition. FMRI measurement will be performed at baseline, and following psilocybin administration (2 and 14 days post-treatment). All FMRI measurements will be performed using the Philips Achieva 3T MR-System equipped with a 32-channel receive head coil and MultiTransmit parallel RF transmission located at the MRI center of the Department of Psychiatry, Psychotherapy and Psychosomatics, University Hospital for Psychiatry. Participants will be instructed to lie as still as possible in the scanner. FMRI data are acquired using a whole brain gradient-echo EPI sequence (TR=2500ms, TE=35ms, slice thickness 3mm, 40 axial slices, no slice gap, field of view 240x240mm2, in-plane resolution 3×3mm, SENSE reduction factor 2.0). Additionally, high-resolution anatomical images (voxel size=1×1×1mm) are acquired using a standard T1-weighted 3-D MP-RAGE sequence. Finally, the effects of psilocybin compared to placebo on BDNF, a neurotrophic marker of antidepressant efficacy, will be collected at baseline, and following psilocybin administration (on visit 5, 6 and 7), always at the same time in the afternoon. Serum and plasma BDNF levels will be assessed at the Neurobiology Laboratory for Brain Aging and Mental Health, Psychiatric University Clinic, University of Basel, Switzerland.

13.2.3 Assessment of Other Outcomes of Interest

A routine lab, medical history, ECG, vital signs, and clinical interview will be conducted and assessed at the screening day to ensure mental and physical health. Urine drug and pregnancy tests will be performed at screening days. Standardized and validated questionnaires as described in section 5.2 will be completed at screening and follow-up and after each MRI measurement.

13.2.4 Assessment of Safety Outcomes

13.2.4.1 Adverse Events

In this study, the efficacy of psilocybin treatment for major depression will be systematically assessed. Although this study is not a systematic investigation of safety, this study will also assess safety and tolerability of psilocybin treatment in depressed patients. The following safety endpoints will be used at each visit: occurrence of adverse events (AEs), psychological and physical well-being, suicidality, vital signs, body weight, and use of concomitant medication. Clinical study participants will be routinely questioned about AEs at study visits. The well-being of the participants will be ascertained by neutral questioning ("How are you?"). All observed or volunteered abnormal safety endpoints will be recorded in the CRF. The investigator will promptly review documented AEs or other abnormal safety endpoints to determine if

- the abnormal safety endpoint should be classified as an AE,
- if there is a reasonable possibility that the AE was caused by the investigational drug, and
- if the AE meets the criteria for a serious AE (SAE).

The Investigator is responsible for SAE reporting to the CEC (see Section 10 for AE definition and procedures).

13.2.4.2 Laboratory Parameters

A routine lab will be conducted at the screening day to ensure physical health. Urine drug and pregnancy tests will be performed at screening days.

13.2.4.3 Vital Signs

Heart beat and blood pressure will measured at each visit in supine position after 5 minutes resting. During drug treatment session, vital signs will be measured before and during treatment sessions in 30-60 minutes intervals.

13.2.5 Assessments in Participants Who Prematurely Stop the Study

For whatever reason a participant or an investigator decides that a participant should withdraw from the study, the investigator will make efforts to conduct an exit visit to assess the safety and well-being of the participant. The exit visit will include vital signs, safety assessments, and reasons for withdrawal. The exit visit will be performed as soon as possible after the withdrawal. If a participant does not agree to an exit visit, a telephone interview will be performed instead, and the participant will be questioned on physical well-being, safety issues, and reasons for withdrawal. When a participant withdraws from the study, the data collected on the participant to the point of withdrawal remains part of the study database and will not be removed.

13.3 Procedures at Each Visit

13.3.1 Pre-Screening (Phone Call)

A telephone screening will be performed by a physician or psychologist specifically trained for the screening procedure prior to the first visit for initial screening and information purposes. The following procedures will be performed:

- Phone Screening Questionnaire
- Prior Medication Record
- SCID-I Section A
- SCID-I Section B

13.3.2 Visit 1 or Screening Visit

Visit 1 will be scheduled between 42 and 5 days before visit 4 and will last about 5 hours. The following exams/tests will be performed:

- Patient information and informed consent
- Structured clinical interview (will be tape-recorded)
- Mental state examination, including assessment of suicidality (will be tape-recorded)
- Questionnaires (self-administered): BDI, SCL-90R, MWT-B, - STEPP, TQ. SKID-II
- Clinician-administered ratings: Drogenanamnese, MADRS, CGI, HAM-A, STEPT
- Physical examination, routine blood test, urine drug test, urine pregnancy test, electrocardiogram (ECG)
- Vital signs & weight
- Safety assessment
- Clinician: Confirmation of In- and Exclusion criteria
- Instruction of patient to write a resume (resp. important life events/ persons)

13.3.3 Visit 2 or Pre-investigation Visit

Visit 2 will be scheduled on day -3 (+/- 2 days) and will last about 4 hours. The following exams/tests will be performed (in chronological order):

- Psychological Counselling Session (will be tape-recorded)
- Further biographical, medical, psychiatric and family history (will be tape-recorded)
- Mental state examination, including assessment of suicidality,(will be tape-recorded)
- Questionnaires (self-administered): BDI, FSCS, SHAPS, HS-Krampen, STEPP
- Clinician-administered ratings: MADRS, CGI, HAM-A, STEPT
- Vital signs & weight
- Safety assessment

13.3.4 Visit 3 or Baseline Measures Visit

Visit 3 will be scheduled 24 hours before day 0 (+/- 0 days) and will last about 6 hours. The following exams/tests will be performed:

- MRI Safety Form
- Drug urine test
- FMRI InvestigationMET
- Blood BDNF sampling (exact time: 14:15hrs)
- Questionnaires (self-administered): BDI, RRQ, ERQ , STEPP
- Clinician-administered ratings: Drogenanamnese, MADRS, CGI, HAM-A, STEPT
- Psychological Counselling Session (will be tape-recorded)
- Mental state examination, including assessment of suicidality (will be tape-recorded)
- Vital signs & weight
- Safety assessment

13.3.5 Visit 4 or Treatment Session Visit

Visit 4 will be scheduled on day 0 (+/- 0 days) between 08:00hrs and 11:00hrs and will last about 8 hours. The following exams/tests will be performed:

- Administer study medication (exact time: 09:00hrs (+/- 2 hours))
- Psychological Counselling Session (will be tape-recorded)
- Mental state examination, including assessment of suicidality, (will be tape-recorded)
- Vital signs & weight
- Safety assessment
- Questionnaires (self-administered): BDI, STEPP, 5D-ASC
- Clinician-administered ratings: Drogenanamnese, MADRS, CGI, HAM-A, STEPT
- Instruction of patient to write protocol

13.3.6 Visit 5 or Post-Treatment Measures Visit

Visit 5 will be scheduled 48 hours after day 0 (+/- 0 days) and will last about 6 hours. The following exams/tests will be performed:

- MRI Safety Form
- Drug urine test
- FMRI Investigation
- MET Investigation
- Questionnaires (self-administered): BDI,, RRQ, ERQ, STEPP
- Clinician-administered ratings: Drogenanamnese, MADRS, HAM-A, CGI, STEPT, Psychological Counselling Session, including discussion of protocol written by patient (will be tape-recorded)

- Mental state examination, including assessment of suicidality (will be tape-recorded)
- Vital signs & weight
- Safety assessment

13.3.7 Visit 6 or Follow-up Visit

Visit 6 will be scheduled 8 days after day 0 (+/- 2 days) and will last about 3 hours. The following exams/tests will be performed:

- Psychological Counselling Session (will be tape-recorded)
- Mental state examination, including assessment of suicidality, (will be tape-recorded)
- Blood BDNF sampling (exact time: 14:15hrs)
- MET Investigation
- Questionnaires (self-administered): BDI, STEPP
- Clinician-administered ratings: MADRS, CGI, HAM-A, STEPT
- Vital signs & weight
- Safety assessment

13.3.8 Visit 7 or Follow-up Measures and Closure Visit

Visit 7 will be scheduled 14 hours days after day 0 (+/- 2 days) and will last about 7 hours. The following exams/tests will be performed:

- MRI Safety Form
- Drug urine test
- FMRI Investigation
- MET Investigation
- Questionnaires (self-administered): BDI, SHAPS, RRQ, ERQ, HS-Krampen, STEPP, FSCS, SCL-90-R
- Clinician-administered ratings: Drogenanamnese, MADRS, CGI, HAM-A, STEPTPsychological Counselling Session (will be tape-recorded)
- Mental state examination, including assessment of suicidality,(will be tape-recorded)
- Vital signs
- Safety assessment

13.3.9 Follow-up Measures six-weeks post-treatment

The first follow-up measurement will be conducted one month after treatment in an online-survey format and consists of the following questionnaires

- BDI
- RRQ
- ERQ
- SCL-90-R
- Drogenanamnese
- Therapy questionnaire
- Stress questionnaire

13.3.10 Follow-up Measures 3-month post-treatment

The first follow-up measurement will be conducted three month after treatment in an online-survey format and consists of the following questionnaires

- BDI
- RRQ
- ERQ
- SCL-90-R
- Drogenanamnese
- Therapy questionnaire
- Stress questionnaire

13.3.11 Follow-up Measures 6-month post-treatment

The first follow-up measurement will be conducted three month after treatment in an online-survey format and consists of the following questionnaires

- BDI
- RRQ
- ERQ
- SCL-90-R
- Drogenanamnese
- Therapy questionnaire
- Stress questionnaire

14 SAFETY

During the entire duration of the study, all adverse events (AE) and all serious adverse events (SAEs) are collected, fully investigated and documented in source documents and case report forms (CRF). Study duration encompassed the time from when the participant signs the informed consent until the last protocol-specific procedure has been completed.

14.1 Definition of (Serious) Adverse Events and Other Safety Related Events

Adverse events

Adverse events (AEs) are defined as any untoward medical occurrence in a patient or clinical investigation participant administered a pharmaceutical product and which does not necessarily have a causal relationship with this treatment. An AE can therefore be any unfavourable and unintended sign (including an abnormal laboratory finding), symptom, or disease temporally associated with the use of a medicinal study product, whether or not related to the medicinal study product.

An AE may also consist of a new disease, an exacerbation of a pre-existing illness or condition, a recurrence of an intermittent illness or condition, a set of related signs or symptoms, or a single sign or symptom.

AEs observed by the investigator and/or reported by the participant must be reported in the CRF during the entire study period, i.e. the period of time from the first (= signature of informed consent) to the last protocol-specific procedure regardless of the medicinal study product relation assessment.

For all AEs, sufficient information will be pursued and/or obtained so as to permit an adequate determination of the outcome of the event (i.e., whether the event should be classified as an SAE) and an assessment of the causal relationship between the AE and the investigational drug or study treatment(s).

Whenever available, the underlying disease or condition for which a therapeutic or diagnostic procedure is required should be reported as the AE term. Surgeries or other invasive procedures that had already been planned prior to the start of the study do not have to be documented as AEs. These planned procedures will be recorded in the CRF by the investigator at the baseline visit. It is not important if the condition was known before enrolment, only if the procedure was planned before.

Serious Adverse Event

An SAE is any untoward medical occurrence that at any dose results in

• results in death,

• is life-threatening,

• requires participant hospitalization or prolongation of current hospitalization,

• results in persistent or significant disability/incapacity, or

• is a congenital anomaly/birth defect,

• any important medical event and any event which, though not included in the above, may jeopardise the participant or may require intervention to prevent one of the outcomes listed above.

Any other medically important condition that may be not immediately life-threatening or results in death or hospitalization but may jeopardize the participant or may require intervention to prevent one of the outcomes listed above should also usually (i.e. based on medical and scientific judgment) be considered serious. For example: intensive treatment at home for allergic bronchospasm; certain laboratory abnormalities (e.g. blood dyscrasias); convulsions that do not result in hospitalisation; development of drug dependency or drug abuse.

SAEs should be followed until resolution or stabilisation. Participants with ongoing SAEs at study termination (including safety visit) will be further followed up until recovery or until stabilisation of the disease after termination.

Unexpected Adverse Drug Reaction

An “unexpected” adverse drug reaction is an adverse reaction, the nature or severity of which is not consistent with the applicable product information (e.g. Investigator’s Brochure for drugs that are not yet approved and Product Information for approved drugs, respectively).

Suspected unexpected serious adverse reaction (SUSAR)

A serious adverse reaction, the nature or severity of which is suspected to be not consistent with the applicable product information e.g. Investigator’s Brochure for drugs that are not yet approved and Product Information for approved drugs, respectively).

Safety Signals

All suspected new risks and relevant new aspects of known adverse reactions that require safety-related measures.

14.2 Recording of (Serious) Adverse Events and Other Safety Related Events

Clinical investigators and ultimately the protocol Principal Investigator (PI) have the primary responsibility for AE identification, documentation, grading, and assignment of attribution to the investigational agent/intervention.

Clinical study participants will be routinely questioned about AEs at study visits. The well-being of the participants will be ascertained by neutral questioning ("How are you?"). The investigator is responsible for reporting all AEs occurring during the course of the study.

All observed or volunteered adverse drug events (serious or non-serious) and abnormal test findings, regardless of treatment group or suspected causal relationship to the investigational drug or study treatment(s) will be recorded in the patient file and subsequently in the CRF.

AEs or abnormal test findings felt to be associated with the study treatment(s) will be followed until the event (or its sequelae) or the abnormal test finding resolves or stabilizes at a level acceptable to the investigator.

An abnormal test finding will be classified as an AE if one or more of the following criteria are met:

- The test finding is accompanied by clinical symptoms.
- The test finding necessitates additional diagnostic evaluation(s) or medical/surgical intervention; including significant additional concomitant drug treatment or other therapy
  Note: simply repeating a test finding, in the absence of any of the other listed criteria, does not constitute an AE.
- The test finding leads to a change in study dosing or discontinuation of participant participation in the clinical study.

All AEs, serious and non-serious, will be fully documented in the appropriate CRF. For each AE, the investigator will provide the onset, duration, intensity, treatment required, outcome and action taken with the investigational product.

14.3 Assessment of (Serious) Adverse Events and Other Safety Related Events

The investigator will promptly review documented AEs and abnormal test findings to determine if

- the abnormal test finding should be classified as an AE,
- if there is a reasonable possibility that the AE was caused by the investigational drug or study treatment(s), and
- if the AE meets the criteria for an SAE.

The intensity of an AE will be assessed by the investigator as being

- mild (hardly noticeable, negligible impairment of well-being),

- moderate (marked discomfort, but tolerable without immediate relief), or
- severe (overwhelming discomfort, calling for immediate relief).

The assessment of causality to the study drug by the investigator is done according to the following definitions:

Unrelated	• The event started in no temporal relationship to medicinal product applied and<br>• The event can be definitely explained by underlying diseases or other situations.
Related	• The event started in a plausible temporal relationship to medicinal product applied and<br>• The event cannot be definitely explained by underlying diseases or other situations.

14.4 Reporting of Serious Adverse Events and Other Safety Related Events

The Investigator is responsible for SAE reporting to the CEC according to the following details:

- Reporting to CEC any SAE which resulted in death:

  - **without delay**, and no later than **7 calendar days**.

- Reporting to CEC of fatal SAEs if evaluated as “suspected”, “unexpected” and “drug related” (SUSAR)

  - **without delay** and no later than **7 calendar days** following awareness that event meets criteria for an SUSAR.

- Reporting to CEC of non-fatal SAEs if evaluated as “suspected”, “unexpected” and “drug related” (SUSAR):

  - **promptly** and no later than **15 calendar days** following awareness that event meets criteria for a SUSAR.

- All other SAEs will be summed up in the **annual safety update report**.

The Sponsor is responsible for SAE reporting to Swissmedic according to the following details:

- Compliance with the regulatory requirements of Swissmedic regarding prompt reporting of unexpected SAEs for which a causal relationship with the study drug cannot be ruled out.

- Reporting to Swissmedic of fatal SAEs if evaluated as “suspected”, “unexpected” and “drug related” (SUSAR):

  - **without delay** and no later than **7 calendar days** following awareness that event meets criteria for a SUSAR;

- Reporting to Swissmedic of non-fatal SAEs if evaluated as “suspected”, “unexpected” and “drug related” (SUSARs):

- **promptly** and no later than **15 calendar days** following awareness that event meets criteria for a SUSAR.

- Sending Annual Safety Reports (ASR), starting one year after the date of notification to Swissmedic. These reports should contain:
  - A concise critical summary of the safety profile of the drug studied as well as the safety issues that have arisen;
  - A listing of all SUSARs that have occurred in Switzerland and at international level (if applicable);
  - Ideally all adverse drug reactions at international level.
  - The accompanying letter provided with the Annual Safety Report should contain a short summary of the status of the clinical trial in Switzerland (number of centres open/closed, number of patients recruited/recruitment closed, and number of SAR/SUSAR.

Reporting of Safety Signals
All suspected new risks and relevant new aspects of known adverse reactions that require safety-related measures, i.e. so called safety signals, must be reported to the Sponsor-Investigator within 24 hours. The Investigator must report the safety signals within 7 days to the local Ethics Committee (local event via local Investigator) and the Sponsor to Swissmedic, respectively.

Reporting and Handling of Pregnancies

Pregnant participants must be immediately withdrawn from the clinical study. All pregnancies occurring during the treatment phase of the study and within 30 days after discontinuation of study medication have to be reported to the Sponsor-Investigator within 24 hours of the investigational sites knowledge of the pregnancy on the Initial Pregnancy Report Form. The Sponsor-Investigator will contact the attendant physician by phone during pregnancy and after the estimated date of delivery to enquire about course and outcome of the pregnancy. Course of the pregnancy and health status of the new born child have to be documented on the Follow-Up Pregnancy Report Form.

14.5 Follow up of (Serious) Adverse Events

Participants terminating the study (either regularly or prematurely) with

- reported ongoing SAE, or
- any ongoing AEs of laboratory values or of vital signs being beyond the alert limit

will return for a follow-up investigation. This visit will take place up to 30 days after terminating the treatment period. Follow-up information on the outcome will be recorded on the respective AE page in the CRF. All other information has to be documented in the source documents. Source data has to be available upon request.

In case of participants lost to follow-up, efforts should be made and documented to contact the participant to encourage him/her to continue study participation as scheduled. In case of minor AEs a telephone call to the participants may be acceptable.

All new SAE or pregnancies that the investigators will be notified of within 30 days after discontinuation of study medication have to be reported in appropriate report forms and in the CRF if required.

Follow-up investigations may also be necessary according to the investigator's medical judgment even if the participant has no AE at the end of the study. However, information related to these investigations does not have to be documented in the CRF but must be noted in the source documents.

15 STATISTICAL METHODS

15.1 Hypothesis

Based on previous studies, we primarily hypothesize that psilocybin will induce rapid (within 48 hours post-treatment) and sustained (<7 days post-treatment) antidepressant effects in depressed patients. In terms of hypothesis testing: the primary null hypothesis of our study says that MADRS change scores at 48 hours post-treatment from baseline are not statistically different between the psilocybin and the placebo group. We are confident that our study will reject the null hypothesis at a probability of $p<0.05$ (two-tailed). We further hypothesize that psilocybin will particularly reduce anhedonia, anxiety, rumination, and hopelessness in depressed patients; that psilocybin will improve neurocognitive measures of depression severity (CANTAB depression test battery), decrease resting-state functional connectivity, increase task-related BOLD fMRI signals in the prefrontal cortex, and decrease task-related BOLD fMRI signals in the amygdala; and that psilocybin will increase blood/ serum BDNF levels in relation to a decrease of MADRS depression severity (probability level for rejection of null hypothesis <0.05 for all secondary endpoints).

15.2 Determination of Sample Size

Our previous studies on brain activity during altered states of consciousness induced with hallucinogenic substances in healthy volunteers revealed effect sizes of Cohen's f = 0.53-1.0 (Kraehenmann et al. 2014; Kometer et al. 2012; Schmidt et al. 2013; Bernasconi et al. 2013). Previous clinical studies investigating rapid antidepressant efficacy of the hallucinogen ketamine in depressed patients showed effect sizes of Cohen's d = 0.68-1.46 for MADRS scores (Zarate et al. 2006a; Murrough et al. 2013). With the conservative assumption of f = 0.5, an alpha-probability of 0.05 (two-sided) and a power of 0.8 (mixed-model ANOVA), we would need at least 22 subjects per treatment arm to detect significant differences in each experiment. From a clinical standpoint, a sample size of N=30 subjects per treatment arm is reasonable given the lack of previous efficacy studies of psilocybin in depression.

15.3 Statistical Criteria of Termination of Trial

15.3.1 Criteria of stopping for benefit

If the interim analysis shows that the sample size of N=15 is sufficient to show significant effects of psilocybin on both primary and secondary endpoints (symmetric stopping boundaries at $p < 0.001$) and that further continuation of the study will be unlikely to yield an additional effect of psilocybin, the trial may be terminated earlier to save resources. However, early stopping for benefit may prevent additional insights into unforeseen beneficial effects on depression sub-groups or individual symptom constellations, because such effects may only be analyzed post-hoc. Therefore, and given the low risks of harm to study participants, the trial shall be continued until the final sample size is reached, if not otherwise advised by the principal investigator or the data monitoring committee.

15.3.2 Criteria of stopping for futility

The trial will not be stopped in case of futility, unless the principal investigator or the data monitoring committee advices otherwise.

15.3.3 Criteria of stopping for harm

The stopping rules for harm in this trial are not statistically defined. The trial will be terminated by the principal investigator if unanticipated problems associated with unexpected serious harm to trial participants or with possible risks of harm to participants have taken place.

15.4 Planned Analyses

15.4.1 Datasets to be Analysed, Analysis Populations

The primary efficacy analysis will be done on the intention-to-treat population (ITT), i.e. all patients randomized who received psilocybin treatment and had at least one assessment of efficacy 48 hours post-treatment.

15.4.2 Primary Analysis

The efficacy analysis on the primary endpoint (depression severity score, MADRS) will be done by the trial statistician after reaching the full sample size of N=2x30 study completers. Study completers are patients who have completed all assessments up to visit 7. Comparisons between psilocybin and placebo will be performed using a mixed-model analysis of variance (ANOVA) to compare mean MADRS change from baseline at the different time points (2, 8, 14 days post-treatment).

15.4.3 Secondary Analyses

The efficacy analysis on the secondary endpoints will be done by the trial statistician after reaching the full sample size of N=2x30 study completers. Study completers are patients who have completed all assessments up to visit 7. Comparisons between psilocybin and placebo will be performed using a mixed-model analysis of variance (ANOVA) to compare secondary endpoint change from baseline at the different time points (2, 8, 14 days post-treatment). To further evaluate the proportion of responders and remitters at 2, 8, and 14

days post-treatment, a McNemar test will be used for the study completers and the results were Bonferroni corrected for the number of points examined.

15.4.4 Interim Analyses

An interim efficacy analysis on the primary endpoints will be performed by the trial statistician after reaching a sample size of N=2x15 study completers. Study completers are patients who have completed all assessments up to visit 7.Comparisons between psilocybin and placebo will be performed using a mixed-model analysis of variance (ANOVA) to compare endpoint change from baseline at the different time points (2, 8, 14 days post-treatment). The interim analysis will inform the stopping rules for benefit (see 11.3.1).

15.4.5 Safety Analysis

Although this study is not a systematic investigation of safety, this study will also clinically assess safety and tolerability of psilocybin treatment in depressed patients. The following safety endpoints will be clinically assessed by the investigators at each visit: occurrence of adverse events (AEs), psychological and physical well-being, suicidality, vital signs, body weight, and use of concomitant medication.

15.4.6 Deviation(s) from the Original Statistical Plan

In case of slow recruitment rates the interim analysis may already be performed at sample size N=2x10.

15.5 Handling of Missing Data and Drop-Outs

The primary efficacy analysis will be done using the last observation carried forward (LOCF) approach on the intention-to-treat population (ITT), i.e. all patients randomized who received psilocybin treatment and had at least one assessment of efficacy 2 days post-treatment will be analyzed. Drop-outs before the primary efficacy assessment 2 days post-treatment will be replaced. The Observed Cases (OC) approach, in which only the observed values will be analyzed, will be used for supportive analyses.

16 DATA QUALITY ASSURANCE AND CONTROL

The Sponsor-Investigator is implementing and maintaining quality assurance and quality control systems with written SOPs and Working Instructions to ensure that trials are conducted and data are generated, documented (record), and reported in compliance with the protocol, GCP, and applicable regulatory requirement(s). The Principal Investigators at must have a manual of the relevant SOPs and WIs for the study on site and is responsible for proper training of all involved study personnel for the respective procedures. Monitoring and Audits will be conducted during the course of the study for quality assurance purposes.

16.1 Data Handling and Record Keeping / Archiving

The study will strictly follow the protocol. If any changes become necessary, they must be laid down in an amendment to the protocol. All amendments of the protocol must be signed by the Sponsor-Investigator and submitted to CEC and Swissmedic.

16.1.1 Case Report Forms

The investigators will use electronic case report forms (eCRF), one for each enrolled study participant, to be filled in with all relevant data pertaining to the participant during the study. All participants who either entered the study or were considered not-eligible or were eligible but not enrolled into the study additionally have to be documented on a screening log. The investigator will document the participation of each study participant on the enrollment log. The correct data must be inserted, (up)-dated and initialed by the investigator. Data that are not available or not done should be made clear by adding 'not applicable' (NA). A declaration ensuring accuracy of data recorded in the case report forms must be electronically signed by the investigator. eCRFs must be kept current to reflect participant status at each phase during the course of study. Participants must not to be identified in the eCRF by name. Appropriate coded identification (e.g. Participant Number) must be used. Initials must not be used in combination with the date of birth in the eCRF for identification of the study participant (combination of initials and year of birth possible).

It must be assured that any authorized person, who may perform data entries and changes in the eCRF, can be identified. A list with signatures and initials of all authorized persons will be filed in the study site file and the trial master file, respectively. Documented medical histories will be maintained. These records will also include the following: laboratory and other medical test results (ECGs, MRIs.) which must be kept on file with the individual participants source documents. The investigators assure to perform a complete and accurate documentation of the participant data in the eCRF. All data entered into the eCRF must also be available in the individual participant file as print-outs by either the investigator or another responsible person assigned by the investigator. Essential documents must be retained for at least 10 years after the regular end or a premature termination of the respective study (KlinV Art. 45). Any patient files and source data must be archived for the longest possible period of time according to the feasibility of the investigational site.

16.1.2 Specification of Source Documents

The following documents are considered source data, including but not limited to:

• SAE worksheets

• Nurse records, records of clinical coordinators, and

• Medical records from other department(s), or other hospital(s), or discharge letters and correspondence with other departments/hospitals, if participant visited any during the study period and the post study period.

Source data must be available at the site to document the existence of the study participants and substantiate the integrity of study data collected. Source data must include the original documents relating to the study, as well as the medical treatment and medical history of the participant.

The following information (at least but not limited to) should be included in the source documents:

• Demographic data (age, sex)
• Inclusion and Exclusion Criteria details
• Participation in study and signed and dated Informed Consent Forms
• Visit dates
• Medical history and physical examination details
• AEs and concomitant medication (SAE report form IMP)
• Results of relevant examinations
• Laboratory printouts
•Dispensing and return of study drug details (drug accountability log total, drug accountability per patient, drug destruction record)
• Reason for premature discontinuation
• Randomization number (subject randomization log)

16.1.3 Record Keeping / Archiving

All study data must be archived for a minimum of 10 years after study termination or premature termination of the clinical trial. Source documents will be stored in locked cabinets in an office which will be locked when the office is not in use. Identifying data (e.g., consent) will be stored in separate locked cabinets. Blood and urine specimens will be used for diagnostic purposes only and will be destroyed after assessment. Data of individuals who do not sign an informed consent form will be destroyed. The study data will be stored in the archives of the Department of Psychiatry, Psychotherapy and Psychosomatics, Zürich University Hospital for Psychiatry.

16.2 Data Management

Source documents will be stored in locked cabinets in an office which will be locked when the office is not in use. Identifying data (e.g., consent) will be stored in separate locked cabinets. Blood and urine specimens will be used for diagnostic purposes only and will be destroyed after assessment. Data of individuals who do not sign an informed consent form will be destroyed. The study data will be stored in the archives of the Department of Psychiatry, Psychotherapy and Psychosomatics, Zürich University Hospital for Psychiatry.

16.2.1 Data Management System

An electronic password restricted data management system will be used. The system is hosted on a server at the Psychiatric University Hospital Zurich. Only approved study personnel will have access to this system.

16.2.2 Data Security, Access and Back-up

Access to the study data will be restricted. A password system will be utilized to control access. Only approved study staff, as listed in the study site personnel signature authorization form, will have access. Data generated as a result of this study are to be available for inspection on request by the monitors, by the CEC, Swissmedic, and the regulatory health authorities. All forms, diskettes and tapes related to study data will be

kept in locked cabinets. All reports prepared by the approved study staff will be prepared such that no individual subject can be identified.

16.2.3 Analysis and Archiving

Data are extracted from the electronic data management system via direct download from our host server and will be stored on password-protected on-site computers. Participant files are to be stored in numerical order and stored in a secure and accessible place and manner at the Psychiatric University Hospital. Participant files will be maintained in storage for a period of 10 years after completion of the study.

16.2.4 Data Validation

Data integrity will be enforced through a variety of mechanisms. Data will be entered centrally and immediately after data generation. Study personnel will be responsible to verify that the data entered are in the proper format. The approved study staff will be responsible for making appropriate corrections to the original paper forms whenever any data item is changed. Written documentation of changes will be available via electronic logs and audit trails. Study personnel will be trained for data quality purposes before study begin.

16.3 Monitoring

Regular monitoring visits at the investigator’s site prior to the start and during the course of the study will help to follow up the progress of the clinical study, to assure utmost accuracy of the data and to detect possible errors at an early time point. The Sponsor-Investigator organizes independent monitoring for the study.

All original data including all patient files, progress notes and copies of laboratory and medical test results must be available for monitoring. The monitor will review all or a part of the eCRF and written informed consents. The accuracy of the data will be verified by reviewing the above referenced documents.

16.4 Audits and Inspections

A quality assurance audit/inspection of this study may be conducted by the competent authority or CEC, respectively. The quality assurance auditor/inspector will have access to all medical records, the investigator's study related files and correspondence, and the informed consent documentation that is relevant to this clinical study.

The investigator will allow the persons being responsible for the audit or the inspection to have access to the source data/documents and to answer any questions arising. All involved parties will keep the patient data strictly confidential.

16.5 Confidentiality, Data Protection

Direct access to source documents will be permitted for purposes of monitoring, audits and inspections. The PI and the investigators will have access to protocol, dataset, statistical code, etc. during and after the study.

16.6 Storage of Biological Material and Related Health Data

Blood and urine specimens will be used for diagnostic purposes only and will be destroyed after assessment.

17 PUBLICATION AND DISSEMINATION POLICY

After the statistical analysis of this trial the sponsor will make every endeavour to publish the data in a medical journal.

18 FUNDING AND SUPPORT

This study is financially supported with 90’000 CHF by the Swiss Neuromatrix Foundation, Switzerland (Achievement Grant to Prof. Dr. Franz X. Vollenweider). The funders have no role in study design, data collection and analysis, decision to publish, or preparation of a manuscript.

19 INSURANCE

Insurance is covered by “Versicherung für klinische Versuche der Zürich Versicherungs-Gesellschaft AG, Mythenquai 2, 8002 Zürich” for Psychiatric University Hospital Zurich (Policy no.: 14.970.885).

Any damage developed in relation to study participation is covered by this insurance. So as not to forfeit their insurance cover, the participants themselves must strictly follow the instructions of the study personnel. Participants must not be involved in any other medical treatment without permission of the principal investigator (emergency excluded). Medical emergency treatment must be reported immediately to the investigator. The investigator must also be informed instantly, in the event of health problems or other damages during or after the course of study treatment.

The investigator will allow delegates of the insurance company to have access to the source data/documents as necessary to clarify a case of damage related to study participation. All involved parties will keep the patient data strictly confidential.

A copy of the insurance certificate will be placed in the Investigator’s Site File.

20 REFERENCES

American Psychiatric Association (2011): Diagnostic and statistical manual of mental disorders. DSM-IV-TR : Text revision. 4. ed. Washington DC.

Bari, Andrea; Theobald, David E.; Caprioli, Daniele; Mar, Adam C.; Aidoo-Micah, Alex; Dalley, Jeffrey W.; Robbins, Trevor W. (2010): Serotonin modulates sensitivity to reward and negative feedback in a probabilistic reversal learning task in rats. In: *Neuropsychopharmacology* 35 (6), S. 1290–1301. DOI: 10.1038/npp.2009.233.

Baumeister, David; Barnes, Georgina; Giaroli, Giovanni; Tracy, Derek (2014): Classical hallucinogens as antidepressants? A review of pharmacodynamics and putative clinical roles. In: *Ther Adv Psychopharmacol* 4 (4), S. 156–169. DOI: 10.1177/2045125314527985.

Beats, B. C.; Sahakian, B. J.; Levy, R. (1996): Cognitive performance in tests sensitive to frontal lobe dysfunction in the elderly depressed. In: *Psychol Med* 26 (3), S. 591–603.

Berman, R. M.; Cappiello, A.; Anand, A.; Oren, D. A.; Heninger, G. R.; Charney, D. S.; Krystal, J. H. (2000): Antidepressant effects of ketamine in depressed patients. In: *Biol. Psychiatry* 47 (4), S. 351–354.

Bernasconi, Fosco; Schmidt, André; Pokorny, Thomas; Kometer, Michael; Seifritz, Erich; Vollenweider, Franz X. (2013): Spatiotemporal Brain Dynamics of Emotional Face Processing Modulations Induced by the Serotonin 1A/2A Receptor Agonist Psilocybin. In: *Cereb. Cortex. DOI:* 10.1093/cercor/bht178.

Brunoni, André Russowsky; Lopes, Mariana; Fregni, Felipe (2008): A systematic review and meta-analysis of clinical studies on major depression and BDNF levels: implications for the role of neuroplasticity in depression. In: *Int. J. Neuropsychopharmacol.* 11 (8), S. 1169–1180. DOI: 10.1017/S1461145708009309.

Carhart-Harris, R. L.; Leech, R.; Williams, T. M.; Erritzoe, D.; Abbasi, N.; Bargiotas, T. et al. (2012a): Implications for psychedelic-assisted psychotherapy: functional magnetic resonance imaging study with psilocybin. In: *Br J Psychiatry* 200 (3), S. 238–244. DOI: 10.1192/bjp.bp.111.103309.

Carhart-Harris, Robin L.; Erritzoe, David; Williams, Tim; Stone, James M.; Reed, Laurence J.; Colasanti, Alessandro et al. (2012b): Neural correlates of the psychedelic state as determined by fMRI studies with psilocybin. In: *Proc. Natl. Acad. Sci. U.S.A.* 109 (6), S. 2138–2143. DOI: 10.1073/pnas.1119598109.

Carlson, Paul J.; Diazgranados, Nancy; Nugent, Allison C.; Ibrahim, Lobna; Luckenbaugh, David A.; Brutsche, Nancy et al. (2013): Neural correlates of rapid antidepressant response to ketamine in treatment-resistant unipolar depression: a preliminary positron emission tomography study. In: *Biol. Psychiatry* 73 (12), S. 1213–1221. DOI: 10.1016/j.biopsych.2013.02.008.

Carter, Olivia L.; Burr, David C.; Pettigrew, John D.; Wallis, Guy M.; Hasler, Felix; Vollenweider, Franz X. (2005): Using psilocybin to investigate the relationship between attention, working memory, and the serotonin 1A and 2A receptors. In: *J Cogn Neurosci* 17 (10), S. 1497–1508. DOI: 10.1162/089892905774597191.

Castrén, Eero (2004): Neurotrophic effects of antidepressant drugs. In: *Curr Opin Pharmacol* 4 (1), S. 58–64. DOI: 10.1016/j.coph.2003.10.004.

Catlow, Briony J.; Song, Shijie; Paredes, Daniel A.; Kirstein, Cheryl L.; Sanchez-Ramos, Juan (2013): Effects of psilocybin on hippocampal neurogenesis and extinction of trace fear conditioning. In: *Exp Brain Res* 228 (4), S. 481–491. DOI: 10.1007/s00221-013-3579-0.

Cryan, John F.; O'Leary, Olivia F. (2010): Neuroscience. A glutamate pathway to faster-acting antidepressants? In: *Science* 329 (5994), S. 913–914. DOI: 10.1126/science.1194313.

Diazgranados, Nancy; Ibrahim, Lobna; Brutsche, Nancy E.; Newberg, Andrew; Kronstein, Phillip; Khalife, Sami et al. (2010a): A randomized add-on trial of an N-methyl-D-aspartate antagonist in treatment-resistant bipolar depression. In: *Arch. Gen. Psychiatry* 67 (8), S. 793–802. DOI: 10.1001/archgenpsychiatry.2010.90.

Diazgranados, Nancy; Ibrahim, Lobna A.; Brutsche, Nancy E.; Ameli, Rezvan; Henter, Ioline D.; Luckenbaugh, David A. et al. (2010b): Rapid resolution of suicidal ideation after a single infusion of an N-methyl-D-aspartate antagonist in patients with treatment-resistant major depressive disorder. In: *J Clin Psychiatry* 71 (12), S. 1605–1611. DOI: 10.4088/JCP.09m05327blu.

Duman, Ronald S.; Voleti, Bhavya (2012): Signaling pathways underlying the pathophysiology and treatment of depression: novel mechanisms for rapid-acting agents. In: *Trends Neurosci.* 35 (1), S. 47–56. DOI: 10.1016/j.tins.2011.11.004.

Elliott, R.; Sahakian, B. J.; Herrod, J. J.; Robbins, T. W.; Paykel, E. S. (1997): Abnormal response to negative feedback in unipolar depression: evidence for a diagnosis specific impairment. In: *J. Neurol. Neurosurg. Psychiatr.* 63 (1), S. 74–82.

Fox, Michael D.; Raichle, Marcus E. (2007): Spontaneous fluctuations in brain activity observed with functional magnetic resonance imaging. In: *Nat. Rev. Neurosci.* 8 (9), S. 700–711. DOI: 10.1038/nrn2201.

Franz, M.; Lemke, M. R.; Meyer, T.; Ulferts, J.; Puhl, P.; Snaith, R. P. (1998): Deutsche Version der Snaith-Hamilton-Pleasure-Scale (SHAPS-D). Erfassung von Anhedonie bei schizophrenen und depressiven Patienten. In: *Fortschr Neurol Psychiatr* 66 (9), S. 407–413. DOI: 10.1055/s-2007-995279.

Fu, Cynthia H Y; Williams, Steven C R; Cleare, Anthony J.; Brammer, Michael J.; Walsh, Nicholas D.; Kim, Jieun et al. (2004): Attenuation of the neural response to sad faces in major depression by antidepressant treatment: a prospective, event-related functional magnetic resonance imaging study. In: *Arch. Gen. Psychiatry* 61 (9), S. 877–889. DOI: 10.1001/archpsyc.61.9.877.

Gable, R. S. (1993): Toward a comparative overview of dependence potential and acute toxicity of psychoactive substances used nonmedically. In: *Am J Drug Alcohol Abuse* 19 (3), S. 263–281.

Gable, Robert S. (2004): Comparison of acute lethal toxicity of commonly abused psychoactive substances. In: *Addiction* 99 (6), S. 686–696. DOI: 10.1111/j.1360-0443.2004.00744.x.

Griffiths, R. R.; Richards, W. A.; McCann, U.; Jesse, R. (2006a): Psilocybin can occasion mystical-type experiences having substantial and sustained personal meaning and spiritual significance. In: *Psychopharmacology (Berl.)* 187 (3), S. 268-83; discussion 284-92. DOI: 10.1007/s00213-006-0457-5.

Griffiths, R. R.; Richards, W. A.; McCann, U.; Jesse, R. (2006b): Psilocybin can occasion mystical-type experiences having substantial and sustained personal meaning and spiritual significance. In: *Psychopharmacology (Berl.)* 187 (3), S. 268-83; discussion 284-92. DOI: 10.1007/s00213-006-0457-5.

Griffiths, Roland R.; Johnson, Matthew W.; Richards, William A.; Richards, Brian D.; McCann, Una; Jesse, Robert (2011): Psilocybin occasioned mystical-type experiences: immediate and persisting dose-related effects. In: *Psychopharmacology (Berl.)* 218 (4), S. 649–665. DOI: 10.1007/s00213-011-2358-5.

Griffiths, Rr; Richards, Wa; Johnson, Mw; McCann, Ud; Jesse, R. (2008a): Mystical-type experiences occasioned by psilocybin mediate the attribution of personal meaning and spiritual significance 14 months later. In: *J. Psychopharmacol. (Oxford)* 22 (6), S. 621–632. DOI: 10.1177/0269881108094300.

Griffiths, Rr; Richards, Wa; Johnson, Mw; McCann, Ud; Jesse, R. (2008b): Mystical-type experiences occasioned by psilocybin mediate the attribution of personal meaning and spiritual significance 14 months later. In: *J. Psychopharmacol. (Oxford)* 22 (6), S. 621–632. DOI: 10.1177/0269881108094300.

Grimm, Simone; Ernst, Jutta; Boesiger, Peter; Schuepbach, Daniel; Hell, Daniel; Boeker, Heinz; Northoff, Georg (2009): Increased self-focus in major depressive disorder is related to neural abnormalities in subcortical-cortical midline structures. In: *Hum Brain Mapp* 30 (8), S. 2617–2627. DOI: 10.1002/hbm.20693.

Grinspoon, L.; Bakalar, J. B. (1981): The psychedelic drug therapies. In: *Curr Psychiatr Ther* 20, S. 275–283.

Grob, Charles S.; Danforth, Alicia L.; Chopra, Gurpreet S.; Hagerty, Marycie; McKay, Charles R.; Halberstadt, Adam L.; Greer, George R. (2011a): Pilot study of psilocybin treatment for anxiety in patients with advanced-stage cancer. In: *Arch. Gen. Psychiatry* 68 (1), S. 71–78. DOI: 10.1001/archgenpsychiatry.2010.116.

Grob, Charles S.; Danforth, Alicia L.; Chopra, Gurpreet S.; Hagerty, Marycie; McKay, Charles R.; Halberstadt, Adam L.; Greer, George R. (2011b): Pilot study of psilocybin treatment for anxiety in patients with advanced-stage cancer. In: *Arch. Gen. Psychiatry* 68 (1), S. 71–78. DOI: 10.1001/archgenpsychiatry.2010.116.

Guy, William (1976): ECDEU assessment manual for psychopharmacology. Rev. Rockville, Md.: U.S. Dept. of Health, Education, and Welfare, Public Health Service, Alcohol, Drug Abuse, and Mental Health Administration, National Institute of Mental Health, Psychopharmacology Research Branch, Division of Extramural Research Programs (DHEW publication, no. (ADM) 76-338).

Haile, C. N.; Murrough, J. W.; Iosifescu, D. V.; Chang, L. C.; Al Jurdi, R K; Foulkes, A. et al. (2014): Plasma brain derived neurotrophic factor (BDNF) and response to ketamine in treatment-resistant depression. In: *Int. J. Neuropsychopharmacol.* 17 (2), S. 331–336. DOI: 10.1017/S1461145713001119.

Halpern, John H.; Pope, Harrison G. (2003): Hallucinogen persisting perception disorder: what do we know after 50 years? In: *Drug Alcohol Depend* 69 (2), S. 109–119.

HAMILTON, M. (1959): The assessment of anxiety states by rating. In: *Br J Med Psychol* 32 (1), S. 50–55.

HAMILTON, M. (1960): A rating scale for depression. In: *J. Neurol. Neurosurg. Psychiatr.* 23, S. 56–62.

Hasler, F.; Bourquin, D.; Brenneisen, R.; Bär, T.; Vollenweider, F. X. (1997): Determination of psilocin and 4-hydroxyindole-3-acetic acid in plasma by HPLC-ECD and pharmacokinetic profiles of oral and intravenous psilocybin in man. In: *Pharm Acta Helv* 72 (3), S. 175–184.

Hasler, Felix; Grimberg, Ulrike; Benz, Marco A.; Huber, Theo; Vollenweider, Franz X. (2004): Acute psychological and physiological effects of psilocybin in healthy humans: a double-blind, placebo-controlled dose-effect study. In: *Psychopharmacology (Berl.)* 172 (2), S. 145–156. DOI: 10.1007/s00213-003-1640-6.

Hasler, Gregor; van der Veen, Jan Willem; Tumonis, Toni; Meyers, Noah; Shen, Jun; Drevets, Wayne C. (2007): Reduced prefrontal glutamate/glutamine and gamma-aminobutyric acid levels in major depression determined using proton magnetic resonance spectroscopy. In: *Arch. Gen. Psychiatry* 64 (2), S. 193–200. DOI: 10.1001/archpsyc.64.2.193.

Hautzinger, Martin; Keller, Ferdinand; Kühner, Christine (2006): Beck-Depressions-Inventar. Revision. [2. BDI II Ausgabe]. Frankfurt am Main: Harcourt Test Services.

Hawley, C. J.; Gale, T. M.; Sivakumaran, T. (2002): Defining remission by cut off score on the MADRS: selecting the optimal value. In: *J Affect Disord* 72 (2), S. 177–184.

Johnson, Matthew W.; Sewell, R. Andrew; Griffiths, Roland R. (2012): Psilocybin dose-dependently causes delayed, transient headaches in healthy volunteers. In: *Drug Alcohol Depend* 123 (1-3), S. 132–140. DOI: 10.1016/j.drugalcdep.2011.10.029.

Johnson, Mw; Richards, Wa; Griffiths, Rr (2008): Human hallucinogen research: guidelines for safety. In: *J. Psychopharmacol. (Oxford)* 22 (6), S. 603–620. DOI: 10.1177/0269881108093587.

Kometer, Michael; Schmidt, André; Bachmann, Rosilla; Studerus, Erich; Seifritz, Erich; Vollenweider, Franz X. (2012): Psilocybin biases facial recognition, goal-directed behavior, and mood state toward positive relative to negative emotions through different serotonergic subreceptors. In: *Biol. Psychiatry* 72 (11), S. 898–906. DOI: 10.1016/j.biopsych.2012.04.005.

Kraehenmann, Rainer; Preller, Katrin H.; Scheidegger, Milan; Pokorny, Thomas; Bosch, Oliver G.; Seifritz, Erich; Vollenweider, Franz X. (2014): Psilocybin-Induced Decrease in Amygdala Reactivity Correlates with Enhanced Positive Mood in Healthy Volunteers. In: *Biol. Psychiatry. DOI:* 10.1016/j.biopsych.2014.04.010.

Krampen, Günter (1994): Skalen zur Erfassung von Hoffnungslosigkeit (H-Skalen). Handanweisung. Göttingen [u.a.]: Hogrefe, Verl. für Psychologie.

Kupferschmidt, Kai (2014): High hopes. In: *Science* 345 (6192), S. 18–23. DOI: 10.1126/science.345.6192.18.

Leuner, H. (1966): [Psychotherapy with the aid of hallucinogenic drugs]. In: *Arzneimittelforschung* 16 (2), S. 253–255.

Li, Nanxin; Lee, Boyoung; Liu, Rong-Jian; Banasr, Mounira; Dwyer, Jason M.; Iwata, Masaaki et al. (2010): mTOR-dependent synapse formation underlies the rapid antidepressant effects of NMDA antagonists. In: *Science* 329 (5994), S. 959–964. DOI: 10.1126/science.1190287.

Liu, Rong-Jian; Lee, Francis S.; Li, Xiao-Yuan; Bambico, Francis; Duman, Ronald S.; Aghajanian, George K. (2012): Brain-derived neurotrophic factor Val66Met allele impairs basal and ketamine-stimulated synaptogenesis in prefrontal cortex. In: *Biol. Psychiatry* 71 (11), S. 996–1005. DOI: 10.1016/j.biopsych.2011.09.030.

Maeng, Sungho; Zarate, Carlos A.; Du, Jing; Schloesser, Robert J.; McCammon, Joseph; Chen, Guang; Manji, Husseini K. (2008): Cellular mechanisms underlying the antidepressant effects of ketamine: role of alpha-amino-3-hydroxy-5-methylisoxazole-4-propionic acid receptors. In: *Biol. Psychiatry* 63 (4), S. 349–352. DOI: 10.1016/j.biopsych.2007.05.028.

Mikoteit, Thorsten; Beck, Johannes; Eckert, Anne; Hemmeter, Ulrich; Brand, Serge; Bischof, Roland et al. (2014): High baseline BDNF serum levels and early psychopathological improvement are predictive of treatment outcome in major depression. In: *Psychopharmacology (Berl.)* 231 (15), S. 2955–2965. DOI: 10.1007/s00213-014-3475-8.

Moghaddam, B.; Adams, B.; Verma, A.; Daly, D. (1997): Activation of glutamatergic neurotransmission by ketamine: a novel step in the pathway from NMDA receptor blockade to dopaminergic and cognitive disruptions associated with the prefrontal cortex. In: *J. Neurosci.* 17 (8), S. 2921–2927.

Molendijk, M. L.; Bus, B A A; Spinhoven, Ph; Penninx, B W J H; Kenis, G.; Prickaerts, J. et al. (2011): Serum levels of brain-derived neurotrophic factor in major depressive disorder: state-trait issues, clinical features and pharmacological treatment. In: *Mol. Psychiatry* 16 (11), S. 1088–1095. DOI: 10.1038/mp.2010.98.

Montgomery, S. A.; Asberg, M. (1979): A new depression scale designed to be sensitive to change. In: *Br J Psychiatry* 134, S. 382–389.

Moreno, Francisco A.; Wiegand, Christopher B.; Taitano, E. Keolani; Delgado, Pedro L. (2006): Safety, tolerability, and efficacy of psilocybin in 9 patients with obsessive-compulsive disorder. In: *J Clin Psychiatry* 67 (11), S. 1735–1740.

Murphy, F. C.; Sahakian, B. J.; Rubinsztein, J. S.; Michael, A.; Rogers, R. D.; Robbins, T. W.; Paykel, E. S. (1999): Emotional bias and inhibitory control processes in mania and depression. In: *Psychol Med* 29 (6), S. 1307–1321.

Murrough, James W.; Iosifescu, Dan V.; Chang, Lee C.; Al Jurdi, Rayan K; Green, Charles E.; Perez, Andrew M. et al. (2013): Antidepressant efficacy of ketamine in treatment-resistant major depression: a two-site randomized controlled trial. In: *Am J Psychiatry* 170 (10), S. 1134–1142. DOI: 10.1176/appi.ajp.2013.13030392.

Muschamp, John W.; Regina, Meredith J.; Hull, Elaine M.; Winter, Jerrold C.; Rabin, Richard A. (2004): Lysergic acid diethylamide and [-]-2,5-dimethoxy-4-methylamphetamine increase extracellular glutamate in rat prefrontal cortex. In: *Brain Res.* 1023 (1), S. 134–140. DOI: 10.1016/j.brainres.2004.07.044.

Naughton, Marie; Clarke, Gerard; O'Leary, Olivia F.; Cryan, John F.; Dinan, Timothy G. (2014): A review of ketamine in affective disorders: current evidence of clinical efficacy, limitations of use and pre-clinical evidence on proposed mechanisms of action. In: *J Affect Disord* 156, S. 24–35. DOI: 10.1016/j.jad.2013.11.014.

Nejad, Ayna Baladi; Fossati, Philippe; Lemogne, Cédric (2013): Self-referential processing, rumination, and cortical midline structures in major depression. In: *Front Hum Neurosci* 7, S. 666. DOI: 10.3389/fnhum.2013.00666.

Nichols, David E. (2004): Hallucinogens. In: *Pharmacol. Ther.* 101 (2), S. 131–181. DOI: 10.1016/j.pharmthera.2003.11.002.

Nolen-Hoeksema, S. (2000): The role of rumination in depressive disorders and mixed anxiety/depressive symptoms. In: *J Abnorm Psychol* 109 (3), S. 504–511.

Northoff, Georg; Wiebking, Christine; Feinberg, Todd; Panksepp, Jaak (2011): The 'resting-state hypothesis' of major depressive disorder-a translational subcortical-cortical framework for a system disorder. In: *Neurosci Biobehav Rev* 35 (9), S. 1929–1945. DOI: 10.1016/j.neubiorev.2010.12.007.

Nugent, Allison C.; Diazgranados, Nancy; Carlson, Paul J.; Ibrahim, Lobna; Luckenbaugh, David A.; Brutsche, Nancy et al. (2014): Neural correlates of rapid antidepressant response to ketamine in bipolar disorder. In: *Bipolar Disord* 16 (2), S. 119–128. DOI: 10.1111/bdi.12118.

Outhred, Tim; Hawkshead, Brittany E.; Wager, Tor D.; Das, Pritha; Malhi, Gin S.; Kemp, Andrew H. (2013): Acute neural effects of selective serotonin reuptake inhibitors versus noradrenaline reuptake inhibitors on emotion processing: Implications for differential treatment efficacy. In: *Neurosci Biobehav Rev* 37 (8), S. 1786–1800. DOI: 10.1016/j.neubiorev.2013.07.010.

Passie, Torsten; Seifert, Juergen; Schneider, Udo; Emrich, Hinderk M. (2002): The pharmacology of psilocybin. In: *Addict Biol* 7 (4), S. 357–364. DOI: 10.1080/1355621021000005937.

Phelps, Laura E.; Brutsche, Nancy; Moral, Jazmin R.; Luckenbaugh, David A.; Manji, Husseini K.; Zarate, Carlos A. (2009): Family history of alcohol dependence and initial antidepressant response to an N-methyl-D-aspartate antagonist. In: *Biol. Psychiatry* 65 (2), S. 181–184. DOI: 10.1016/j.biopsych.2008.09.029.

Poo, M. M. (2001): Neurotrophins as synaptic modulators. In: *Nat. Rev. Neurosci.* 2 (1), S. 24–32. DOI: 10.1038/35049004.

Preskorn, Sheldon H.; Baker, Bryan; Kolluri, Sheela; Menniti, Frank S.; Krams, Michael; Landen, Jaren W. (2008): An innovative design to establish proof of concept of the antidepressant effects of the NR2B subunit selective N-methyl-D-aspartate antagonist, CP-101,606, in patients with treatment-refractory major depressive disorder. In: *J Clin Psychopharmacol* 28 (6), S. 631–637. DOI: 10.1097/JCP.0b013e31818a6cea.

Price, Rebecca B.; Nock, Matthew K.; Charney, Dennis S.; Mathew, Sanjay J. (2009): Effects of intravenous ketamine on explicit and implicit measures of suicidality in treatment-resistant depression. In: *Biol. Psychiatry* 66 (5), S. 522–526. DOI: 10.1016/j.biopsych.2009.04.029.

Sanacora, Gerard; Zarate, Carlos A.; Krystal, John H.; Manji, Husseini K. (2008): Targeting the glutamatergic system to develop novel, improved therapeutics for mood disorders. In: *Nat Rev Drug Discov* 7 (5), S. 426–437. DOI: 10.1038/nrd2462.

Satow, L. (2012). Stress-und Coping-Inventar (SCI): Test- und Skalendokumentation. Online im Internet: *URL: http://www.drsatow.de*

Schmidt, André; Kometer, Michael; Bachmann, Rosilla; Seifritz, Erich; Vollenweider, Franz (2013): The NMDA antagonist ketamine and the 5-HT agonist psilocybin produce dissociable effects on structural encoding of emotional face expressions. In: *Psychopharmacology (Berl.)* 225 (1), S. 227–239. DOI: 10.1007/s00213-012-2811-0.

Schmidtke, A.; Fleckenstein, P.; Moises, W.; Beckmann, H. (1988): Untersuchungen zur Reliabilität und Validität einer deutschen Version der Montgomery-Asberg Depression-Rating Scale (MADRS). In: *Schweiz Arch Neurol Psychiatr* 139 (2), S. 51–65.

Sheehan, D. V.; Lecrubier, Y.; Sheehan, K. H.; Amorim, P.; Janavs, J.; Weiller, E. et al. (1998): The Mini-International Neuropsychiatric Interview (M.I.N.I.): the development and validation of a structured diagnostic psychiatric interview for DSM-IV and ICD-10. In: *J Clin Psychiatry* 59 Suppl 20, S. 22-33;quiz 34-57.

Sheline, Yvette I.; Price, Joseph L.; Yan, Zhizi; Mintun, Mark A. (2010): Resting-state functional MRI in depression unmasks increased connectivity between networks via the dorsal nexus. In: *Proc. Natl. Acad. Sci. U.S.A.* 107 (24), S. 11020–11025. DOI: 10.1073/pnas.1000446107.

Skolnick, Phil; Popik, Piotr; Trullas, Ramon (2009): Glutamate-based antidepressants: 20 years on. In: *Trends Pharmacol. Sci.* 30 (11), S. 563–569. DOI: 10.1016/j.tips.2009.09.002.

Strassman, R. J. (1984): Adverse reactions to psychedelic drugs. A review of the literature. In: *J. Nerv. Ment. Dis.* 172 (10), S. 577–595.

Studerus, Erich; Kometer, Michael; Hasler, Felix; Vollenweider, Franz X. (2011a): Acute, subacute and long-term subjective effects of psilocybin in healthy humans: a pooled analysis of experimental studies. In: *J. Psychopharmacol. (Oxford)* 25 (11), S. 1434–1452. DOI: 10.1177/0269881110382466.

Studerus, Erich; Kometer, Michael; Hasler, Felix; Vollenweider, Franz X. (2011b): Acute, subacute and long-term subjective effects of psilocybin in healthy humans: a pooled analysis of experimental studies. In: *J. Psychopharmacol. (Oxford)* 25 (11), S. 1434–1452. DOI: 10.1177/0269881110382466.

Taylor Tavares, Joana V; Clark, Luke; Furey, Maura L.; Williams, Guy B.; Sahakian, Barbara J.; Drevets, Wayne C. (2008): Neural basis of abnormal response to negative feedback in unmedicated mood disorders. In: *Neuroimage* 42 (3), S. 1118–1126. DOI: 10.1016/j.neuroimage.2008.05.049.

Tylš, Filip; Páleníček, Tomáš; Horáček, Jiří (2014): Psilocybin--summary of knowledge and new perspectives. In: *Eur Neuropsychopharmacol* 24 (3), S. 342–356. DOI: 10.1016/j.euroneuro.2013.12.006.

Vollenweider, F. X. (2001): Brain mechanisms of hallucinogens and entactogens. In: *Dialogues Clin Neurosci* 3 (4), S. 265–279.

Vollenweider, F. X.; Leenders, K. L.; Scharfetter, C.; Maguire, P.; Stadelmann, O.; Angst, J. (1997): Positron emission tomography and fluorodeoxyglucose studies of metabolic hyperfrontality and psychopathology in the psilocybin model of psychosis. In: *Neuropsychopharmacology* 16 (5), S. 357–372. DOI: 10.1016/S0893-133X(96)00246-1.

Vollenweider, F. X.; Vollenweider-Scherpenhuyzen, M. F.; Bäbler, A.; Vogel, H.; Hell, D. (1998): Psilocybin induces schizophrenia-like psychosis in humans via a serotonin-2 agonist action. In: *Neuroreport* 9 (17), S. 3897–3902.

Vollenweider, Franz X.; Kometer, Michael (2010a): The neurobiology of psychedelic drugs: implications for the treatment of mood disorders. In: *Nat. Rev. Neurosci.* 11 (9), S. 642–651. DOI: 10.1038/nrn2884.

Vollenweider, Franz X.; Kometer, Michael (2010b): The neurobiology of psychedelic drugs: implications for the treatment of mood disorders. In: *Nat. Rev. Neurosci.* 11 (9), S. 642–651. DOI: 10.1038/nrn2884.

Walter, Martin; Henning, Anke; Grimm, Simone; Schulte, Rolf F.; Beck, Johannes; Dydak, Ulrike et al. (2009): The relationship between aberrant neuronal activation in the pregenual anterior cingulate, altered glutamatergic metabolism, and anhedonia in major depression. In: *Arch. Gen. Psychiatry* 66 (5), S. 478–486. DOI: 10.1001/archgenpsychiatry.2009.39.

Wolkowitz, Owen M.; Wolf, Jessica; Shelly, Wendy; Rosser, Rebecca; Burke, Heather M.; Lerner, George K. et al. (2011): Serum BDNF levels before treatment predict SSRI response in depression. In: *Prog. Neuropsychopharmacol. Biol. Psychiatry* 35 (7), S. 1623–1630. DOI: 10.1016/j.pnpbp.2011.06.013.

Zarate, Carlos A.; Singh, Jaskaran B.; Carlson, Paul J.; Brutsche, Nancy E.; Ameli, Rezvan; Luckenbaugh, David A. et al. (2006a): A randomized trial of an N-methyl-D-aspartate antagonist in treatment-resistant major depression. In: *Arch. Gen. Psychiatry* 63 (8), S. 856–864. DOI: 10.1001/archpsyc.63.8.856.

Zarate, Carlos A.; Singh, Jaskaran B.; Quiroz, Jorge A.; Jesus, Georgette de; Denicoff, Kirk K.; Luckenbaugh, David A. et al. (2006b): A double-blind, placebo-controlled study of memantine in the treatment of major depression. In: *Am J Psychiatry* 163 (1), S. 153–155. DOI: 10.1176/appi.ajp.163.1.153.

DATA, D. (1997). *Structured clinical interview for DSM-IV axis I disorders*. American Psychiatric Press, Washington.
