# Supplementary material for: Single-dose psilocybin-assisted therapy in major depressive disorder: A placebo-controlled, double-blind, randomised clinical trial
Source: eClinicalMedicine. 2022 Dec 28;56:101809. doi: 10.1016/j.eclinm.2022.101809 (PMC9830149; doi:10.1016/j.eclinm.2022.101809)
Supplement: Summary German [file mmc3.docx]

|  |  |  |  |  | **PLACEBO** | |  | **PSILOCYBIN** | |  | **T-TEST** | | | |
| --- | --- | --- | --- | --- | --- | --- | --- | --- | --- | --- | --- | --- | --- | --- |
| **Questionnaire** |  | **Scale** |  |  | **Baseline  mean (SD)** | **14 days  mean (SD)** |  | **Baseline  mean (SD)** | **14 days  mean (SD)** |  | **mean difference** | **95% CI** | **P-value** | **Cohens' *d*** |
|  |  |  |  |  |  |  |  |  |  |  |  |  |  |  |
| **SCL-90-R** |  | Somatization |  |  | 0·58 (0·41) | 0·55 (0·56) |  | 0·43 (0·36) | 0·35 (0·38) |  | -0·09 | -0·07 to 0·47 | 0·14 | 0·42 |
|  |  | Obsessive-compulsive | |  | 1·26 (0·57) | 1·04 (0·76) |  | 1·36 (0·43) | 0·79 (0·47) |  | -0·57 | -0·11 to 0·62 | 0·16 | 0·40 |
|  |  | Interpersonal sensitivity | |  | 1·15 (0·80) | 1.05 (0·76) |  | 1·29 (0·75) | 0·83 (0·64) |  | -0·46 | -0·18 to 0·62 | 0·28 | 0·31 |
|  |  | Anger-hostility |  |  | 0·65 (0·54) | 0·61 (0·60) |  | 0·56 (0·49) | 0·38 (0·33) |  | -0·19 | -0·05 to 0·51 | 0·099 | 0·48 |
|  |  | Anxiety |  |  | 0·72 (0·63) | 0·72 (0·62) |  | 0·58 (0·34) | 0·38 (0·33) |  | -0·20 | 0·05 to 0·62 | 0·024 | 0·67 |
|  |  | Depression |  |  | 1·75 (0·77) | 1·43 (0·80) |  | 1·67 (0·52) | 0·84 (0·62) |  | -0·83 | 0·18 to 1·01 | 0·0056 | 0·83 |
|  |  | Psychoticism |  |  | 0·50 (0·40) | 0·45 (0·43) |  | 0·54 (0·37) | 0·26 (0·27) |  | -0·30 | 0·02 to 0·43 | 0·030 | 0·64 |
|  |  | Paranoid Ideation | |  | 0·57 (0·46) | 0·53 (0·54) |  | 0·74 (0·64) | 0·35 (0·40) |  | -0·39 | -0·09 to 0·46 | 0·174 | 0·39 |
|  |  | Phobic Anxiety |  |  | 0·45 (0·52) | 0·33 (0·31) |  | 0·20 (0·27) | 0·11 (0·14) |  | -0·09 | 0·08 to 0·36 | 0·0031 | 0·91 |
|  |  | Global Severity Index | |  | 0·93 (0·44) | 0·80 (0·49) |  | 0·87 (0·33) | 0·50 (0·33) |  | -0·33 | 0·06 to 0·53 | 0·017 | 0·71 |
|  |  |  |  |  |  |  |  |  |  |  |  |  |  |  |
| **HAM-A** |  | Total score |  |  | 16·08 (7·28) | 12·58 (7·62) |  | 15·35 (5·66) | 7·58 (6·03) |  | -7·77 | 1·17 to 8·83 | 0·012 | 0·73 |
|  |  |  |  |  |  |  |  |  |  |  |  |  |  |  |
| **CGI** |  | Severity |  |  | 5·65 (0·89) | 5·19 (1·36) |  | 5·50 (0·58) | 3·88 (1·40) |  | -1·62 | 0·54 to 2·07 | 0·0012 | 0·95 |
|  |  |  |  |  |  |  |  |  |  |  |  |  |  |  |
| **CSSR-S** |  | Intensity |  |  | 0·54 (0·81) | 0·46 (0·95) |  | 0·50 (0·76) | 0·15 (0·37) |  | -0·35 | -0·10 to 0·71 | 0·13 | 0·43 |
|  |  |  |  |  |  |  |  |  |  |  |  |  |  |  |

The symptom checklist 90-revised (SCL-90-R) consists of nine subscales and one global index and included N=25 participants per condition; Hamilton-Anxiety Scale (HAM-A) is summarised as a total score; clinical global impressions (CGI) provides an indication-independent severity scale; Colombia-Suicidality severity rating scale (C-SSRS) was used to detect acute suicidality on a low-threshold by deriving intensity of ideation. Each questionnaire was administered at baseline five days before drug administration (except SCL-90-R, which was assessed during medical screening) and two weeks after the trial intervention. Mean differences from baseline, 95% confidence intervals, and P-values were derived using two-samples Welch’s t-test. Cohens’ *d* estimates effect sizes between treatment conditions at visit 7 (+14d).

### Supplemental Table 1. Statistical analysis of secondary endpoints.

|  | **Psilocybin** | **Placebo** |
| --- | --- | --- |
|  | (N=26) | (N=26) |
|  |  |  |
| **Headache** | 4 (15%) | 0 |
| resolved in  (mean days) | 1·75 |  |
| **Dizziness** | 2 (8%) | 0 |
| resolved in  (mean days) | 1·00 |  |
| **Nausea** | 1 (4%) | 0 |
| resolved in  (mean days) | 1·00 |  |
| **Diarrhea** | 1 (4%) | 0 |
| resolved in  (mean days) | 4·00 |  |
| **Common Cold** | 0 | 2 (8%) |
| resolved in  (mean days) |  | 2·50 |
| **Cystitis** | 0 | 1 (4%) |
| resolved in  (mean days) |  | 3·00 |
|  |  |  |

Adverse events reported from a total of N=52 participants over the course of the study, excluding acute, transient symptoms directly related to the well-known psychotropic effects of psilocybin. Causal relatedness to the active pharmaceutical compound administered and duration to completely resolve were rated for each adverse event.

### Supplemental Table 2. Overview of adverse events reported.
